# Spatial biology analysis reveals B cell follicles in secondary lymphoid structures may regulate anti-tumor responses at initial melanoma diagnosis

**DOI:** 10.3389/fimmu.2022.952220

**Published:** 2022-08-15

**Authors:** Aaron D. Therien, Georgia M. Beasley, Kristen E. Rhodin, Norma E. Farrow, Douglas S. Tyler, David Boczkowski, Rami N. Al-Rohil, Eda K. Holl, Smita K. Nair

**Affiliations:** ^1^ Department of Surgery, Duke University, Durham, NC, United States; ^2^ Department of Medicine, Duke University, Durham, NC, United States; ^3^ Department of Surgery, University Texas Medical Branch, Galveston, TX, United States; ^4^ Department of Pathology, Duke University, Durham, NC, United States; ^5^ Department of Neurosurgery, Duke University, Durham, NC, United States

**Keywords:** tumor immune microenvironment, B cells, digital spatial profiling, melanoma, sentinel lymph nodes (SLN)

## Abstract

**Introduction:**

B cells are key regulators of immune responses in melanoma. We aimed to explore differences in the histologic location and activation status of B cell follicles in sentinel lymph nodes (SLN) of melanoma patients.

**Methods:**

Flow cytometry was performed on fresh tumor draining lymph nodes (LN). Paraffin slides from a separate cohort underwent NanoString Digital Spatial Profiling (DSP)®. After staining with fluorescent markers for CD20 (B cells), CD3 (T cells), CD11c (antigen presenting cells) and a nuclear marker (tumor) was performed, regions of interest (ROI) were selected based on the location of B cell regions (B cell follicles). A panel of 68 proteins was then analyzed from the ROIs.

**Results:**

B cell percentage trended higher in patients with tumor in LN (n=3) compared to patients with nSLN (n=10) by flow cytometry. B cell regions from a separate cohort of patients with tumor in the (pSLN) (n=8) vs. no tumor (nSLN) (n=16) were examined with DSP. Within B cell regions of the SLN, patients with pSLN had significantly higher expression of multiple activation markers including Ki-67 compared to nSLN patients. Among 4 patients with pSLN, we noted variability in arrangement of B cell follicles which were either surrounding the tumor deposit or appeared to be infiltrating the tumor. The B cell follicle infiltrative pattern was associated with prolonged recurrence free survival.

**Conclusion:**

These data suggest a role for B cell follicles in coordinating effective adaptive immune responses in melanoma when low volume metastatic disease is present in tumor draining LN.

## Introduction

Recent reports suggest that tumors of melanoma patients that respond to immune checkpoint therapy have enriched B cell signatures (1). Formation of intratumoral tertiary lymphoid structures with B cells as part of the anti-tumor response has also been described (1, 2). Furthermore, T cells in tumors without tertiary lymphoid structures were found to have a dysfunctional molecular phenotype (1, 2). While B cells in patients with advanced melanoma are increasingly being recognized as critical to effective anti-tumor immune responses and durable disease control, B cells may also play an important role in regulating tumor progression in melanoma patients at the early stages of disease.

In the early stages, primary melanoma tumors first spread through lymphatics to the tumor draining lymph nodes (TDLN). Secondary lymphoid tissues, such as TDLN, represent the anatomic location that positions tumor in proximity to immune cells, including B cells. Further, lymph nodes are also the primary site where lymphocyte activation occurs (3). B-cell subtypes within lymph nodes can include either immunosuppressive or anti-tumor functions that subsequently contribute to differential immune responses (4). These tumor and immune cell interactions in lymph nodes can then result in either coordinated, effective adaptive immune responses or the tumor can induce lymph node remodeling which then drives immune escape and further tumor progression (3). In a mouse model of breast cancer, primary tumors were shown to induce B cell accumulation in TDLN and the B cells within the lymph node promoted further lymph node metastases (5). Regulatory B cells that accumulate in TDLN and promote tumor growth have also been described in a mouse model of melanoma (6). These data have motivated further investigation into the role of TDLN B cells in melanoma tumor progression (7–10).

At initial diagnosis, many patients with melanoma undergo evaluation of TDLN with a sentinel lymph node (SLN) biopsy. SLNs undergo histopathologic and immunohistochemical evaluations to detect any microscopic tumor in the lymph nodes. In melanoma patients, a higher number of CD20 B cells in the SLN compared to control lymph nodes has been reported (11). In a previous project, our group examined the immune profile of melanoma tumor draining lymph nodes from formalin fixed paraffin embedded blocks (FFPE) with the Nanostring nCounter® immune profiling panel. Using this method, the entire unstained slide (all portions of the lymph node) underwent analysis. We unexpectedly found that the expression of CD22 and PAX5, both genes involved in B cell signaling were significantly higher in patients with pSLN (n=19) compared to nSLN (n=19) (12). As such, we wanted to explore the potential contribution of B cell populations in tumor control in a more in-depth manner. We have previously described use of NanoString GeoMx digital spatial profiling (DSP) as a useful platform for studying human TDLN. Since DSP preserves lymph node architecture, we can identify and examine regions of B cell follicles separate from other portions of the lymph node. DSP can also provide in depth spatial analysis beyond just number and type of cells (13–15). We hypothesized that B cell follicle activation in TDLN at the time of initial melanoma diagnosis may in part regulate generation of initial effective anti-tumor immune responses or promote an immunosuppressive environment that permits tumor growth and metastasis. Herein, we aimed to further explore B cell regulation in melanoma TDLN at initial disease diagnosis.

## Materials and methods

All work was performed using Institutional Review Board (IRB) approved protocols. To obtain fresh SLN samples, patients undergoing SLN biopsy as part of routine clinical care were approached and consented for our melanoma biorepository (Pro00100644). Only patients with no clinically positive TDLN, found either upon palpation or imaging, prior to surgery were candidates for the sentinel node technique used to find microscopic tumor in TDLNs. Once obtained, SLNs had to be at least 1 cm in gross measurement to be included in the study. Immediately after surgical removal, a dermatopathologist evaluated the SLN grossly and provided a small portion of the SLN for flow cytometry not to jeopardize subsequent routine histopathology evaluation. This small portion of the SLN was transported to the laboratory, where immediate processing for flow cytometry was performed. Archived FFPE material for NanoString DSP was acquired from a separate group of patients who underwent SLNB for melanoma, did not receive immune checkpoint therapy nor targeted therapy, and had known clinical follow-up of at least 5 years using IRB protocol Pro00090678. SLNs had to be reported to be at least 1 cm in gross measurement to be included for DSP analysis.

### Flow cytometry

Sentinel lymph node samples were processed into single cell suspensions according to laboratory protocols. The SLN was enzymatically digested in a solution of RPMI-1640 containing enzymes from a human Tumor Dissociation Kit (Miltenyi Biotec, Bergisch Gladbach, Germany). The sample was processed in a gentleMACS™ Dissociator (Miltenyi Biotec, Bergisch Gladbach, Germany) and incubated at 37°C for 30 minutes. The suspensions were then filtered through a 70 µm filter to remove undigested connective tissue. Single cell suspensions were then incubated with the DuraClone IM Basic panel, which contains CD16-FITC, CD56-PE, CD19-ECD, CD14-PC7, CD4-APC, CD8-AF700, CD3-AF750, and CD45-KO as previously described, to identify immune cells in tissue (16). Additionally, propidium iodide (Sigma-Aldrich, St. Louis, MO) was added as a live-dead marker to SLN single cell suspensions. Following staining, cells were analyzed by flow cytometry on a 13-color CytoFlex flow cytometer (Beckman Coulter, Brea, CA). Representative gating strategy is shown in **Supplementary Figure 1**. Data were then analyzed on Kaluza software (Beckman Coulter, Brea, CA). Samples were stratified by the presence of metastatic tumor within the lymph node. Statistical analysis was performed in Prism9® (San Diego, CA) version 9.2.0 using a two-tailed Mann-Whitney test with a significance threshold of 0.05.

### NanoString digital spatial profiling

NanoString DSP is based on nCounter^®^ barcoding technology and enables spatially resolved, digital readout of proteins targets in a multiplexed assay. The assay relies upon antibody probes coupled to photocleavable oligonucleotide (oligo) tags. Detailed methods have been described (14, 15). Representative 5 µm thick sections were cut and a hematoxylin and eosin (H&E) stain performed before performing DSP. Using the H&E stain, a pathologist confirmed tumor and lymph node tissue present on the slide for pSLNs, and for nSLNs, a pathologist confirmed nodal tissue present on the slide. Corresponding unstained FFPE tissue slides were incubated with a cocktail of photo-cleavable-oligo-labeled primary antibodies (14, 15). The same slides were also fluorescently stained for CD3 (T cell), CD11c (likely dendritic cells), CD20 (B cells) and DNA (to identify cells and tumor) to visualize the morphology for regions of interest (ROI) selection. Once the incubation was complete, slides were loaded onto the DSP instrument and each sample was scanned to produce a digital image of tissue morphology based on the fluorescent markers. ROIs were then selected within geometric regions enriched for B cells or tumor. A programmable digital micromirror device (DMD) or dual-DMD (DDMD) directs UV light to precisely illuminate the ROI and cleave PC-oligos in a region-specific manner (14, 15). The released indexing oligos were then collected *via* microcapillary aspiration, dispensed into a microplate, and digitally counted using the single-molecule counting nCounter System (14, 15). A panel of 68 proteins including internal-spike controls were analyzed in selected ROIs. The protein panels were standard panels developed by Nanostring to include proteins associated with immune cell profiling and immune-oncology targets.

Protein expression values first underwent quality control analysis in the NanoString GeoMx DSP Analysis Suite® and was performed on 38 scans (3 patients had 3 scans, 8 patients had 2 scans, and 13 patients had 1 scan) and 417 segments/regions of interest (ROIs). To account and control for technical variability, a common set of external protein controls was utilized (17). In addition to ERCC (positive control) normalization, Quality Control also controlled for Field of View (FOV), Binding Density, Nuclei Count, and Surface Area (18). Eight ROIs failed positive control normalization and were excluded from further normalization and subsequent analysis. Further, 9 protein targets were removed from analysis as values were not present for all ROIs. The paired down dataset was analyzed using the *Evaluate Normalization for Protein Script* from the NanoString GeoMx Script Hub®. After following the algorithm, S6 and GAPDH were recommended as optimal normalization factors and the signal-to-noise ratio data was then used to compare protein expression between our selected ROIs. Values for all 42 proteins from the ROIs and the names of 42 proteins included in the final analysis are listed in **Supplementary Table 1**. Statistical analyses to compare protein expression were then performed using Prism9®. Kolmogorov-Smirnov test was used to compare medians between groups. The false-discovery Rate was set to 5%. Multiple comparisons were controlled for using the two-stage Benjamini, Krieger, and Yukitieli correction.

## Results

**Figure 1:**
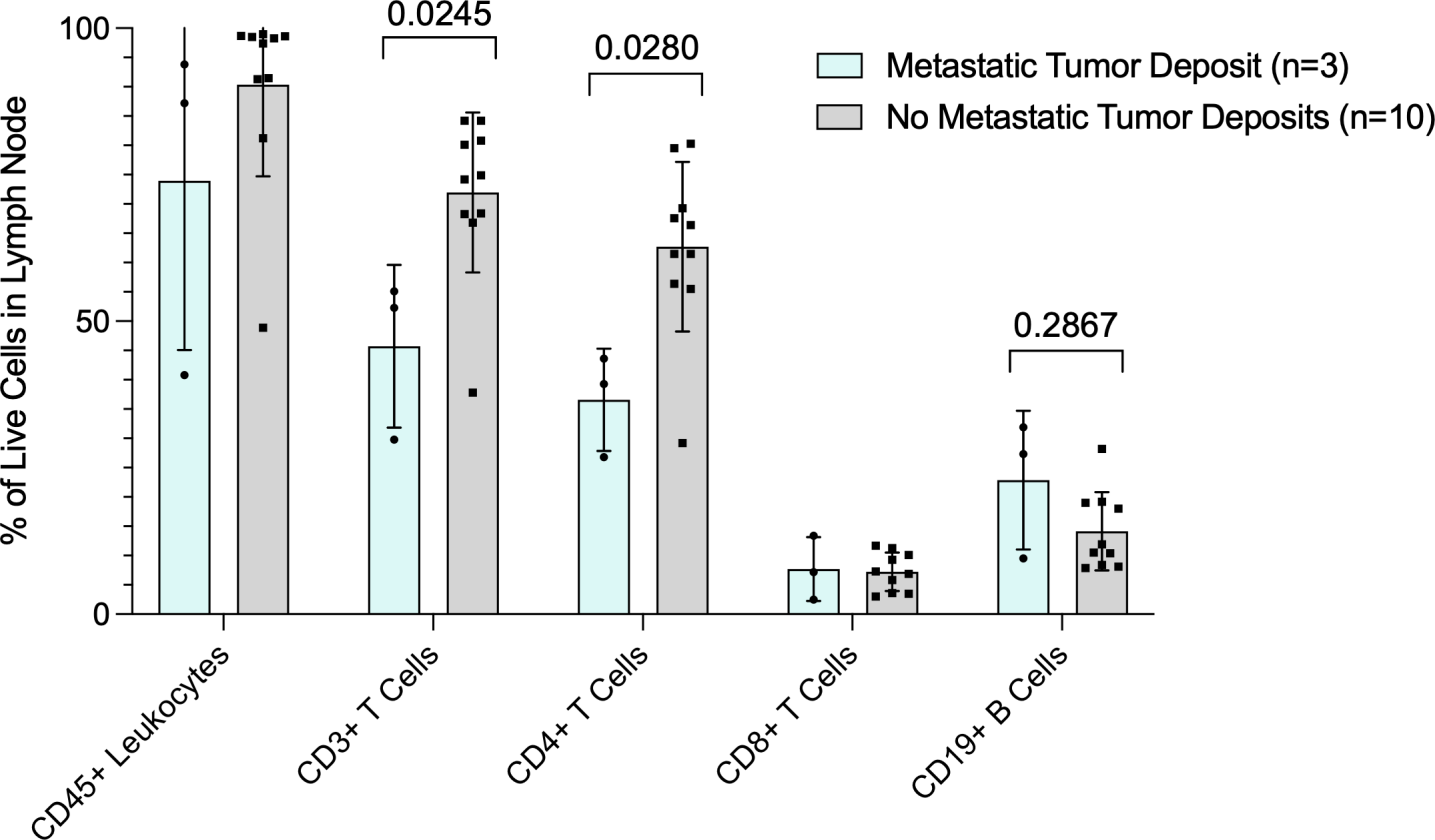
Flow cytometry analysis of fresh lymph node tissue. Data are presented as percent (%) of live cells in the lymph node (Y-axis). X axis shows cell subsets. Blue bars are patients with tumor in the lymph nodes and gray bars are patients with no tumor in the lymph nodes. P values for CD3+ T cell and CD19+ B cells are also shown.

We collected and analyzed fresh lymph node tissue prospectively from 13 patients undergoing surgery for melanoma. Three patients had tumor in the lymph node, including one patient with known tumor in the lymph node prior to surgery and two patients who were subsequently found to have microscopic tumor in the SLN (pSLN). The 3 patients with tumor in the lymph nodes had a significantly lower percentage of CD3+ and CD4+ T cells compared to patients (n=10) found to have no tumor in the SLN (nSLN) (p=0.0245 and p=0.028, respectively) (**Figure 1**). Additionally, 3 patients with tumor in the lymph nodes had a trend toward lower CD45+ leukocytes compared to nSLN patients (**Figure 1**). Despite overall lower CD3+ and CD4+ T cells, patients with pSLN (n=3) had a trend toward a higher percentage of B cells compared to patients with nSLN (n=10) (p=0.2867) (**Figure 1**).

To further investigate B cells in melanoma SLN, we obtained available FFPE sections from a separate retrospective cohort of patients undergoing SLN with at least 5 years of follow up, no receipt of immune checkpoint therapy nor targeted therapy. Prior to undergoing fluorescent staining, a pathologist confirmed presence of microscopic tumor where applicable (pSLN) on the slides. In total, 16 patients had a nSLN and 8 patients had a pSLN as reported in the routine clinically available histologic synopsis. Patient and tumor characteristics are listed in **Table 1**. Importantly, there was only 1 recurrence among the nSLN patients. Among pSLN patients, only 1 had received additional therapy for melanoma after surgery, as part of a vaccine trial. These patients had initial diagnosis prior to the era of modern systemic melanoma therapy and therefore none of these patients received immune checkpoint inhibitors or targeted therapy.

**Table 1:** Clinical characteristics of patients included in the DSP analysis.

Unique ID	Age (years)	Breslow depth (mm)	SLN Burden (mm)	Additional therapy after surgery	Recurrence	Overall Survival
SLN negative (n = 16)	49.5 median, range 31-80	1.3 Median	0.88-3.55 Range	No	1 of 16 at 8 years	Median 9.5 years
SLN P74	70	6	5	No	No	5.6 years
SLN P77	46	2.5	0.3	No	11 months	7.5 years
SLN P78	20	11	0.4	Vaccine trial	No	6 years
SLN P109	71	1.1	7	No	9 months	7 years
SLN P7	40	6.8	Not reported but EC was noted	No	3 months	Died 3.3 years
SLN P22	62	10.5	7.2	No	7 months	Died 6.9 years
SLN P23	49	2	5	No	6 years	12 years
SLN P47	53	2.8	3	No	No	10 years

SLN, sentinel lymph node; EC, extracapsular extension.

All slides underwent fluorescent staining for CD3 (T cell) in red, CD11c (likely dendritic cells) in green, CD20 (B cells) in gray and DNA (to identify cells and tumor) in blue.

**Figure 2:**
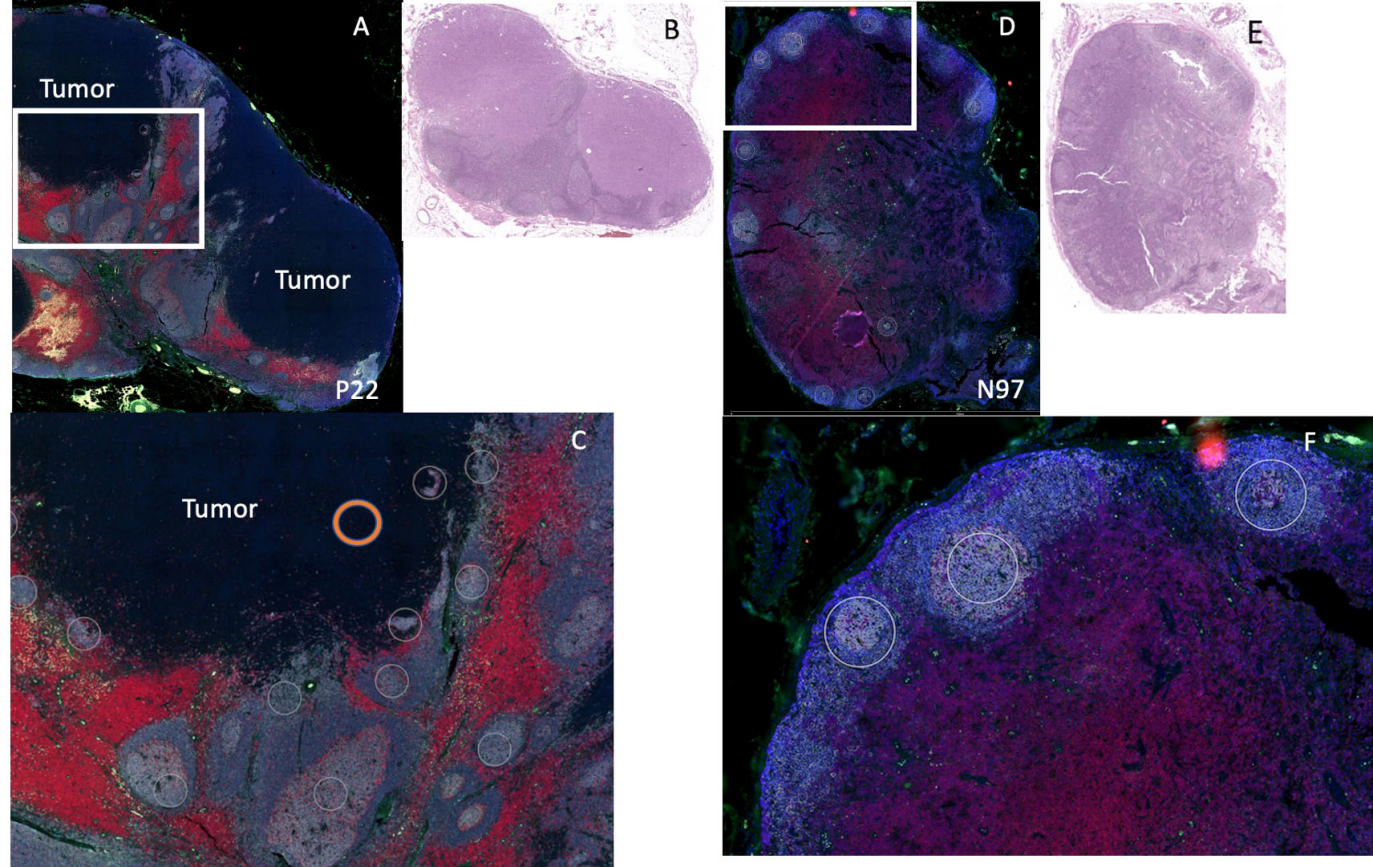
Example of DSP analysis. We used 2 patients for DSP analysis: patient P22 (positive sentinel node), left and patient N97 (negative sentinel lymph node), right. **(A)** Patient P22 SLN low power fluorescent staining for CD11c (green), CD20 (gray), CD3 (red), DNA (blue). Tumor region is labeled “tumor.” **(B)** H&E of sentinel lymph node for patient P22. **(C)** High power image of white rectangle from **(A)**, white circles indicate B cell regions selected for protein analysis (not all B cell regions selected are shown). Orange circle indicates an example of a tumor region chosen for protein analysis. **(D)** Patient N97, low power fluorescent staining for CD11c (green), CD20 (gray), CD3 (red), DNA (blue). **(E)** H&E of sentinel lymph node for patient N97. **(F)** High power image of white rectangle from **(D)**, white circles indicate B cell regions selected for protein analysis (not all B cell regions selected are shown).

After staining, geometric ROIs were selected based on the location of B cell follicles. B cell follicles were identified by using the comparison H&E and the fluorescent staining (CD20 cells are stained gray) as shown in **Figure 2**. On the fluorescent images, we noted the central portion of many B cell follicles appeared brighter, and using the H&E slides as comparison, these bright portions of the B cell follicles appeared to correspond to germinal centers on the H&E. Each B cell follicle is slightly different in total size. To keep the area of each ROI consistent for eventual analysis, (or use the same size circle for each ROI), we focused on selecting the brighter regions within the B cell follicles which were likely germinal centers. ROIs were also selected in regions where tumor only was present (**Figure 2C**); tumor was identified by comparing the DSP slide to the corresponding H&E slide as shown in **Figure 2**. An example of the ROI selection strategy is shown in **Figure 2**. On each slide, at least 10 ROIs for B cell rich regions were chosen and when possible, 2 or more regions of tumor were selected. ROIs then underwent analysis with the nCounter system and a panel of 68 proteins from the pre-specified ROIs were measured.

We first wanted to determine whether differences in B cell regions existed among patients with pSLN (n=8) and nSLN (n=16). We did note that the B cell follicles near tumor from patients with pSLN appeared to be larger and less well circumscribed compared to patients with nSLN (**Figure 2**). We then compared a total of 290 B cell ROIs from patients with nSLN (16 patients with minimum 10 regions each), to 115 B cell ROIs from patients with pSLN (8 patients with minimum 10 regions each). From the panel of 68 proteins, we excluded 6 controls as well as 20 proteins that fell below the lowest control median. Of the remaining 42 proteins, expression of 29 were significantly different between the 2 groups (pSLN versus nSLN) as listed in **Table 2**. As expected, MART-1, a melanoma marker was higher in patients with a pSLN. The B cell regions in patients with pSLN had a pattern of expression consistent with a higher level of activation, proliferation, including higher expression of Ki-67, CD45, CD40, HLA-DR, CD44, CD8, CD4, and CD68 as shown in **Table 2**. Interestingly, the only protein which was higher in nSLN patients compared to pSLN was Bcl-2 (**Table 2**).

**Table 2:** Median values of proteins significantly different between B cell regions from patients with no tumor in the sentinel lymph node (nSLN), compared to patients with tumor in the SLN (pSLN).

	nSLN Median	pSLN Median	q value
CD68	585.4	943.3	<0.000001
PanCk	156.8	210.9	<0.000001
CD11c	219.4	280.1	<0.000001
CD56	223.64	307.1	<0.000001
CD8	437.9	638.4	<0.000001
CD20	136.8	194.7	<0.000001
CD4	466.2	676.9	0.000011
CD3	532.9	801.6	0.000011
NY-ESO-1	52.8	64.4	0.000016
CD27	85.5	117.9	0.000073
PD-1	233.8	308.5	0.000092
CD127	179.2	204.9	0.000095
Beta-2-microglobulin	281.2	361.1	0.000118
CD45RO	172.7	198.4	0.000224
CD14	124.7	174.7	0.000384
pan-RAS	39.5	51.6	0.000732
HLA-DR	2550.3	3078.4	0.001159
Ki-67	1356.2	2372.7	0.001159
Bcl-2	836.3	542.8	0.001159
CD40	70.0	90.9	0.001315
CD25	55.6	64.1	0.0016
Phospho-c-RAF (S338)	40.4	45.7	0.001982
CD45	6033.6	7662.2	0.002494
MART1	64.6	79.2	0.003097
CD44	1090.9	1763.5	0.005461
VISTA	78.1	96.5	0.010158
p44/42 MAPK ERK1/2	490.2	511.3	0.016366
CD34	119.0	141.2	0.022224
SMA	2276.1	2645.9	0.025154

**Figure 3:**
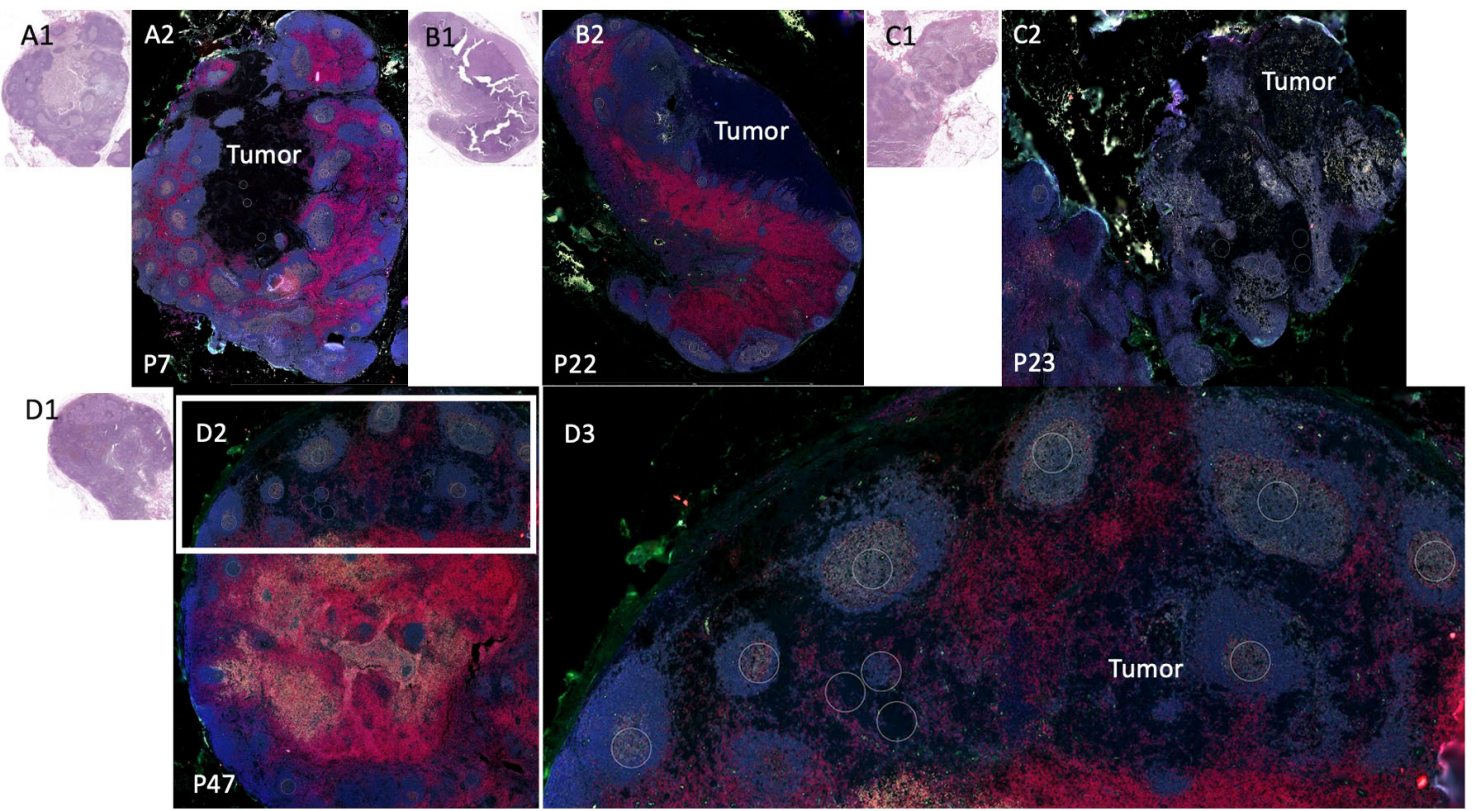
Sentinel lymph node tissue analysis. Comparison of sentinel lymph node tissue (SLN) from patients P22, P23, P7, (top) and P47 (bottom). Fluorescent antibodies were used to examine CD11c (green), CD20 (gray), CD3 (red), and DNA (blue.) **(A1)** Patient P7 SLN H&E. **(A2)** Patient P7 stained SLN. **(B1)** Patient P22 SLN H&E **(B2)** Patient P22 (an additional cut separate from **Figure 2**) stained SLN. **(C1)** Patient P23 SLN H&E. **(C2)** Patient P23 SLN stained. **(D1)** Patient P47 SLN H&E. **(D2)** Patient P47 SLN stained low power. **(D3)** Patient P47 SLN high power of white rectangle from **(D2)**.

Among the 8 patients with pSLN confirmed by a pathologist on the corresponding H&E stain, regions of microscopic tumor on the fluorescently marked slides were only clearly visible in 4 patients. Among those without visible tumor on DSP images, 2 patients had known tumor burden less than 0.5 mm and we could not reliably discern the tumor on the fluorescent images. In 2 other cases, the tumor regions were not scanned into the region analyzed with DSP. We next focused on the 4 patients with visible tumor on the slide (**Figure 3**). First, we noted a striking difference in the B cell arrangement among these 4 patients. For patient number 47 (**Figure 3D**), we noted that both B cells (gray) and T cells (red) appeared to have infiltrated the tumor whereas for the other 3 patients (patients 7, 22, 23), the B cell follicles were stacked along the periphery of the tumor, or the tumor appeared to be immune cell excluded (**Figures 3A–C** respectively). Interestingly, patient 47 (infiltrative pattern) has had no melanoma recurrence at 10 years while patients 7, 22, 23 with an immune excluded tumor pattern experienced recurrence at 3 months, 7 months, and 6 years respectively. Patients 7 and 22 also died from disease at 3.3 and 6.9 years (**Table 1**).

**Figure 4:**
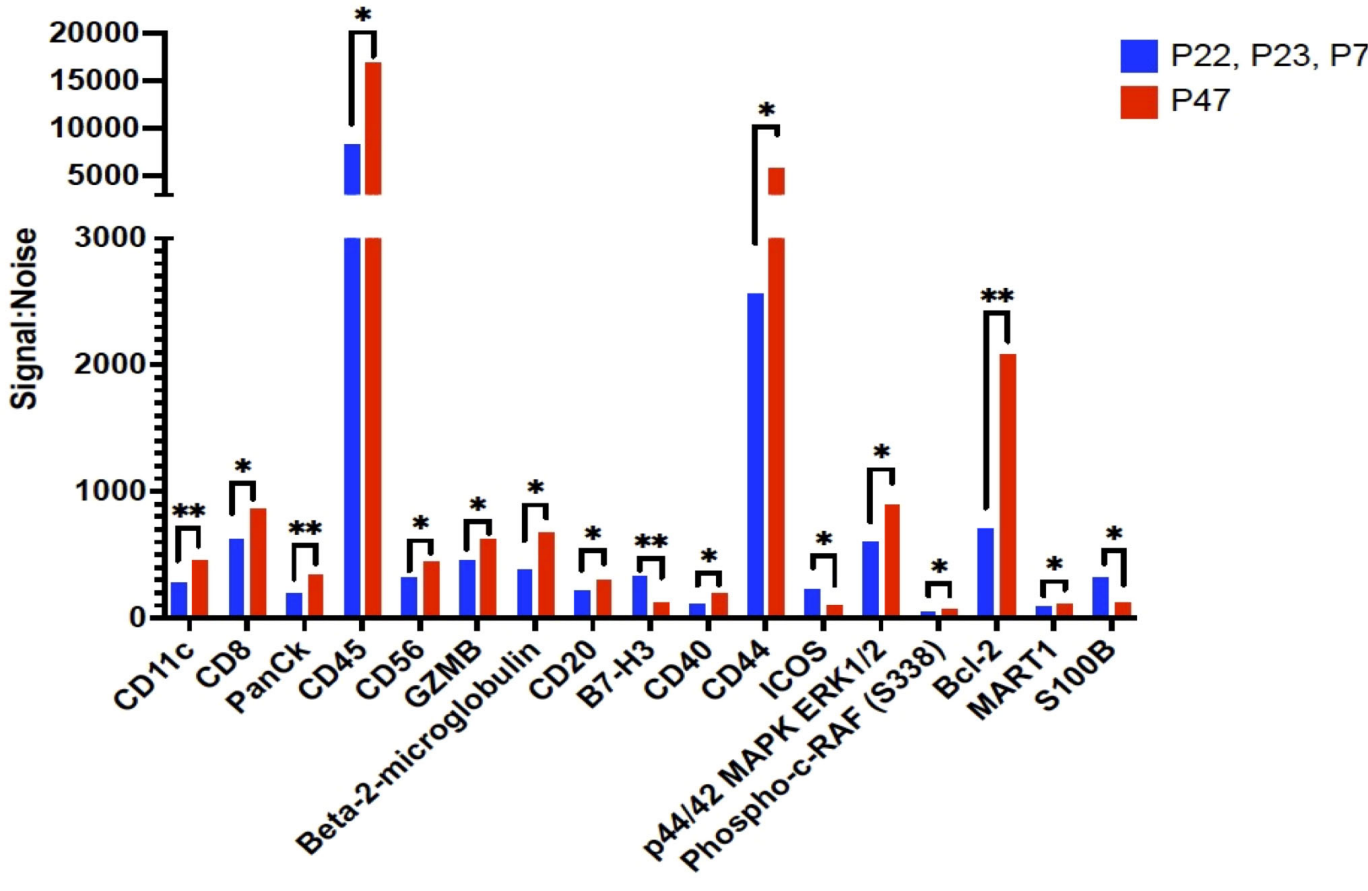
Proteins that were significantly differentially expressed between B cell regions among patients with tumor in the sentinel lymph nodes. The signal to noise ratio (y axis) of protein levels from patients P22, P23, and P7 with shorter recurrence free survival in blue bars compared to protein expression from B cell regions from patient P47 in red bars. Protein target names are listed on the x axis. *= q value less than 0.05, **=q value < 0.01; q value adjusted for false discovery rate set at 5%.

We then examined B cells regions (n=53 ROIs) from the 3 patients where B cell follicles were surrounding the tumor compared to regions (n=10 ROIs) from patient 47 where B cells had infiltrated the tumor (**Figure 4**). Of the 42 proteins included in analysis, 17 proteins were significantly higher in B cells clusters from patient 47 including CD8, CD45, GZMB, and Bcl-2 compared to the other 3 patients (excluded pattern) (**Figure 4**). In contrast, B7-H3 and ICOS were significantly higher in B cell regions of the 3 patients with poor prognosis and the spatial immune exclusion pattern compared to the patient with the infiltrative pattern (**Figure 4**).

**Figure 5:**
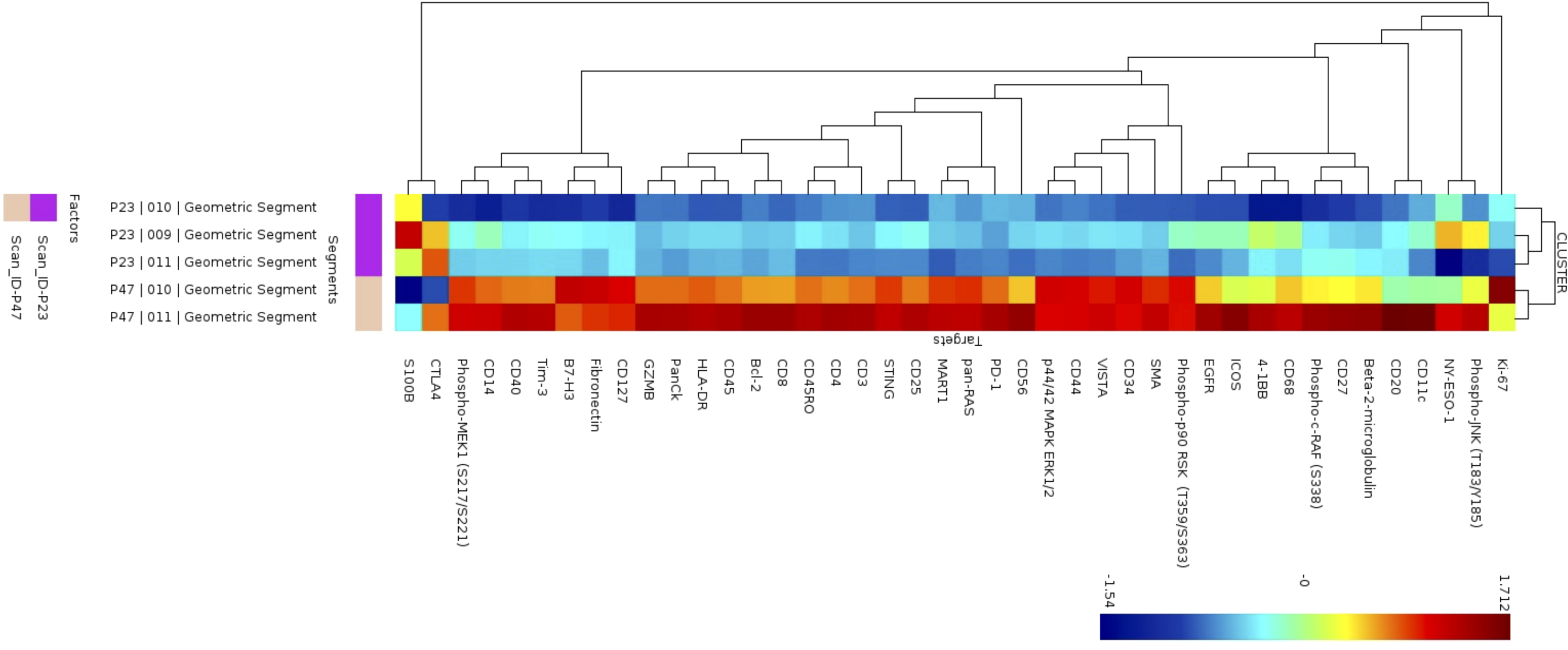
Cluster analysis of protein expression in B cell regions. Cluster analysis of protein expression from 3 B cell regions from patient P23 (purple) and 2 B cells regions from patient P47 (tan).

In 2 patients, patient 47 and patient 23, we had selected ROIs for analysis on the tumor region of the slide to determine differences in the tumor that may account for ability of immune cells to infiltrate the tumor portion of the lymph node. To explore heterogeneity between tumor regions (P23 and P47), cluster analysis was performed in the NanoString GeoMx DSP Analysis Suite®. The S6-GAPDH normalized signal-to-noise data for the tumor ROIs (3 regions for patient P23 and 2 regions for patient P47) were examined. The corresponding tumor ROI cluster data was log-transformed and visualized using z-scored values, where each segment-probe cell color represents a z-score. Of 42 proteins, the tumors appeared to have different expression of multiple proteins as shown in the heatmap in **Figure 5**. Patient 47’s tumor had higher levels of 4-1BB, CD3, CD45, HLA-DR, and Ki-67 compared to patient 23.

## Discussion

The detection of microscopic melanoma in lymph nodes (pSLN) is associated with worse disease outcomes compared to patients without tumor spread to lymph nodes (nSLN) (19). However, the outcomes for patients with tumor in the SLN are highly variable with some patients achieving long term disease control, while other patients can rapidly progress and develop distant metastatic disease. Standard histopathologic evaluation includes the number of SLN containing tumor metastasis, the size of SLN tumor deposits, and presence of extracapsular tumor extension (20). While these practices can certainly assist in identifying patients at higher risk of recurrence, standard measurement and evaluation of the tumor immune response is lacking and as a result, many patients are inappropriately risk stratified (21). Precise risk stratification has become increasingly important in the selection of patients who may benefit from adjuvant therapy after initial surgery which can reduce rates of recurrence. However, these therapies can have significant toxicity and should only be given to patients at high risk of recurrence (22). Therefore, we have begun evaluation of SLN to assess not only standard histopathology but also immunologic function which has potential to provide insight into mechanisms of tumor progression (13, 23). Here, we demonstrate a trend toward greater proportions of B cells among the immune infiltrate in pSLN compared to nSLN. Further, the characteristics of B cell follicles appear to be very different in patients with pSLN compared to nSLN. Moreover, among patients with pSLN, there may be key differences in B cell follicle orientation and activation status that can differentiate effective control of tumor growth from continued tumor progression.

B cell accumulation in tumor draining lymph nodes that can drive the remodeling that precedes and promotes metastasis has been reported in animal models of melanoma and breast cancer (5, 6). Conferring these findings in human models is difficult. Although many patients undergo evaluation of TDLN, the lymph node must be processed for histopathologic evaluation as a key guide for clinical care. Working with our pathologist, we obtained a small portion of a fresh SLN without compromising histopathology and performed basic analysis using flow cytometry to determine immune cell presence and distributions. Although this technique can provide initial immune characterization, this analysis disrupts lymph node architecture and reduces the number of cells available for additional experiments. NanoString DSP on paraffin sections preserves lymph node architecture and only requires 5-6 µm slices of tissue from paraffin tissue. Paraffin tissue is the most readily accessible patient tissue in the clinic. Hence, DSP may be an ideal platform to study the immunologic profile of TDLN and host-immune interactions at the time of initial melanoma diagnosis. However, the technique does have some limitations. For example, some nSLN tissue expressed low levels of MART-1. MART-1 expression in lymph nodes without tumor (false positive) has been described and likely accounts for some of the MART-1 expression in nSLN (24, 25). MART-1 was significantly higher in pSLN compared to nSLN.

We observed from the images that B cell rich regions in patients with pSLN appeared larger, less well circumscribed, and more centrally located than the peripheral, symmetric B cell follicles of patients with nSLN, suggesting gross morphologic changes in the presence of tumor. There were also differences in immunologic protein expression of B cell rich regions between patients with pSLN and nSLN; 29 of 42 proteins examined were significantly different which suggests B cells in the TDLN are reactive to tumor. Many of these proteins (Ki-67, CD40, HLA-DR) are important in B cell activation during germinal center formation (26). Bcl-6, an essential marker of the germinal center activation pathway was not included in the protein panel, but will be important to further examine possible tumor induced B cell activation through the germinal center pathway (27). Next, we noted interesting patterns of B cell follicle orientation in 4 patients with tumor in the SLN. Notably the traditionally prognostic histopathologic features of primary melanomas (Breslow depth, SLN tumor burden) were similar among the 4 patients. In 3 patients, all who developed recurrence, the B cell follicles appeared to be stacked or excluded along the border of the tumor. In 1 patient with no recurrence at 10 years, B cells and CD3+ cells appeared on DSP images to have infiltrated the tumor portion of the SLN. These distinct patterns of tumor immune cell infiltration or exclusion have been well described in metastatic melanoma tumors, where an infiltrative pattern is known to correlate with overall prognosis independent of therapy and is, in fact, predictive of response to checkpoint therapy (28, 29). Thus, preserving the spatial arrangement of cells in the SLN may be important in assessing effectiveness of immunologic responses. Further, spatial analysis may provide additional insight into risk stratification, appropriateness of adjuvant therapy, and possible response to therapy.

In addition to location, we noted differences in protein expression of B cell follicles excluded from (3 patients) and infiltrating (1 patient) the tumor regions of SLN. The excluded B cells appeared to be immunosuppressive with higher levels of B7-H3, a known immune checkpoint, and higher ICOS (Inducible T cell Costimulator), known to have immunosuppressive activities mediated by regulatory T cells (30, 31). Notably, the infiltrative B cell follicles showed higher markers of proteins associated with effective anti-tumor immune responses (CD8, Granzyme B) (32) (33). Finally, we noted marked differences in immunologic protein expression from tumor regions of 2 patients with divergent outcomes including one with an infiltrative pattern and 1 with an excluded pattern, which may account for ability of B cells and other immune cells to infiltrate the tumor.

Although limited by sample size, our data are consistent with findings in animal models where B cells appear to accumulate in TDLN when tumor is present. In some cases, the B cell arrangement and protein expression appears to support tumor progression whereas in 1 patient the arrangement and protein expression may be suggestive of an anti-tumor response, given the patient’s prolonged survival. This dichotomy may explain in part why the role of B cells in tumor progression have been inconsistent in the literature with both pro- and anti-tumor activity reported (8, 10, 34). Additionally, these types of data may help us better prognosticate which patients are at a higher risk for development of further tumor progression.

Mounting evidence suggests that B cells likely play an important role at early stages of melanoma progression, especially in tumor draining lymph nodes where B cell regulation can coordinate effective adaptive immune responses or promote lymph node remodeling and tumor progression. We explored B cells in tumor draining lymph nodes with spatial profiling, a powerful adjunct to understand the complex cellular and proteomic relationships within the highly variable melanoma tumor immune environment. We propose that spatial assessment of immunologic response as an adjunct to histopathologic evaluation may ultimately help refine patient selection for additional therapy at the time of initial melanoma diagnosis and reduce further tumor progression.

## Data availability statement

The original contributions presented in the study are included in the article/**Supplementary Material**. Further inquiries can be directed to the corresponding author/s.

## Ethics statement

The studies involving human participants were reviewed and approved by Duke Health Institutional Review Board. The ethics committee waived the requirement of written informed consent for participation.

## Author contributions

Study conception and design: GB, SN, and EH. Data collection and analysis: AT, GB, EH, NF, KR, and DB. Analysis and interpretation: AT, GB, EH, NF, KR, DT, DB, and SN. Manuscript draft preparation: GB, AT, and KR. All authors reviewed the results and approved the final version of the manuscript.

## Funding

The work in this paper was supported by Department of Defense Award Number: W81XWH-20-1-0808 (PI, EH co-I, GB). GB is supported by NIH K08 CA237726-01A1. KR is supported by NIH T32-CA093245-15 for translational research in surgical oncology. GB has clinical trial funding from Istari Oncology, Delcath, Oncosec Medical, Replimune, and Checkmate Pharmaceuticals paid to Duke University. The funders were not involved in the study design, collection, analysis, interpretation of data, the writing of this article or the decision to submit it for publication.

## Acknowledgments

We thank our pathologists and pathology assistants for their availability and assistance in the handling of fresh samples.

## Conflict of interest

The authors declare that the research was conducted in the absence of any commercial or financial relationships that could be construed as a potential conflict of interest.

## Publisher’s note

All claims expressed in this article are solely those of the authors and do not necessarily represent those of their affiliated organizations, or those of the publisher, the editors and the reviewers. Any product that may be evaluated in this article, or claim that may be made by its manufacturer, is not guaranteed or endorsed by the publisher.

## References

[1] Cabrita R Lauss M Sanna A Donia M Skaarup Larsen M Mitra S. Tertiary lymphoid structures improve immunotherapy and survival in melanoma. Nature (2020) 577:561–5. doi: 10.1038/s41586-019-1914-8 31942071

[2] Helmink BA Reddy SM Gao J Zhang S Basar R Thakur R. B cells and tertiary lymphoid structures promote immunotherapy response. Nature (2020) 577:549–55. doi: 10.1038/s41586-019-1922-8 PMC8762581 31942075

[3] Krishnamurty AT Turley SJ. Lymph node stromal cells. cartographers of the immune system. Nat Immunol (2020) 21:369–80. doi: 10.1038/s41590-020-0635-3 32205888

[4] Sarvaria A Madrigal JA Saudemont A. B cell regulation in cancer and anti-tumor immunity. Cell Mol Immunol (2017) 14:662–74. doi: 10.1038/cmi.2017.35 PMC5549607 28626234

[5] Gu Y Liu Y Fu L Zhai L Zhu J Han Y. Tumor-educated b cells selectively promote breast cancer lymph node metastasis by HSPA4-targeting IgG. Nat Med (2019) 25:312–22. doi: 10.1038/s41591-018-0309-y 30643287

[6] Ganti SN Albershardt TC Iritani BM Ruddell A. Regulatory b cells preferentially accumulate in tumor-draining lymph nodes and promote tumor growth. Sci Rep (2015) 5:12255. doi: 10.1038/srep12255 26193241 PMC4507466

[7] Willsmore ZN Harris RJ Crescioli S Hussein K Kakkassery H Thapa D. B cells in patients with melanoma. implications for treatment with checkpoint inhibitor antibodies. Front Immunol (2021) 11:622442. doi: 10.3389/fimmu.2020.622442 33569063 PMC7868381

[8] Somasundaram R Zhang G Fukunaga-Kalabis M Perego M Krepler C Xu X. Tumor-associated b-cells induce tumor heterogeneity and therapy resistance. Nat Commun (2017) 8:607. doi: 10.1038/s41467-017-00452-4 28928360 PMC5605714

[9] Lynch KT Young SJ Meneveau MO Wages NA Engelhard VH Slingluff CL Jr.. Heterogeneity in tertiary lymphoid structure b-cells correlates with patient survival in metastatic melanoma. J Immunother Cancer (2021) 9:1–10. doi: 10.1136/jitc-2020-002273 PMC8190052 34103353

[10] Chen M Werner F Wagner C Simon M Richtig E Mertz KD. Spatiotemporal analysis of b cell- and antibody secreting cell-subsets in human melanoma reveals metastasis-, tumor stage-, and age-associated dynamics. Front Cell Dev Biol (2021) 9:677944. doi: 10.3389/fcell.2021.677944 34095149 PMC8176028

[11] Grotz TE Jakub JW Mansfield AS Goldenstein R Enninga EA Nevala WK. Evidence of Th2 polarization of the sentinel lymph node (SLN) in melanoma. Oncoimmunology (2015) 4:e1026504. doi: 10.1080/2162402X.2015.1026504 26405583 PMC4570120

[12] Beasley GM Holl EK Farrow NE Leddy M Salama AK Hanks BA. (2019). The immune profile of sentinel nodes in melanoma, in: AACR Special Conference on Melanoma, From Biology to Target. Houston, Texas

[13] Beasley GM Therien AD Holl EK Al-Rohil R Selim MA Farrow NE. Dissecting the immune landscape of tumor draining lymph nodes in melanoma with high-plex spatially resolved protein detection. Cancer Immunol Immunother (2021) 70:475–83. doi: 10.1007/s00262-020-02698-2 PMC7892641 32814992

[14] Beechem JM. High-plex spatially resolved RNA and protein detection using digital spatial profiling. a technology designed for immuno-oncology biomarker discovery and translational research. Methods Mol Biol (2020) 2055:563–83. doi: 10.1007/978-1-4939-9773-2_25 31502169

[15] Toki MI Merritt CR Wong PF Smithy JW Kluger HM Syrigos KN. High-plex predictive marker discovery for melanoma immunotherapy-treated patients using digital spatial profiling. Clin Cancer Res (2019) 25:5503–12. doi: 10.1158/1078-0432.CCR-19-0104 PMC6744974 31189645

[16] Holl EK Frazier VN Landa K Beasley GM Hwang ES Nair SK. Examining peripheral and tumor cellular immunome in patients with cancer. Front Immunol (2019) 10:1767. doi: 10.3389/fimmu.2019.01767 31417550 PMC6685102

[17] NanoString IG. nCounter data analysis user manual. (2020) https://nanostring.com/support-documents/ncounter-advanced-analysis-2-0-user-manual/.

[18] Incorporated N. Nanonstring Inc. GeoScript hub (2021). Available at: https://www.nanostring.com/products/geomx-digital-spatial-profiler/geoscript-hub/.

[19] Morton DL Thompson JF Cochran AJ Mozzillo N Nieweg OE Roses DF. Final trial report of sentinel-node biopsy versus nodal observation in melanoma. N Engl J Med (2014) 370:599–609. doi: 10.1056/NEJMoa1310460 24521106 PMC4058881

[20] Egger ME Bower MR Czyszczon IA Farghaly H Noyes RD Reintgen DS. Comparison of sentinel lymph node micrometastatic tumor burden measurements in melanoma. J Am Coll Surg (2014) 218:519–28. doi: 10.1016/j.jamcollsurg.2013.12.014 24491245

[21] Masoud SJ Perone JA Farrow NE Mosca PJ Tyler DS Beasley GM. Sentinel lymph node biopsy and completion lymph node dissection for melanoma. Curr Treat Options Oncol (2018) 19:55. doi: 10.1007/s11864-018-0575-4 30232648 PMC6684152

[22] Kwak M Farrow NE Salama AKS Mosca PJ Hanks BA Slingluff CL. Updates in adjuvant systemic therapy for melanoma. J Surg Oncol (2019) 119:222–31. doi: 10.1002/jso.25298 PMC6330126 30481375

[23] Farrow NE Holl EK Jung J Gao J Jung SH Al-Rohil RN. Characterization of sentinel lymph node immune signatures and implications for risk stratification for adjuvant therapy in melanoma. Ann Surg Oncol (2021) 28:3501–10. doi: 10.1245/s10434-020-09277-w PMC8126577 33205334

[24] Brennick JB Yan S. False-positive cells in sentinel lymph nodes. Semin Diagn Pathol (2008) 25:116–9. doi: 10.1053/j.semdp.2008.03.001 18697715

[25] Yan S Brennick JB. False-positive rate of the immunoperoxidase stains for MART1/MelanA in lymph nodes. Am J Surg Pathol (2004) 28:596–600. doi: 10.1097/00000478-200405000-00005 15105646

[26] Vaidehi Narayanan H Hoffmann A. From antibody repertoires to cell-cell interactions to molecular networks. bridging scales in the germinal Center.0. Front Immunol (2022) 13:898078. doi: 10.3389/fimmu.2022.898078 35603162 PMC9114758

[27] Vinuesa CG Linterman MA Goodnow CC Randall KL. T Cells and follicular dendritic cells in germinal center b-cell formation and selection. Immunol Rev (2010) 237:72–89. doi: 10.1111/j.1600-065X.2010.00937.x 20727030

[28] Joyce JA Fearon DT. T Cell exclusion, immune privilege, and the tumor microenvironment. Science (2015) 348:74–80. doi: 10.1126/science.aaa6204 25838376

[29] Erdag G Schaefer JT Smolkin ME Deacon DH Shea SM Dengel LT. Immunotype and immunohistologic characteristics of tumor-infiltrating immune cells are associated with clinical outcome in metastatic melanoma. Cancer Res (2012) 72:1070–80. doi: 10.1158/0008-5472.CAN-11-3218 PMC3306813 22266112

[30] Flem-Karlsen K Fodstad Ø Nunes-Xavier CE. B7-H3 immune checkpoint protein in human cancer. Curr Med Chem (2020) 27:4062–86. doi: 10.2174/0929867326666190517115515 31099317

[31] Amatore F Gorvel L Olive D. Role of inducible Co-stimulator (ICOS) in cancer immunotherapy. Expert Opin Biol Ther (2020) 20:141–50. doi: 10.1080/14712598.2020.1693540 31738626

[32] Kontos F Michelakos T Kurokawa T Sadagopan A Schwab JH Ferrone CR. B7-H3. an attractive target for antibody-based immunotherapy. Clin Cancer Res (2021) 27:1227–35. doi: 10.1158/1078-0432.CCR-20-2584 PMC7925343 33051306

[33] Solinas C Gu-Trantien C Willard-Gallo K. The rationale behind targeting the ICOS-ICOS ligand costimulatory pathway in cancer immunotherapy. ESMO Open (2020) 5:1–6. doi: 10.1136/esmoopen-2019-000544 PMC7003380 32516116

[34] Griss J Bauer W Wagner C Simon M Chen M Grabmeier-Pfistershammer K. B cells sustain inflammation and predict response to immune checkpoint blockade in human melanoma. Nat Commun (2019) 10:4186. doi: 10.1038/s41467-019-12160-2 31519915 PMC6744450

