# Supplementary material for: Spatial biology analysis reveals B cell follicles in secondary lymphoid structures may regulate anti-tumor responses at initial melanoma diagnosis
Source: Front Immunol. 2022 Aug 15;13:952220. doi: 10.3389/fimmu.2022.952220 (PMC9425113; doi:10.3389/fimmu.2022.952220)
Supplement: Supplementary file 1 [file DataSheet_1.pdf]

Supplementary Material

Spatial Biology Analysis Reveals B Cell Follicles in Secondary Lymphoid Structures May Regulate Anti- tumor Responses at Initial Melanoma Diagnosis

Aaron D Therien, MS[1], Georgia M Beasley, MD[1,2], Kristen E Rhodin, MD[1], Norma E Farrow, MD[1], Douglas S Tyler, MD[3], David Boczkowski, MS[1], Rami N Al-Rohil, MBBS[4], Eda K Holl, PhD[1] and Smita K Nair, PhD[1,4,5]

1. Duke University, Department of Surgery
2. Duke University, Department of Medicine
3. University Texas Medical Branch, Department of Surgery
4. Duke University, Department of Pathology
5. Duke University, Department of Neurosurgery

Co-Corresponding authors:
Eda K Holl, PhD
Duke University School of Medicine, Box 103035, Durham, NC 27710 Email: eda.holl@duke.edu

Smita K Nair, PhD
Duke University School of Medicine, Box 103035, Durham, NC 27710 Email: smita.nair@duke.edu

Supplementary Figure 1

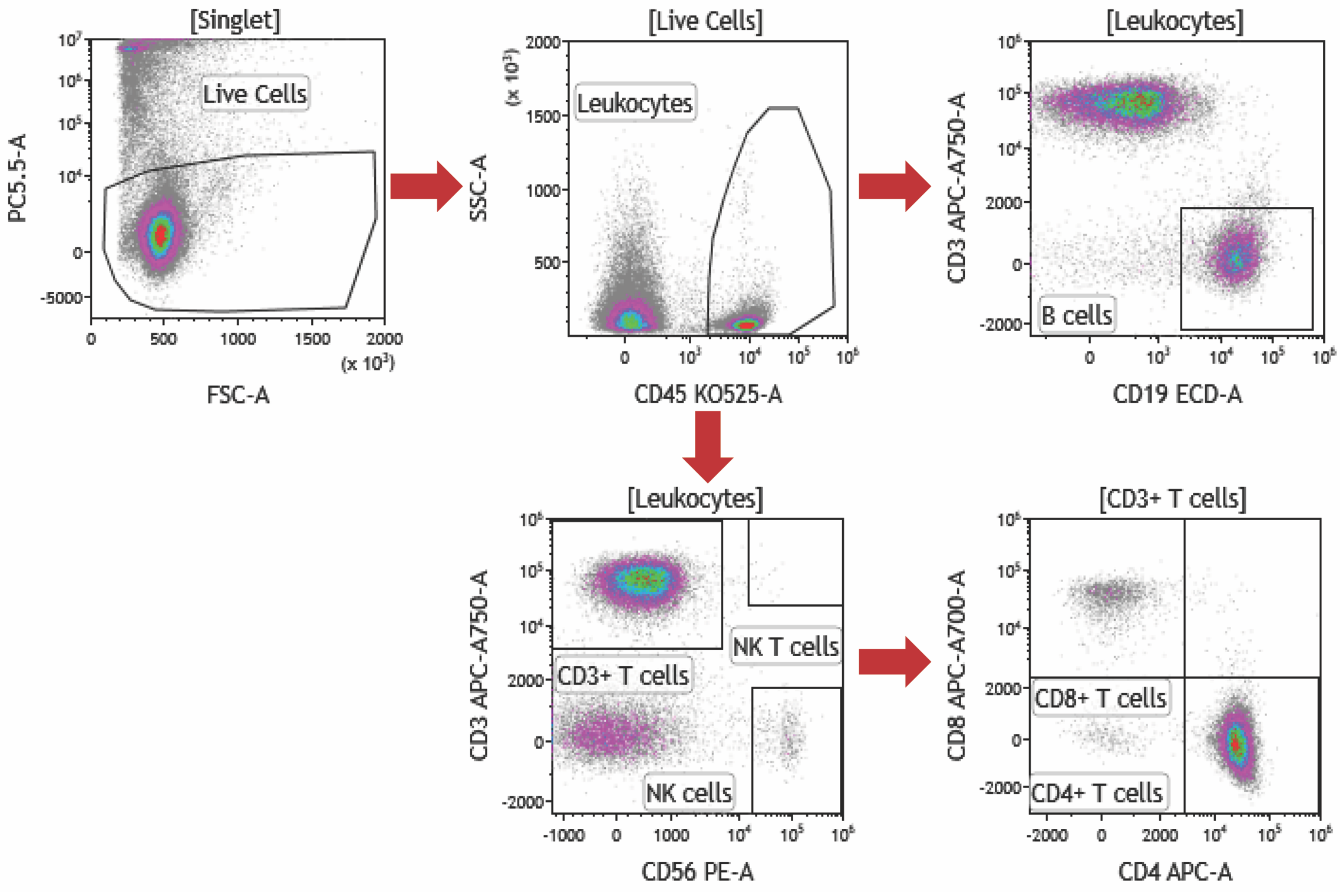

Figure S1. Flow cytometry gating strategy used to analyze T cells and B cells in the sentinel lymph node. Flow cytometry gating strategy for manuscript Figure 1. Sentinel lymph node tissue was digested and gated on live cells (propidium iodide (PI)-negative). Leukocytes were identified based on expression of CD45. Immune cell subsets were separated based on expression of surface markers (CD19, CD3, CD56, CD4, and CD8). T and B cells were identified based on expression of CD3 (T cells) and CD19 (B cells). NK cells were defined as CD56+ and CD3– and NK T cells were defined as CD56+ and CD3+. CD3+ T cells were separated into CD4+ T cells and CD8+ T cells.
