# Supplementary material for: Spatial biology analysis reveals B cell follicles in secondary lymphoid structures may regulate anti-tumor responses at initial melanoma diagnosis
Source: Front Immunol. 2022 Aug 15;13:952220. doi: 10.3389/fimmu.2022.952220 (PMC9425113; doi:10.3389/fimmu.2022.952220)
Supplement: Supplementary file 2 [file DataSheet_2.pdf]

	N103 N1 | N103 N1 001 | Geometric Segment	N103 N1 | N103 N1 002 | Geometric Segment
PD-1	503.346351	196.153563
Fibronectin	686.120817	596.354336
CD11c	211.242246	173.936934
CD8	250.84937	349.236047
CD4	400.178296	232.342932
CD3	499.7151	247.782901
PanCk	139.430726	121.323395
SMA	2203.32835	1682.49477
CD45	3605.82005	4411.95339
CD56	145.771228	120.922973
CTLA4	107.223583	45.1966841
HLA-DR	3115.53063	3584.51508
Ki-67	3902.43511	3208.27082
GZMB	557.740032	305.508953
CD68	682.119109	575.219483
Beta-2-microglobulin	193.235661	191.818485
CD20	116.059838	99.907513
Tim-3	39.5005441	17.3297953
B7-H3	174.29332	207.415326
VISTA	95.1492119	60.8768754
4-1BB	815.185699	584.146438
STING	580.91693	142.876952
CD40	52.4233128	47.7022737
CD127	174.649977	213.551009
CD44	211.181109	259.934721
ICOS	262.301599	134.257354
CD27	108.677799	64.5261821
CD25	59.4622916	44.2696496
CD14	57.2173569	78.847809
CD34	127.631461	85.4280426
CD45RO	184.741454	70.3128809
pan-RAS	54.5448149	27.9184059
Phospho-MEK1 (S217/S221)	42.4182065	10.8557405
p44/42 MAPK ERK1/2	216.875903	99.3216134
Phospho-JNK (T183/Y185)	48.4345782	4.50744024
EGFR	148.007943	69.2634472
Phospho-c-RAF (S338)	14.2366253	4.85794887
Phospho-p90 RSK(T359/S363)	325.010473	133.083584
Bcl-2	261.877373	86.56764
NY-ESO-1	36.4549375	41.464935
MART1	62.5868451	25.6277331
S100B	32.508573	33.2785999

N103 N1 | N103 N1 003 | Geometric Segment	N103 N1 | N103 N1 004 | Geometric Segment	N103 N1 | N103 N1 005 | Geometric Segment
165.960046	133.346146	433.857257
403.646985	443.692787	491.722345
182.959625	228.496943	209.363379
258.543999	322.95064	415.439791
199.165423	174.573945	379.187717
151.032542	164.821569	488.650566
134.851731	155.249381	133.507293
1640.13314	1239.08213	2081.63575
2372.17667	2401.71954	5904.06394
204.619094	201.137508	190.261069
40.9710545	15.3624628	34.6316428
2251.02224	2041.3631	2520.2199
9810.82608	10147.9499	1698.46141
472.472264	478.359824	471.191671
837.762611	1087.51086	313.565331
162.292258	173.186356	223.215816
139.358545	102.794225	107.398326
48.8741845	35.3426374	40.9377804
94.3485532	77.3236485	168.969057
45.3254757	26.4860134	73.6669607
437.746545	340.376219	567.93752
339.782602	139.129937	259.584632
22.7022526	22.2944134	56.2000635
170.174914	140.108839	121.26666
165.486991	167.95712	575.428642
58.8241146	34.2258007	291.976806
65.8890675	40.8400253	72.0473346
60.8648835	22.5710423	48.7171366
64.8049004	32.1606743	37.4369485
51.0199613	51.2421165	91.1421751
133.339124	107.135776	129.019821
28.3451793	18.9790681	17.8495553
45.9236926	46.1236572	30.3636996
150.76552	135.038631	224.582805
28.6021438	22.9813484	24.0731414
117.203733	77.9855716	66.1606569
6.16526209	11.5586004	5.50012598
283.151607	243.47307	186.519178
101.59438	106.308056	493.167741
43.8529029	31.0068709	36.5782646
68.6625435	91.4647521	46.1660503
77.4292558	43.3606619	61.854125

N103 N1 | N103 N1 006 | Geometric Segment	N103 N1 | N103 N1 007 | Geometric Segment	N103 N1 | N103 N1 008 | Geometric Segment
311.092661	353.019051	261.014309
459.252474	478.819756	517.55039
198.501445	154.961807	187.108423
296.443151	290.047422	360.152611
248.03623	267.705364	299.917522
299.546764	351.285168	363.92323
138.956482	115.873731	111.834546
2072.23413	2381.74252	2180.76882
4238.62521	3534.89298	4862.88477
160.137872	199.09472	175.024516
28.4860999	51.8884594	29.4955657
3452.74926	2569.01335	1864.35569
5044.57575	4203.16604	1828.92638
339.708539	341.808566	529.965235
772.488359	572.592429	290.859322
161.15083	202.226569	243.274789
110.77556	102.897711	109.764624
18.4492927	22.157837	29.9771454
174.817898	182.822386	159.837845
86.9398237	85.4663008	66.0151963
764.163637	891.459525	600.130441
302.651639	305.591527	261.20984
53.1762787	31.1343788	33.0364163
179.284431	147.797639	178.212248
294.383386	285.929932	427.45056
218.475085	196.713045	166.540283
52.0021641	72.492719	62.3063248
52.7444189	49.9986636	49.1521771
64.3464648	77.7183682	46.7644144
85.6783776	98.4703773	116.807523
114.547258	97.3124715	150.700004
19.1410694	35.4887588	20.8103428
42.7358943	81.0223204	109.993683
144.617624	147.457244	194.359599
20.3802367	15.9100016	20.5748926
88.6846833	98.9034812	98.3865158
12.9020731	8.63323785	4.5803265
159.81302	126.422824	169.447768
120.291964	139.591373	680.674969
36.773348	34.4489046	62.4454439
46.4122683	39.2514676	62.4740822
46.6380094	58.2533609	88.8549377

N103 N1 | N103 N1 009 | Geometric Segment	N103 N1 | N103 N1 010 | Geometric Segment	N103 N2-2 | N103 N2-2 001 | Geometric Segmen
190.383336	249.941036	299.997201
290.484477	366.044612	1080.78727
166.557807	201.347279	243.42169
291.707212	384.540094	471.600638
174.490926	249.289672	465.707244
125.309127	284.812803	381.44031
109.781058	113.407912	219.786192
1652.09867	2001.03409	1970.80868
2347.3608	3322.00505	5927.16358
169.696151	181.2632	213.593953
22.0050195	36.2744724	41.0470709
2482.36343	2778.26783	8614.35464
5543.34162	8624.6646	13847.9401
335.08884	464.311234	462.283022
640.967319	1023.04184	1857.52538
151.423939	195.62171	308.784781
101.986736	152.133399	136.91827
21.2511444	32.5294699	57.7152049
105.839846	146.169708	208.112391
25.8305808	61.5055578	74.9346824
583.025623	627.348633	977.009177
257.807839	346.167212	388.987277
33.7706549	45.7077483	77.6794376
148.806608	208.211891	264.578076
153.773696	295.537538	729.100737
73.1143135	145.41929	127.932409
51.6481359	85.1400692	50.7398524
36.2667721	60.4487233	32.4085266
45.4500938	98.3272854	85.2847403
87.82249	105.466878	107.569245
72.0698624	126.130821	138.986305
12.7957024	34.73355	46.8346866
44.0774486	60.6397596	79.455595
134.162761	200.694056	379.027301
24.5233425	35.0357246	37.3042476
33.8772778	106.478672	243.92696
1.97142098	5.63301374	14.4028421
113.956425	267.001748	306.072145
60.854242	136.620837	295.28709
31.4659665	39.3351281	28.9663155
59.9842825	76.8323751	31.0892461
46.5580412	137.783148	191.817196

N103 N2-2 | N103 N2-2 002 | Geometric Segmen	N103 N2-2 | N103 N2-2 003 | Geometric Segmen	N103 N2-2 | N103 N2-2 004 | Geometric Segmen
313.593239	361.390539	278.270597
1449.59751	853.755544	1196.07807
326.112345	232.758896	259.851043
1018.71116	574.996636	409.826
764.663381	477.34362	456.026993
780.87848	573.116796	329.962654
230.301967	203.205153	217.206677
3305.81682	2593.51826	2427.6868
9683.7088	6609.1689	3484.20525
248.934013	172.086533	189.338128
39.5701086	57.0715795	60.1937051
4457.1593	4121.81394	4760.40847
4189.28882	7987.17166	7898.4045
649.374373	433.454728	472.12112
1077.26625	652.179456	1595.31804
413.022727	262.019005	226.409579
123.275225	137.865262	130.699382
78.8211901	21.6351786	56.4245219
327.48927	185.855015	417.388885
148.991639	103.4226	108.171375
639.011901	774.487897	1216.72735
389.141265	409.685867	500.473843
131.047636	63.532343	56.9567214
138.61847	152.886164	92.7806931
2794.48071	990.903997	488.887883
171.11916	233.462757	140.705394
99.0510989	51.355056	50.2252234
66.3900953	24.8495977	59.4070798
70.4708666	56.0511616	80.3997521
207.397342	85.9790613	93.2132599
165.248509	128.830435	135.87816
36.1195787	34.4745755	41.2085918
106.384182	83.0364196	71.2055047
617.259221	471.769236	404.728172
29.423422	31.2037595	29.8391025
221.478433	210.52166	262.04194
31.2403426	24.2957861	15.8408458
313.216485	242.627375	414.314951
1592.32819	515.093342	480.387545
75.3949213	35.5363907	101.946754
44.9558818	34.6734906	49.7356338
98.422606	39.8533843	129.303302

N103 N2-2 | N103 N2-2 005 | Geometric Segmen	N103 N2-2 | N103 N2-2 006 | Geometric Segmen	N103 N2-2 | N103 N2-2 007 | Geometric Segmen
272.076486	379.111552	319.892311
1049.51018	1175.80005	1507.48554
234.918148	262.466294	349.922581
769.781071	835.986298	861.384942
626.314321	801.628721	615.180955
727.958369	938.351866	594.450596
202.886218	188.13517	235.629265
4326.32883	3989.8037	7362.30422
7830.24311	10385.7731	10170.1402
276.894262	220.756625	273.726847
42.2540353	44.5863147	75.910193
3807.58048	5669.77973	6043.20355
1853.6338	6897.43364	3301.8978
528.987356	427.045658	623.743214
1085.58021	949.559675	1221.97111
342.697569	398.298721	438.484494
129.642135	155.213048	221.813911
55.6990179	71.8341736	63.5329299
165.39956	314.775616	351.720893
108.475441	109.69363	145.385347
655.663514	872.470646	865.688531
368.315644	391.136735	481.520109
81.2129853	88.9916537	115.437917
170.223647	140.571716	146.890142
2662.81093	2241.20699	2491.92166
172.848617	188.903177	232.366311
93.0379423	59.4206944	67.0253496
67.7655059	57.7547912	57.0805188
11.7579223	99.2553581	104.610803
144.706923	106.22209	146.400644
172.880013	157.260692	224.394431
54.2383114	15.8978006	37.1200675
62.4799534	71.9221471	73.8591336
497.185666	445.934334	670.814192
19.6369551	6.90694946	35.8381411
246.354873	228.180814	258.523681
20.8495533	29.3338372	33.294779
327.190166	254.142327	435.409979
1329.868	974.146553	1450.54542
178.908316	106.190689	183.664146
49.0961353	34.5373836	93.335617
191.08787	119.720943	212.514751

N103 N2-2 | N103 N2-2 008 | Geometric Segmen	N103 N2-2 | N103 N2-2 009 | Geometric Segmen	N103 N2-2 | N103 N2-2 010 | Geometric Segmen
218.207598	281.283257	387.689111
882.862222	1165.12044	966.487188
185.551663	327.407217	215.981911
521.917985	586.227418	571.530879
355.450058	397.73909	670.445818
457.372651	371.358502	722.39664
194.243671	219.777284	201.336132
2281.85874	3375.26263	2476.30863
4374.81498	5372.77221	8441.76001
149.692637	248.095519	265.61945
31.7058994	48.2999443	70.758912
2350.55396	4349.78416	6410.38214
2005.82383	10127.7255	5508.19585
364.463504	402.676729	430.076078
764.185964	1189.45301	1183.59733
251.06732	295.218599	332.686272
97.6726087	140.937799	165.762698
43.0165901	36.0146177	42.9904749
184.765033	223.441303	375.167722
89.9640515	77.0282065	91.9481974
566.4601	617.430791	1272.51826
374.461831	533.875772	433.447468
47.3698093	23.3706277	102.290486
128.606529	169.200051	201.973282
1062.77614	930.705034	1682.0416
76.0642796	115.469195	309.363042
47.9597004	56.9915242	75.3194895
26.3507868	21.6676134	49.1425979
89.1559025	92.8602681	110.846853
61.6213826	117.684626	99.896582
124.307976	106.551849	136.064177
39.9844584	35.0701345	38.872832
78.960331	75.551612	62.0025956
509.395013	451.707951	299.556442
49.6332148	32.6497517	19.4869253
228.313109	273.071034	209.159959
15.3702812	14.4441254	17.2418825
284.276663	290.893631	252.53696
1236.1017	686.04256	453.64948
109.516741	185.915792	155.348747
34.4701414	31.7452373	54.1344803
107.539068	64.5360283	168.887371

N114 N1-2 | N114 N1-2 001 | Geometric Segmen	N114 N1-2 | N114 N1-2 002 | Geometric Segmen	N114 N1-2 | N114 N1-2 003 | Geometric Segmen
434.228109	95.3024312	97.5695709
344.776796	424.985181	541.624206
132.122953	172.602716	170.628696
379.072111	352.596102	673.579602
550.974426	304.721539	754.117092
727.439324	405.645566	1276.19103
110.31411	110.750335	146.405904
2570.81907	2018.23962	1683.82075
6409.11997	3814.65885	6848.47406
126.517324	150.434557	164.575129
63.294836	38.7720143	39.4397531
4389.84313	919.079904	1294.24284
1630.62883	419.794626	171.280556
239.680577	270.077775	333.820662
377.988356	206.877175	284.084648
257.88259	193.367595	230.027323
104.591597	84.9721702	90.558628
23.3116834	45.8336739	62.6566678
432.476909	29.6794759	70.9182103
107.70708	42.9645522	151.784859
2334.85578	67.219812	238.729504
734.676639	153.673155	387.207934
80.0366754	26.473069	52.7056888
151.245727	159.492864	121.217968
1497.4964	608.911724	1735.67194
416.409986	57.5442944	104.696052
78.1639285	41.0595634	104.415539
36.9469879	37.3936079	33.8715962
425.048716	18.5417952	56.2021919
100.600382	73.0087236	142.130053
110.046399	128.402958	181.029734
45.4090523	13.0777821	39.737055
30.3529505	62.0971062	47.4968426
354.554513	495.499071	490.142881
28.3651222	32.3936122	39.0061012
266.506253	72.7186405	165.955121
30.2289701	17.656185	30.3774621
289.127757	329.818049	322.683388
313.739671	1259.55496	1592.78918
36.2830842	57.234246	43.4564406
45.1548143	50.5861689	54.7021478
197.449932	97.9694442	499.249955

N114 N1-2 | N114 N1-2 004 | Geometric Segmen	N114 N1-2 | N114 N1-2 005 | Geometric Segmen	N114 N1-2 | N114 N1-2 006 | Geometric Segmen
85.280756	117.105193	489.225163
525.342999	494.618139	435.109641
138.492296	149.327791	133.937588
438.038395	308.113802	436.868927
423.225331	280.270975	719.267961
512.940279	240.793338	725.329404
104.875624	98.4453507	99.6927088
3493.96773	1361.34079	2431.66396
4946.96719	3992.86508	6813.78869
179.211395	184.828621	131.173222
40.4296754	53.8656481	75.3851704
899.247256	1272.35892	5781.88125
143.615134	507.512726	2016.73913
294.181183	299.226391	255.658888
186.285035	194.305259	470.562447
201.431279	186.110174	303.584691
64.9681281	95.9033841	110.005416
33.3211171	27.5448973	38.0092795
97.6831362	45.918722	350.237167
74.6442248	34.1579253	94.4592888
247.620689	172.733174	1238.22598
219.284442	106.774657	583.850538
49.628063	34.8852314	126.362845
118.659547	163.100301	203.86388
1320.38397	763.283392	1303.40535
70.0329048	59.6237029	394.786873
56.1819643	31.7912119	93.4595759
43.0312411	33.0309294	65.9587525
43.9113285	18.9717566	267.245786
98.2274853	83.5879662	121.522368
150.927173	89.1170921	147.567504
17.3084065	28.9566507	61.6988675
28.5248656	44.0506926	32.8510799
531.907147	471.398461	315.428678
28.6313342	36.4124444	20.412288
94.8749109	99.5915346	131.521581
37.7797465	47.4935738	33.0165008
345.890474	334.557923	278.694339
1339.24313	1191.96374	355.37494
28.2174447	37.6692314	47.2317141
51.8393974	45.2485426	41.7787612
245.357863	61.1037558	185.633924

N114 N1-2 | N114 N1-2 007 | Geometric Segmen	N114 N1-2 | N114 N1-2 008 | Geometric Segmen	N114 N1-2 | N114 N1-2 009 | Geometric Segmen
730.399852	973.040707	772.1949
1205.07644	2424.41653	2318.87578
397.313282	525.612935	436.329347
876.942904	1422.59564	1848.85353
1457.4302	3217.88446	2773.40814
1118.32718	2677.5027	2366.07625
306.904869	397.468288	317.498621
5701.71523	13939.8564	15576.5484
6851.69582	13273.6655	9751.93519
446.87477	539.941085	495.3279
95.4451241	176.48894	143.885478
8885.82506	15526.0377	12424.412
5102.4881	9030.73781	4730.35975
576.632638	646.402592	642.529397
652.966807	1080.08333	894.873479
672.644726	915.886847	848.470479
276.49392	370.784477	285.45363
155.524216	104.861797	128.135322
689.44312	892.43195	543.625828
246.98022	353.802967	311.149098
1312.23548	2308.3805	1206.18852
151.501112	212.318803	355.934068
235.715551	307.239095	264.113324
281.706367	296.550213	301.551777
2347.96168	3829.90642	4838.61618
556.071387	1109.1123	684.037952
190.832118	311.582667	316.473633
103.174114	121.544665	139.619786
349.235597	1001.19534	472.98721
313.097369	359.94749	921.760232
345.328748	710.784848	619.89863
71.9326485	89.2945583	101.398385
44.1922807	40.7521627	45.4850511
324.372382	351.511633	287.107255
71.8164638	49.4259923	61.5347618
247.697614	387.491024	394.291636
72.4662689	90.5373428	85.6746906
212.728797	224.331955	245.437165
642.704783	856.313129	878.01343
72.9505216	85.4459575	92.8816086
60.5253077	137.790211	113.757681
784.378339	736.235351	716.061287

N114 N1-2 | N114 N1-2 010 | Geometric Segmen	N114 N1-2 | N114 N1-2 011 | Geometric Segmen	N114 N1-2 | N114 N1-2 012 | Geometric Segmen
317.746072	281.103409	444.295941
3099.53481	433.820681	1743.07782
218.207513	165.76748	332.042874
515.890882	756.831607	2348.98994
733.207212	763.731313	3615.90763
731.215172	851.034538	3613.89316
128.223692	128.270912	234.139125
2530.1796	1637.87321	15243.7056
4948.71398	6623.26987	9205.14329
170.904676	146.543668	409.599077
116.655704	66.4023871	109.065261
2629.71635	5373.16794	6257.91846
1977.59564	1774.88661	1290.7045
376.790955	317.522311	757.594355
555.495797	708.801484	770.159869
252.808483	294.435475	622.856932
116.168919	99.6100282	268.841332
25.4213063	49.9530976	181.327473
347.297709	214.555991	975.412307
161.944401	105.544232	499.287903
1219.15737	863.299211	2644.87393
350.846341	431.991689	490.141545
69.86474	83.7397316	240.059479
211.478404	221.365283	248.762417
1303.20953	1675.45306	6340.66551
335.487049	266.357615	667.674709
87.0639984	122.827778	267.515548
45.1403457	67.4422264	148.30653
183.845731	188.292325	542.025509
349.901781	98.433812	306.572205
203.560222	246.830527	553.412085
41.2653392	45.2120151	68.0255389
100.44072	25.4184677	49.7522894
504.439585	324.268015	543.952782
147.022769	31.516481	91.8858996
221.16677	147.259365	468.487231
34.8663852	51.4883798	68.5301751
557.093388	329.231148	450.152109
901.413751	400.274742	1112.41582
42.119254	71.8966224	124.62371
66.631889	47.6117334	124.69664
127.33886	191.07863	1077.84285

N114 N1-2 | N114 N1-2 013 | Geometric Segmen	N114 N1-2 | N114 N1-2 014 | Geometric Segmen	N114 N1-2 | N114 N1-2 015 | Geometric Segmen
422.187678	183.362439	199.141988
602.838428	891.841354	571.016438
223.32447	251.709095	297.568176
356.354856	537.311314	1238.01805
432.935978	492.437745	1157.22472
525.452746	507.301386	1681.02685
181.543553	224.240763	178.525337
1858.97813	2270.278	5528.19855
5010.80095	4907.37672	7939.7132
235.824375	308.948482	359.062694
69.8270972	36.0440219	49.4556502
3104.24725	1810.82026	2671.54287
4539.19897	735.330023	733.566751
374.083053	478.405496	476.834022
624.309009	531.904764	687.839708
255.450664	286.268213	417.468052
178.639442	123.370982	123.726941
21.9453008	32.1235838	73.0086926
243.939584	138.605947	205.023172
57.1977555	69.4261674	88.2693141
1138.52189	348.8172	432.702236
575.496519	616.813529	451.908955
55.5960064	44.8305407	48.9875741
210.904431	191.945656	190.442711
272.493343	871.062058	1666.42722
248.151962	82.4516753	122.516832
93.5396939	95.7911048	180.681237
48.1313203	50.3497906	82.6454783
224.583562	54.0946522	103.95207
134.51618	156.840047	206.662257
200.325343	207.077631	242.276512
32.8091027	35.4653857	49.6349432
78.3065833	69.8114826	66.718761
391.286078	639.124455	619.479927
83.0909979	74.8484933	59.3371341
139.924891	160.076576	205.1506
38.9050443	53.5243741	47.5613181
412.730164	579.014004	435.977048
166.601351	1603.23749	1515.96651
54.9580571	56.3347538	45.1285848
60.1635276	64.7589032	67.1349328
75.3605168	227.33536	370.699312

N114 N1-2 | N114 N1-2 016 | Geometric Segmen	N114 N2-2 | N114 N2-2 001 | Geometric Segmen	N114 N2-2 | N114 N2-2 002 | Geometric Segmen
1410.16722	256.678966	212.323269
19791.3461	784.353166	575.029394
1989.41712	252.914483	187.346919
10096.6729	496.983996	460.297838
9119.38573	490.777873	530.243262
10960.406	471.871852	485.238081
1263.69761	166.713863	109.60762
10908.9029	3199.47079	1925.9361
30360.4805	5172.62579	5280.17734
2308.14561	273.852691	238.689925
504.624445	68.5071209	31.2635172
8707.94943	1151.81673	1305.65994
68826.1919	2907.93703	503.308946
2592.53226	403.917591	425.67812
6534.63852	441.244334	359.045124
2177.18021	307.193003	321.021186
1402.02655	121.32062	108.232688
1848.3714	36.6334408	42.1893883
1997.14107	271.152075	125.932886
690.343024	58.7572952	42.6969175
2673.56298	874.906811	358.627541
672.72703	531.973571	249.581433
988.711113	30.3982551	50.7768696
1991.9099	245.30348	237.609578
13127.92	481.881005	744.083393
1436.74361	110.743071	170.635004
1967.74068	101.147499	101.185591
579.556416	57.4184402	60.2404298
1222.42692	213.654745	81.9144225
1806.62126	168.272286	225.236249
3545.52204	197.915716	183.664426
286.387608	47.1851346	11.0094238
139.079806	38.1475966	45.7130878
430.636532	505.653196	691.583079
374.248502	69.4567894	42.7114253
1274.96116	185.903796	144.772372
154.362961	36.3325632	44.5056073
2162.95693	384.597111	418.609079
3873.63798	921.115745	1593.62642
326.49077	36.9887609	55.6431441
351.357163	55.5187858	66.2969008
1197.67409	143.128246	112.105135

N114 N2-2 | N114 N2-2 003 | Geometric Segmen	N114 N2-2 | N114 N2-2 004 | Geometric Segmen	N114 N2-2 | N114 N2-2 005 | Geometric Segmen
214.708235	284.497914	287.829306
542.979358	620.877412	576.184929
234.961877	282.971574	265.300278
402.538548	460.62178	356.126908
361.846429	544.760151	482.989827
408.550803	634.306624	524.187562
163.877831	197.797168	152.391068
2575.92746	1805.32097	1839.8801
4621.07882	4273.79206	4115.25639
230.835914	281.377623	250.309729
30.3380544	57.5693174	33.4198522
1196.54792	1831.94689	2184.31304
2952.97402	3820.36184	2449.51341
390.272534	407.392609	375.301415
472.159063	466.370528	466.972969
225.604361	328.765217	266.868611
139.006044	139.818029	137.637454
26.7147087	24.1540463	28.6399286
213.915362	150.538857	135.114487
39.9861364	59.7535015	43.8559271
530.873376	608.74671	463.586581
361.902418	382.029744	383.363783
36.2152089	43.4263112	35.0246712
284.573965	225.097203	167.614861
433.730479	762.210739	596.016179
116.230675	140.295207	167.985246
60.9180603	75.1385443	87.5965962
49.3638513	61.5200867	41.4397005
120.824179	127.534367	89.5396493
151.011174	151.23891	161.61993
176.841376	215.266623	216.085805
28.1926295	30.8149467	27.8036749
48.5585613	87.668859	81.5178928
448.581177	575.635068	497.961727
53.8996269	86.074279	71.7730095
184.362583	156.061459	113.859888
29.1759961	45.6754668	40.2123309
390.509318	517.984252	410.427044
690.191063	1055.49219	839.675818
51.2961957	58.4036194	50.7761974
58.7080038	58.3623783	51.1502832
155.868011	189.038319	237.166376

N114 N2-2 | N114 N2-2 006 | Geometric Segmen	N114 N2-2 | N114 N2-2 007 | Geometric Segmen	N114 N2-2 | N114 N2-2 008 | Geometric Segmen
407.959078	222.454632	308.95053
413.842903	832.908001	817.167435
213.97696	138.593213	103.37892
358.821083	340.942838	333.429041
424.494555	263.203045	235.503855
479.838344	502.236848	554.641967
134.005658	149.592389	163.978475
1755.73722	3139.49077	2068.87343
4566.8881	8893.83368	9420.51933
245.778525	96.4416131	112.979789
52.6811748	29.6266295	27.4308003
2301.4258	1654.35706	1690.35159
3799.49724	284.000104	163.551936
328.294177	470.141812	412.473987
499.779347	394.32512	300.056175
228.424472	263.486884	275.920519
147.33355	128.891679	118.140125
29.0380837	93.4559891	59.9888253
194.379506	238.273701	213.198047
34.9874193	92.6212681	84.3151962
623.410078	678.007378	558.066749
332.165099	546.898402	363.46731
44.2005931	66.8827868	54.6402818
180.006579	207.627677	149.186176
229.60015	2304.29425	2395.7472
231.930976	105.529544	107.287257
66.8745846	197.089231	187.848726
45.3940967	64.3230681	64.6758211
178.517299	101.961762	54.941402
116.517433	88.5796572	139.562356
172.50734	127.596481	109.82501
31.9185863	41.1068738	40.5788538
59.0955813	34.6896145	31.6739862
352.925506	275.640923	297.729744
60.2263029	7.36672683	6.01829034
130.186075	127.408171	99.2828385
36.4937151	18.3909551	9.3903736
313.610631	85.5428482	75.2604758
164.626739	1032.61995	1142.18412
52.7047928	38.4101001	27.1954407
38.6131544	40.7120716	48.0421699
73.3195031	62.4674495	80.9491286

N114 N2-2 | N114 N2-2 009 | Geometric Segmen	N114 N2-2 | N114 N2-2 010 | Geometric Segmen	N114 N3-2 | N114 N3-2 001 | Geometric Segmen
190.397539	271.230059	484.93079
700.111336	754.233215	639.64838
103.777713	152.126276	201.383189
351.451035	398.563176	388.822785
287.806316	352.015463	322.530973
515.10433	709.457495	696.604758
136.968805	159.847671	140.806113
2219.60781	3093.73493	1986.17221
8663.23165	9019.15801	10838.9245
100.283283	113.411166	147.471951
29.4803782	28.0481112	46.2059724
1208.74117	1851.69724	2254.20508
112.255252	413.219982	157.540704
452.957088	398.325511	467.951344
253.536903	455.089157	355.817492
239.267812	274.015068	300.197222
108.283177	126.488583	154.968036
107.301514	71.9750263	83.6603075
151.985556	198.425432	303.358305
66.8442522	60.5453513	72.9635091
349.231384	514.793288	574.997084
381.598606	654.829735	653.815739
74.5329127	55.8699229	73.9600255
126.374235	179.592697	131.67302
2403.4847	2281.00947	2473.13151
131.933881	92.5063784	193.975034
182.771111	131.568631	128.779645
59.6501318	50.7465917	107.4845
42.3435343	71.8834465	85.18957
101.702752	131.447377	102.202564
115.107085	115.045801	135.163091
25.564969	44.5920333	51.5175028
35.1431366	32.1973905	39.2127664
288.865081	253.949173	281.305297
2.81936965	7.74913862	12.3098754
81.018675	128.351854	129.377904
18.4761316	10.8819237	9.60358525
83.1058793	81.3311822	73.3040864
1480.8934	945.869169	1014.63068
32.3404139	10.1009986	27.8129226
49.3404388	37.47228	45.3535235
79.1415342	158.044306	151.176455

N114 N3-2 | N114 N3-2 002 | Geometric Segmen	N114 N3-2 | N114 N3-2 003 | Geometric Segmen	N114 N3-2 | N114 N3-2 004 | Geometric Segmen
64.2649293	93.9033425	129.349797
813.641441	851.972582	867.233123
130.181519	92.12851	112.059513
319.345032	259.311996	245.319924
300.688805	212.211431	311.649706
204.927826	204.526967	402.648441
85.9863404	108.850477	215.341175
1854.84004	2182.06509	2032.83038
2886.86598	3879.74158	3724.21569
88.3604134	113.615455	110.347737
19.7886554	18.2977524	24.3405902
657.26538	698.791705	1541.5411
101.273876	135.007662	1952.64636
302.615034	862.711913	336.392303
159.855586	212.812438	367.779984
170.713	162.95674	184.678615
54.2166521	48.7881817	109.831602
53.6566879	48.9813206	44.5520787
51.0445847	107.722238	175.979431
50.2839106	28.5395979	35.4616337
217.395947	127.22616	307.71388
288.983138	197.080693	285.857582
35.4631211	43.289261	59.0621038
54.6464294	72.9285843	107.154696
969.344843	976.773973	864.600718
42.3244514	36.8535706	79.3918581
59.6885133	64.0419606	58.75299
32.8331376	20.4780588	35.622772
8.58472749	26.7001734	62.4490776
52.3756069	85.3396799	63.5094447
43.076868	68.365467	104.120994
11.2068791	47.4790304	37.7874318
33.4594425	42.1072554	29.3775461
456.87907	479.78375	410.21437
13.9769916	14.235439	7.28334486
109.994446	111.459508	122.34788
15.2358617	9.81842486	10.0468941
110.898186	159.928257	166.989488
685.582049	716.091261	443.151144
14.7990391	24.4127883	18.1678984
20.8665376	31.182369	29.4535064
98.2838371	89.1106125	70.9210185

N114 N3-2 | N114 N3-2 005 | Geometric Segmen	N114 N3-2 | N114 N3-2 006 | Geometric Segmen	N114 N3-2 | N114 N3-2 007 | Geometric Segmen
152.10625	119.194855	102.527311
2061.22881	615.269027	1093.08572
128.113703	111.793607	115.668193
227.043619	242.89767	267.63796
278.142431	282.669622	182.537819
205.459723	218.225721	205.96091
157.053515	104.393437	104.985709
1908.30976	1578.86076	1168.6738
4023.24413	3849.0853	3416.31904
72.0895367	78.2189234	94.9382306
17.1247638	55.742372	19.7154714
743.176404	1049.55407	723.744328
499.754058	900.332095	139.016864
309.002219	231.556849	311.348686
234.209127	165.413184	157.891427
192.687743	192.760449	179.152677
79.9060246	90.8285772	72.0215251
36.0788348	24.5624626	42.203882
106.122777	134.336799	82.1109393
38.1572136	41.4015169	29.2238022
270.614203	446.959574	97.9820754
241.750549	207.253572	143.4625
31.6517004	45.7904996	39.2577429
76.5663733	104.438905	50.6458899
831.90182	642.3238	976.033977
56.7287006	65.655507	26.6578828
46.5024659	37.3750527	82.282447
35.9349381	20.794823	36.3463461
17.8489589	38.7331203	14.2549646
65.8011051	78.7815948	93.4120563
66.9588038	48.4346346	66.9144717
30.8578876	26.7852547	22.330866
13.4345264	22.67501	39.0974261
478.962047	433.071352	436.413475
14.5718863	15.0579578	9.28353387
99.5727677	99.4643899	102.582052
11.4862609	9.97030088	16.8661281
139.366645	143.620907	115.091723
555.525359	482.206961	670.602407
7.78901986	30.0489901	15.3586543
47.7036414	28.4170497	17.3244728
48.6489046	43.5687862	30.9874544

N114 N3-2 | N114 N3-2 008 | Geometric Segmen	N114 N3-2 | N114 N3-2 009 | Geometric Segmen	N114 N3-2 | N114 N3-2 010 | Geometric Segmen
206.694713	67.5363595	101.89467
1225.68558	768.355033	577.290716
103.569189	87.2720624	92.3404725
212.242511	209.311152	255.622943
240.308107	172.218166	244.261708
254.457005	153.884619	287.136373
100.980202	102.913994	146.896005
1802.61265	1415.58286	1276.63927
3904.62414	2765.73241	3541.0307
89.0709768	84.2604302	76.9558127
43.0346035	13.0937805	22.5374898
924.909355	601.726885	541.291009
890.966798	113.918727	172.243741
296.457077	236.748048	329.783866
213.800219	127.201668	204.033951
134.621448	169.436364	144.653447
63.1098839	37.0699419	72.7252647
28.444318	26.6277147	40.2040248
148.159437	75.9942901	65.4020642
44.7483112	37.4309059	38.6564597
445.813591	185.973366	124.976188
171.005671	143.411024	173.045457
36.7655513	66.4849576	29.9179923
129.007409	68.1125475	73.8163845
845.624941	835.436939	1080.50688
85.1555193	21.7282439	44.8793298
58.8632643	63.7132526	55.1534402
27.2920721	32.5876029	31.8541278
29.9030058	21.3013217	16.2953811
66.9036503	82.7892196	39.7673881
74.7856691	57.9257606	87.0431048
39.289974	13.3476854	7.65817343
19.5063679	29.5193104	32.4617577
357.746665	433.746653	422.888239
11.1601407	12.4852213	12.7348241
98.5019722	79.2617753	118.409418
6.15787136	13.4417102	17.3522694
144.569399	137.586076	122.794594
436.308273	725.910592	581.660492
12.179278	16.5244141	6.32053797
26.3264678	19.2311714	18.6725788
40.3635232	59.2701844	70.8458271

N114 N3-2 | N114 N3-2 011 | Geometric Segmen	N114 N3-2 | N114 N3-2 012 | Geometric Segmen	N115 | N115002 | Geometric Segment
184.909689	208.724143	544.447154
675.983237	258.329469	236.530409
125.780913	196.285282	203.625359
251.608103	507.325713	358.969213
278.996692	417.427944	641.219871
245.849011	477.311908	1078.30608
146.134175	130.395168	141.137197
2217.36009	1041.79901	1094.87431
4438.08837	7978.2096	11284.9362
123.567413	196.353545	191.533642
32.4759449	32.2161196	52.3845759
1701.5851	3866.92223	4273.55758
2024.79996	5655.07772	4178.61093
355.427646	235.411307	235.293778
271.178017	1355.07499	732.230851
141.143891	285.945717	311.504008
89.9226501	186.324986	209.05067
29.7213593	47.5745584	33.4963041
134.169732	247.819106	248.07624
47.3139745	37.4135294	51.8731286
410.561081	413.236079	772.074564
295.19296	133.191143	214.192258
51.7140941	119.878658	193.078133
127.650899	155.401644	188.076771
496.337746	502.027223	393.046283
121.816046	209.524611	911.509451
54.2700001	80.6333608	129.869144
50.3247918	43.591535	48.3339167
46.8683049	135.253969	195.803039
66.2001599	58.9702954	97.8667569
84.6551985	138.319112	183.728525
28.8097852	47.9403616	43.6345339
36.583309	37.9391258	44.7631495
346.442109	307.794742	271.685182
17.0058998	36.4412089	30.4994878
132.805593	135.646453	137.181317
10.7238938	25.1703708	24.8491024
142.593523	225.002453	229.784744
181.528893	93.7490808	76.2290401
15.1500966	76.6844099	73.8296067
26.1985007	63.6858163	60.6178191
64.8856789	82.6580783	58.2271056

N115 | N115003 | Geometric Segment	N115 | N115005 | Geometric Segment	N115 | N115006 | Geometric Segment
305.151543	454.628358	426.194497
308.500421	268.785746	254.584887
241.366818	296.573534	198.659425
540.109727	705.900146	374.930933
522.351864	761.006678	706.32386
601.114426	1232.4456	1229.52657
160.460878	166.852756	193.997959
1337.35598	1570.35664	1130.59865
8749.00529	10394.3513	9534.09835
250.250571	378.020676	226.779673
33.6571449	51.9347673	104.318586
3579.00623	3198.9396	4013.79605
6338.72703	3425.21858	5737.63781
236.580011	402.309598	272.498078
1078.11821	901.581174	692.907102
322.20613	339.909874	308.368266
195.658931	172.718483	187.039416
44.9210531	69.9984898	41.4376458
240.074656	300.593041	288.825701
56.7048562	84.683603	52.595275
514.435719	634.506395	698.083826
188.255228	224.909547	363.920812
133.951657	116.882788	177.486793
201.138364	222.089503	239.571844
423.500118	917.160832	822.658662
449.989422	610.116534	687.46663
82.7424851	126.998735	115.076441
50.8786524	49.8799507	48.1848161
236.780166	216.054796	170.072342
111.813554	105.739268	64.738348
168.662365	384.334614	227.287091
52.118475	44.5489991	75.7489522
47.6434419	58.7587724	99.9105599
268.750763	450.655218	374.660462
38.954379	73.0307528	63.4079339
135.150062	181.936439	173.28468
27.8671843	37.3783514	27.4913751
260.815461	369.097321	314.463807
101.772475	490.826977	200.980087
56.4588726	233.92492	177.293885
69.4075591	168.602757	137.480127
120.94083	1290.4743	562.227263

N115 | N115 001 | Geometric Segment	N115 | N115 004 | Geometric Segment	N115 | N115 007 | Geometric Segment
234.10543	366.217294	368.310658
369.8708	366.833867	334.365044
338.472685	230.975705	283.06092
1007.3322	464.528967	1506.65528
914.192902	582.103502	1172.06072
1410.93537	870.991339	1878.68992
229.705925	152.349194	187.222688
2844.78313	1616.42138	3107.39997
14174.3185	9091.10034	12289.0777
502.56433	210.701712	582.111159
37.8895223	40.9613912	77.7142802
3627.94159	4411.22979	3530.90532
1135.79783	5310.217	1738.97474
559.752643	308.362852	381.399684
807.425776	665.289882	1022.69521
434.30163	306.999276	426.410042
200.011895	196.628892	159.076085
109.568039	43.1175378	159.961836
201.416248	283.069724	432.367616
109.792634	64.4012785	129.919043
445.570471	680.393925	551.899917
398.721486	176.981213	415.144723
130.620732	143.09582	124.143278
208.835561	224.562223	265.336594
1527.95468	505.152098	1501.23599
195.968801	400.110078	462.7155
119.419656	153.308065	178.693959
68.2546507	51.7937479	88.1090299
155.116165	279.177682	382.016366
127.981143	111.66619	176.158074
419.643785	161.758008	340.568518
44.730413	49.652177	68.5112241
140.471552	45.5580582	121.795249
674.778413	362.007251	469.938255
134.413652	60.2633873	75.5402359
242.886074	154.832821	204.04003
55.1179399	41.2896221	40.2157955
435.495715	307.69502	326.152264
1102.03307	230.384322	603.287767
159.453935	110.224748	332.769793
191.934256	109.441068	254.009898
1114.68198	305.730113	2894.53127

N115 | N115 008 | Geometric Segment	N115 | N115 009 | Geometric Segment	N115 | N115 010 | Geometric Segment
162.472865	275.793011	211.806987
359.279396	367.488638	501.387474
231.246551	200.329916	215.902958
727.857251	597.204557	931.421307
551.317273	435.55731	797.646641
606.288778	492.197877	1103.13271
163.598411	147.171662	123.556911
2761.6981	1336.2736	2209.94052
6576.55853	4110.36683	7730.12614
466.371794	1545.49507	1426.07222
45.0331974	43.2270025	35.0423516
1992.68565	1332.34183	2242.62568
2776.65296	2093.5405	3518.94766
314.603767	260.576216	296.578731
830.960399	626.450841	642.645792
314.384999	324.804896	380.701885
170.568785	114.800848	134.660027
117.812387	109.892607	134.9644
534.454738	1192.25484	931.032551
215.098174	101.647262	132.563013
537.319488	431.249198	541.131094
412.678302	246.472815	389.21671
72.5588571	50.081813	116.817617
211.182075	212.327793	247.317024
1925.80523	3201.18527	2529.13406
149.547109	243.016129	357.757762
102.985387	70.2171595	133.860532
65.3050262	47.9197865	55.9391017
170.740413	395.134634	341.257333
116.98677	121.158941	93.9457004
190.530225	127.991008	213.77866
52.104122	47.6668478	62.6447739
39.6035166	45.6123811	59.241676
493.510296	491.509494	479.287953
36.4581058	28.6632605	61.3433711
120.27333	124.277573	136.209556
36.7623548	32.5978891	37.234758
439.941929	369.577292	382.734505
242.123482	254.596318	288.518194
1092.35204	2821.31481	1613.116
696.398022	3316.21309	1782.04274
3522.39429	7184.73293	5483.48405

N115 | N115 011 | Geometric Segment	N115 | N115 012 | Geometric Segment	N204 | N204 001 | Geometric Segment
162.788025	380.869705	532.753056
335.831563	625.202213	478.347274
214.878536	225.525365	228.250336
381.443959	385.445363	335.992303
432.443581	480.06676	448.803512
318.77673	391.422801	475.20536
156.890794	152.360198	172.446536
1530.46198	2929.02038	2266.21053
11104.1009	5062.2573	5268.14621
244.43339	259.366402	246.022155
16.089499	85.3203653	48.7507832
2829.52213	4669.90368	4056.59336
1687.53452	3510.43966	5435.8842
273.490044	421.335636	359.554061
657.34847	879.78046	871.188793
427.770762	268.900321	234.251386
215.790341	198.578486	234.35132
62.6883963	49.0193998	21.3680099
150.606973	423.079458	302.734835
239.907083	75.6803454	74.4605537
1161.33835	1343.55451	1120.16792
569.843108	219.415144	292.49906
178.412557	104.298418	79.9840983
179.147657	180.982015	189.194067
2020.38894	596.31172	583.659799
85.7336191	184.554763	178.713113
66.1443305	65.6177761	71.7226201
63.3960501	39.7789708	37.5596878
400.687727	221.007388	254.883194
145.121722	146.457294	96.7506698
156.225394	204.458181	195.108024
47.1911342	36.1295958	39.0536339
81.6948144	146.567017	139.146496
548.660943	598.103997	474.974472
36.1809326	64.3624082	63.5558118
137.857687	207.472616	176.513834
41.8111877	52.332058	36.4710821
320.801036	560.582821	517.167853
185.20552	278.782858	145.900528
188.186501	48.2916176	55.0721979
137.423599	67.2109578	64.2383109
1148.94786	181.172387	110.603178

N204 | N204 002 | Geometric Segment	N204 | N204 003 | Geometric Segment	N204 | N204 004 | Geometric Segment
501.728835	561.839994	472.753545
548.093309	571.411503	883.906397
262.501762	287.627504	356.051768
493.583908	521.584385	509.93167
561.569593	642.30988	605.188577
487.03268	772.937913	505.828422
153.990825	162.053713	200.280773
2068.25509	3142.22881	2970.52203
4659.08741	8003.64758	6359.53158
288.739088	263.516616	353.039885
72.4560206	55.8134681	70.6401149
4305.15189	6027.95397	6340.8418
4313.81932	4745.10434	4008.64019
533.762753	435.547986	532.770675
741.710884	609.303236	1110.13654
297.781838	289.589188	314.670905
216.410082	240.554801	250.413396
29.4543203	39.0249166	39.3371656
240.490841	329.890101	397.77661
66.8632102	96.9884047	80.7802609
1088.41324	1191.07897	1365.30416
276.435759	243.70441	356.505884
82.1959812	104.068874	80.6349495
152.79232	169.483939	200.793977
519.438875	1195.63533	554.371615
197.61463	353.739984	266.434045
84.0667562	103.968983	78.0496354
46.7934787	63.6705967	63.5940508
243.949874	181.329825	280.526186
137.288641	143.458371	167.409509
202.776404	220.716333	202.289099
47.964611	53.204449	46.121931
156.992699	155.433585	156.367766
638.482994	595.92448	649.718395
118.55287	49.5074032	116.233628
171.942946	118.674362	155.784506
50.6777919	55.3993321	60.0307921
475.212311	497.065307	533.665703
578.593662	311.96425	226.302273
62.2288878	59.614236	59.8382101
101.025068	68.960882	47.8247079
60.5341089	166.268978	58.2085404

N204 | N204 005 | Geometric Segment	N204 | N204 006 | Geometric Segment	N204 | N204 007 | Geometric Segment
610.186826	546.250961	294.778785
923.795694	1059.73307	854.808463
299.009747	337.625439	270.226787
508.38074	507.537022	379.319467
546.331588	537.76765	417.082928
513.731874	506.148401	392.643299
177.387356	190.016585	187.26863
3476.99993	2691.10945	2505.50415
9321.55074	6348.23072	5322.32178
269.790122	344.882023	252.497588
74.5837112	72.3647975	42.9117627
6037.44595	5042.0046	2907.89602
2384.36643	3805.83741	3737.98908
463.81006	518.972155	534.730477
1126.2749	944.230612	715.624646
310.150068	327.931094	242.969546
259.786968	238.912061	201.357393
37.3799026	34.3955385	30.6163387
433.651208	303.288552	263.069944
93.1226971	69.22891	74.5986407
1722.82337	1407.68564	1250.16724
369.070994	294.227061	214.46452
114.639279	90.9332256	59.2471597
205.970996	191.571633	161.036309
864.458182	553.604438	724.416198
392.769866	264.889524	121.017234
79.463777	93.0028597	75.6808219
51.9418087	57.9796204	49.9516904
322.571479	169.719096	192.597138
166.655101	172.793414	182.673802
194.305836	198.285142	163.906483
60.870951	49.746693	26.9575039
119.432049	132.054719	133.233791
567.462637	548.623125	697.68739
37.7607859	101.83782	84.9629295
193.963942	194.4472	195.518326
52.1970286	52.0020638	47.2975457
437.900986	450.98448	581.292438
180.441261	335.983322	632.261222
53.6062315	50.068128	61.5386068
52.5985395	66.4851327	77.5463352
67.311279	40.3938468	106.194401

N204 | N204 008 | Geometric Segment	N204 | N204 009 | Geometric Segment	N204 | N204 010 | Geometric Segment
473.556709	269.406663	595.67647
761.050388	500.340273	679.521734
231.918557	348.668883	262.386616
408.308517	535.30419	458.903802
545.611005	420.711543	580.071122
596.097093	363.366011	579.026461
177.390362	154.314228	150.258437
2390.90392	2003.26669	3074.06421
7024.49383	3116.17646	9535.66812
241.184566	364.568367	240.885182
58.7029701	34.8162217	69.6286413
6156.36289	2324.68619	7267.34883
4396.93921	10772.5437	2513.95584
429.820793	524.905688	426.004409
902.145983	915.973902	1152.25238
285.715659	281.095628	302.987745
246.725111	158.126227	293.626515
35.4103925	38.7903879	38.3205526
407.629544	127.027251	605.789193
82.2657756	38.4447073	88.5109368
1705.76259	334.933811	1682.78046
260.42817	175.025158	313.874134
114.049832	57.4493399	134.335736
147.749885	215.36251	136.439007
780.521186	424.22567	843.566627
267.579351	73.6878477	280.472581
97.2781313	95.3392335	93.6006817
56.3536053	39.9732967	73.0548976
250.819404	121.38414	310.541348
118.873663	100.706732	151.43426
205.79037	192.684704	233.634289
52.6494841	33.2911602	57.438889
126.142353	165.144832	92.0736603
605.58528	465.993025	646.652212
63.8632698	55.9984025	36.6215054
188.377722	123.381	196.856516
45.60778	39.526993	46.908595
550.178356	463.66313	511.942823
226.938759	281.542349	197.502001
51.4520285	66.4487303	62.2260703
73.1159625	70.3681765	79.412636
121.140903	83.7229285	82.8470799

N204 | N204 011 | Geometric Segment	N204 | N204 012 | Geometric Segment	N204 | N204 013 | Geometric Segment
397.302383	413.368285	374.223929
843.187184	3734.24622	714.816345
399.25562	218.486118	317.323358
644.542784	394.880297	553.214683
558.392756	442.417764	539.032382
659.060498	454.34447	598.788313
234.6051	184.937581	244.697664
2573.43784	2822.97851	2630.55277
5662.36554	5373.87958	5841.80834
335.196842	255.065957	310.808257
51.868489	84.8525691	60.1868003
3391.54073	3631.62286	3452.6461
5937.10114	2409.97866	2354.19434
686.496744	505.540716	602.968432
824.915501	536.214593	612.353211
327.921504	259.806033	293.651624
195.733607	212.436763	197.211344
28.8945599	42.1762949	38.4434412
157.702148	268.092578	404.490193
73.6381697	44.0911947	66.6727646
883.190144	930.075104	1167.60671
297.308898	202.57925	244.833074
59.4353066	76.7761576	72.5609547
179.86632	187.947239	213.929829
919.854435	604.398824	1080.99701
183.629718	167.668365	194.571724
91.3078437	70.1248014	77.1027658
64.3155043	53.559129	60.0559649
135.226612	172.617054	231.983296
238.340088	513.173029	187.087325
296.481309	235.61166	345.448699
44.8275911	36.5136047	35.4291543
121.363479	155.531068	140.867356
662.671033	660.403312	639.984388
104.725333	127.305281	140.163635
154.634204	128.983598	164.777979
49.0720824	40.1069435	47.6241957
831.384315	694.939385	891.301676
955.381963	606.501778	1145.41692
74.6954333	67.2915941	97.2546687
94.9431467	86.5368572	95.6264358
95.3807644	169.013543	255.308619

N204 | N204 014 | Geometric Segment	N204 | N204 015 | Geometric Segment	N206 | N206 001 | Geometric Segment
339.111814	3300.07003	259.011325
1171.34051	17924.3055	1250.18319
294.357265	4296.78514	467.688324
445.512268	12846.8905	6350.12048
403.703204	18191.7077	11556.1164
446.520529	7394.55567	22765.5099
171.860194	3197.97625	354.850049
2849.85046	74360.8419	13911.0485
3535.39382	32698.3781	24386.9398
275.951064	8767.79021	705.118305
86.0117646	349.520296	89.3867789
2041.98089	15025.7308	8477.97248
7566.90962	3353.99783	484.997146
538.099192	5172.28518	960.212403
560.864438	11662.2824	2485.9711
256.273799	4945.46801	1217.29874
179.832407	1746.92566	211.526884
42.3416429	854.443417	257.297288
190.338171	1699.07379	580.911224
33.4770125	726.826289	726.32435
910.39125	1126.7929	417.637615
195.760532	949.586359	1849.32587
70.2173434	487.165441	155.943384
200.655546	568.167188	383.550071
433.335509	16830.9101	19546.0048
149.740068	607.778228	557.422635
60.367613	813.093507	652.571991
50.6598222	632.633099	154.735815
138.095985	1389.87578	419.249432
213.790565	3795.44998	814.441382
229.659012	8837.47464	1090.84747
29.1097742	270.933375	82.1201518
126.700396	201.747861	88.5127462
546.777617	1274.42528	1174.55
86.4284587	725.808779	90.3747714
180.247482	1429.14861	402.715813
34.1607852	368.925796	87.5669104
604.505185	1347.82147	948.958307
498.595788	6107.67705	2803.72811
66.0270013	545.674688	166.24826
83.3943328	764.513102	180.012179
164.685669	1390.12174	4510.71986

N206 | N206 002 | Geometric Segment	N206 | N206 003 | Geometric Segment	N206 | N206 004 | Geometric Segment
320.078817	242.830049	204.965707
1419.52971	1141.28905	1192.60067
380.28447	445.079761	388.958838
6926.80144	5109.49699	5279.65229
16244.5689	11221.3925	12245.4119
31045.77	20861.6281	23207.6199
320.434039	350.702542	285.656002
17788.1611	10572.6823	8634.81519
25213.9638	17719.1106	17715.9013
613.343021	514.335058	596.734377
110.171138	82.4770805	76.6097634
9727.55862	9204.56708	6776.53618
573.12276	497.901178	462.278938
900.601353	821.090882	757.222039
1558.09227	1647.34261	1004.69991
1224.89369	1062.9914	1036.90005
165.329217	193.417279	143.346425
327.723933	273.92612	192.598002
543.690822	455.822052	427.593116
660.977565	566.008016	590.21901
645.016144	437.59523	375.72064
1651.92159	1206.36064	1095.26981
209.557019	175.613909	124.764347
490.772769	411.136334	339.822373
23929.4737	16706.5819	16513.3533
696.658259	543.924261	629.218717
661.686052	615.234381	616.03066
254.393916	148.762312	163.140048
556.302389	311.871798	335.159532
510.793386	344.948914	359.092498
1333.75402	849.985194	725.634911
110.39826	76.6732463	74.8993653
88.5522953	101.006681	82.5646314
1256.91327	1038.48852	1053.02881
115.844414	79.6925523	80.5547927
438.232605	323.146713	305.549592
109.507546	95.3851847	76.236866
1052.56656	751.013818	777.431994
3188.38689	2213.80402	2140.4966
177.478323	141.970669	151.29392
237.25436	151.045317	168.281071
3693.74498	6604.18306	4278.87797

N206 | N206 005 | Geometric Segment	N206 | N206 006 | Geometric Segment	N206 | N206 007 | Geometric Segment
370.243774	553.462241	394.10335
890.348132	3231.3143	1439.61243
542.072977	808.633639	561.961595
9131.21748	7815.55329	3292.35173
12588.3343	9999.61127	7922.36784
23509.3822	14721.7321	13020.818
495.142906	628.236099	300.561603
4477.25314	35652.4067	7504.06123
21390.956	28326.9598	16057.983
761.69154	741.564957	526.308487
134.397676	89.59628	91.71069
4757.83608	8129.72792	10330.5227
826.498654	1075.39796	643.840964
1510.43875	1743.8802	1082.43098
4781.82293	5372.12177	1388.47373
1030.59889	1594.93015	1331.75761
246.326402	453.336656	342.982455
332.356735	417.682504	167.298073
416.91651	484.128883	677.104061
705.726472	578.147058	440.969095
288.1136	300.963366	1931.5638
1037.30265	1250.46461	942.655695
169.862989	221.870726	266.603543
554.801828	481.031018	566.941764
20587.1714	22462.5198	14957.2341
479.096591	496.823471	478.10993
567.802943	524.72906	452.875745
230.05783	265.95817	175.571589
792.193978	780.55618	545.884746
336.055541	1273.23351	350.094032
938.838283	1583.44559	1029.23531
109.52686	149.868493	123.391208
85.4865921	81.9464256	205.142049
1205.37671	1341.85676	1035.84313
78.9721036	82.6908159	161.464315
268.610082	686.507363	445.645695
165.118897	170.533738	107.850614
1199.39523	1108.92025	1590.39959
2756.83306	2878.73512	4529.57489
256.418199	192.36978	130.024528
275.964697	232.898812	230.62131
3052.59163	2056.98267	3095.19749

N206 | N206 008 | Geometric Segment	N206 | N206 009 | Geometric Segment	N206 | N206 010 | Geometric Segment
370.997958	3504.54356	268.318341
2673.09068	30712.5189	477.233613
661.779447	5478.36121	184.255892
3037.53424	9112.85322	271.57691
5195.74659	11506.1714	287.545986
7505.6266	4835.00576	331.32031
384.130209	3661.58146	129.443099
7821.30114	61709.5379	2605.51565
17120.4555	24687.1713	6495.45531
630.021108	7257.66501	112.247586
63.9134621	248.66605	108.88377
5853.18839	9690.93537	2641.74113
716.376827	3949.18428	3465.09722
1063.45776	5009.85545	277.06965
1956.52985	11859.2627	853.904741
1337.76513	4423.00883	239.529923
445.81274	2212.32315	105.76631
148.976804	676.222466	33.3534551
474.597656	1770.98028	151.615463
329.435311	501.862002	61.1234932
600.852486	990.69934	430.575346
558.292862	599.160462	484.901386
361.06744	454.730834	79.6331924
476.681741	745.790104	129.39726
15038.4676	13959.4639	1068.8381
276.95172	526.484061	150.529045
348.342323	717.765682	69.5755552
131.901357	451.086713	53.6864091
359.085224	1423.60818	99.6492349
558.001581	2865.19449	61.0732524
924.872247	5794.07071	122.150892
103.854084	221.837819	58.0936409
216.080063	170.575949	64.9804665
1112.1073	1279.76961	421.779593
147.889971	473.344955	10.8019505
397.324299	1172.76755	174.471618
97.1371938	366.185011	10.992385
1270.34243	1310.50617	188.381851
4599.58889	4207.09189	594.776961
143.353088	440.470993	27.0716291
163.764849	560.203585	24.9948204
813.783031	1110.93314	145.47153

N24 N1 | N24 N1 001 | Geometric Segment	N24 N1 | N24 N1 002 | Geometric Segment	N24 N1 | N24 N1 003 | Geometric Segment
166.671411	233.452886	185.535848
637.459299	597.898073	711.443935
120.38365	146.235655	134.371169
215.287639	212.362083	199.478605
282.434123	267.398848	298.014601
204.456627	304.995266	230.584145
95.0126428	74.1961961	97.634341
1355.57943	1777.30636	1742.19948
4584.34808	6058.389	4093.94561
131.825518	141.547573	130.015555
75.1992154	56.1705138	59.0843569
1720.70947	2768.58197	1235.81387
1983.13843	1979.54158	2176.77485
276.939797	283.381314	297.50259
670.893052	592.334762	405.676465
189.882975	202.201719	164.596819
95.489355	109.124583	85.9306236
26.9299787	51.6187318	47.9282217
104.165006	175.201073	127.59699
41.9939291	50.4514201	61.8361345
275.608677	618.082637	475.328495
242.554859	457.062266	408.154111
39.0790894	79.8142141	40.5936022
116.010059	107.281493	98.8070109
935.372704	690.124121	598.091282
123.443951	187.441433	102.489779
47.3550896	63.0835291	49.6998734
28.8980327	32.824326	28.0528924
37.0869071	95.5729075	70.9137526
67.6764709	77.2410545	56.9351427
52.8179401	96.2406365	108.899828
39.7005806	35.1361977	30.9721539
69.3431829	79.9367364	50.5660283
499.705729	490.334493	469.468395
17.8396857	25.0760972	50.4939914
106.613335	159.760126	128.892787
8.71401257	6.80484792	21.136123
161.516183	188.160233	194.81383
620.714732	632.764537	585.461584
23.8450442	26.379469	19.5446562
25.4028047	17.8535433	42.2473123
53.3888426	39.6072193	52.3168401

N24 N1 | N24 N1 004 | Geometric Segment	N24 N1 | N24 N1 005 | Geometric Segment	N24 N1 | N24 N1 006 | Geometric Segment
112.890397	90.4556858	109.99721
457.232926	414.643044	521.432158
135.833685	137.305157	147.923383
268.379223	243.507309	338.928833
256.50967	189.277869	232.334658
247.296802	169.178302	201.131074
100.069266	78.6405274	95.8795847
1837.77455	1403.5827	4891.32976
3619.18307	3304.46978	4020.25362
115.07186	118.71899	132.822354
33.6432638	33.4893075	18.0295746
990.04774	1014.07441	1035.12828
287.134706	221.280571	178.695598
309.21004	327.655749	304.689874
198.542819	211.073085	259.822113
204.580143	156.973791	191.489188
60.5506458	70.4953889	80.8502579
30.0199254	38.445386	39.0007551
45.1725117	52.7938496	66.022227
39.3558672	37.3102564	47.4775402
77.9750278	72.4188977	103.604539
177.663661	215.649044	209.103709
29.8477648	39.9624021	42.0085147
58.4497248	68.4497067	72.0272245
1096.3013	1031.34705	755.079569
48.145989	16.8160232	17.3748482
52.7849782	53.1816061	59.3448347
30.1216769	23.6714494	40.3558514
10.5198046	29.394146	10.2502001
71.3228522	54.1815157	64.2828474
111.084202	110.269561	101.11643
21.8243955	22.86792	18.9022878
91.497236	70.0604092	126.870608
494.007947	587.467532	548.201175
26.1740688	25.7114729	30.285136
80.1112052	64.4203665	88.9499198
21.6632415	7.56635349	10.5540249
204.036759	182.352591	198.807647
1037.25096	927.662076	940.908092
14.2821345	29.9300578	27.8322159
35.2822371	39.2798205	17.189007
131.074977	179.307968	47.8934065

N24 N1 | N24 N1 007 | Geometric Segment	N24 N1 | N24 N1 008 | Geometric Segment	N24 N1 | N24 N1 009 | Geometric Segment
97.846288	78.8382806	332.661203
639.645405	546.924982	544.535555
146.150175	122.713784	174.421836
358.040371	259.049747	237.462937
258.822951	162.441355	273.542248
205.606386	159.006565	335.704459
77.9606711	114.590754	119.471135
10627.3175	2431.27262	1615.65568
3177.24131	3560.48839	5401.41162
156.510437	107.32434	180.341298
27.3499403	13.5971946	83.7463698
720.633744	750.254486	2722.18995
329.427852	144.12387	1914.56774
314.211345	301.833633	363.203503
321.039437	196.21383	749.226117
224.258338	177.409738	241.175076
66.087799	63.446	86.7718621
67.7899588	43.8128649	31.3540644
98.8683289	52.66394	217.677284
83.7936567	41.6585427	45.1317569
212.497167	94.9008128	872.151597
337.837079	174.178486	549.456126
55.6059831	28.4825868	69.9985291
83.8519306	81.638048	167.05818
757.617341	1007.50365	558.6617
32.6164829	16.2154893	239.539092
61.8908616	73.8466333	61.6210619
21.0436041	41.8478398	33.6454324
35.6331892	14.172211	66.928355
74.2883723	75.667468	75.7638787
81.6901459	97.4706457	112.740701
29.5698578	12.2507004	46.2226344
69.7328658	74.6698231	111.491128
503.990315	544.699892	456.909361
28.2597589	18.1817274	37.386735
124.950219	75.2967102	157.675965
11.0068163	16.4163236	10.1455599
199.714261	210.705323	209.629769
903.199023	959.814932	370.243285
22.8063633	26.7996814	32.3893892
35.8529082	15.5964362	17.7456027
77.6971088	34.948786	71.4835616

N24 N1 | N24 N1 010 | Geometric Segment	N24 N1 | N24 N1 011 | Geometric Segment	N24 N1 | N24 N1 012 | Geometric Segment
127.444151	158.19377	288.335624
326.743947	540.314489	442.167177
180.066907	188.161843	211.613416
489.949752	372.998039	347.714709
423.535914	423.01828	387.12843
593.263102	450.401259	366.822978
141.159736	139.192378	127.520862
1339.69046	1019.92532	1093.44208
4059.66845	9951.15612	3993.37106
217.234527	155.298631	224.077742
39.5880962	16.1436783	36.409824
1394.18547	4505.31818	4368.9482
783.331045	243.008885	4867.56458
373.927862	292.634677	306.461426
302.731239	233.472718	843.449294
175.648966	219.115624	195.176731
89.1376883	110.150204	108.549446
43.4837787	48.9691399	20.2479003
78.2308893	106.492208	125.807724
87.9679558	60.2854076	58.9358905
326.170826	291.424807	578.028989
495.790261	339.881142	192.274293
36.2938543	86.9438438	39.6057006
107.59478	133.172652	181.50927
605.984518	1310.73454	387.492796
98.6999498	151.812528	217.199799
52.0792474	55.5069982	49.7275843
40.7852058	37.8261831	31.9536661
37.4151523	43.0679613	38.4737805
80.3409636	67.9175783	71.8208389
161.256903	100.72411	114.586248
18.8684348	16.0882537	23.8797542
101.461939	37.542929	83.2411946
571.99268	580.236219	391.217208
93.302872	30.6542598	83.4941215
149.714911	149.595791	119.586906
43.273291	47.339754	44.1378506
537.014615	434.126503	451.41506
1073.13398	1274.01247	406.551654
73.5501345	57.0116768	85.4533996
58.0562441	59.8374748	46.4845388
376.67151	230.448193	82.1434306

N24 N1 | N24 N1 013 | Geometric Segment	N24 N1 | N24 N1 014 | Geometric Segment	N24 N1 | N24 N1 015 | Geometric Segment
366.80753	142.566017	390.702211
718.890842	350.709182	421.9998
209.614272	171.408933	126.010355
418.571338	479.574079	247.148827
506.662875	421.846451	356.551643
562.441279	652.132408	431.765435
113.536755	102.427474	89.8653657
1129.46401	1978.59662	1177.90646
4714.73968	8211.97568	5831.39728
230.916767	238.909133	146.522361
40.9009547	35.6989365	21.1121356
2042.04592	2830.07149	2202.60322
3940.62212	367.863	4164.08348
312.703387	295.297234	200.849574
581.570079	304.86886	338.409829
169.538779	198.453611	148.413016
101.827245	116.743463	94.4865026
37.0901695	69.2814111	14.1633708
121.704784	63.0311987	125.323089
60.037704	99.8722965	30.6257122
790.35575	204.335984	640.780713
259.764159	386.344023	277.485517
42.0703276	40.9660641	40.8271216
190.096501	114.406311	160.827866
439.020044	1114.47743	399.442889
272.562332	150.520801	460.690933
49.0490808	50.6319295	48.7966805
38.1529776	29.6808474	29.2551234
63.9708823	48.5004794	25.495903
86.7601863	83.417177	46.7922637
164.100392	118.059042	93.7009879
22.7127173	21.5022044	25.7151317
107.624669	62.3936554	13.0451828
447.705243	536.768189	247.316684
112.121723	34.2391315	12.4437167
141.003934	155.950962	115.304549
34.0713514	36.2873933	32.6406163
548.275731	348.130145	320.945311
665.116921	1024.05631	90.3880444
74.5992271	52.0044552	45.5631422
65.8173442	36.860618	31.8809938
111.631182	341.71807	61.3906038

N24 N1 | N24 N1 016 | Geometric Segment	N24 N2 | N24 N2002 | Geometric Segment	N24 N2 | N24 N2003 | Geometric Segment
698.584431	102.056026	256.369171
521.747555	510.52873	438.653256
174.604394	147.598498	160.421482
343.878287	1066.0853	368.790829
635.794736	664.361938	337.755137
761.062879	974.265735	409.441594
128.961056	102.763029	101.089658
2165.02358	1278.61512	1322.28421
9296.61593	5275.02208	4410.61835
184.108893	186.113637	168.309195
49.2134827	19.9545657	45.8081249
5891.23574	1158.9087	3792.77012
1352.96676	210.787364	2663.57704
262.951519	332.128249	187.02211
564.377106	295.756292	536.114637
216.141776	171.269858	157.641406
163.97713	84.6762781	113.07516
33.4104745	62.9233743	24.2613315
254.478188	35.4947037	171.269128
76.9303763	98.2443686	71.9607461
1322.8278	119.13521	924.496506
386.610709	293.581183	380.011039
93.5895141	33.1370926	55.1951319
160.133611	92.8566276	178.583767
633.140139	1077.5094	560.422114
631.313556	120.811128	239.213817
86.7207881	68.2141897	47.1632822
34.7175611	32.5431902	33.0077275
126.428651	45.8788328	110.639637
128.510409	118.79962	82.8465755
132.406971	158.02064	86.4983225
33.9539002	16.1794846	24.0349806
77.1282752	96.4579174	105.293959
473.281916	577.344311	386.146102
26.6995778	48.8551818	43.5371689
177.348089	132.992582	134.890982
36.7858485	29.1250173	25.1096477
402.638332	425.841496	376.842744
173.502464	970.173756	195.866695
44.173724	38.0130854	54.3734267
43.6684738	31.8608763	32.4488429
71.3280381	171.765074	79.9214772

N24 N2	N24 N2004	Geometric Segment	N24 N2	N24 N2005	Geometric Segment	N24 N2	N24 N2006	Geometric Segment
241.986726	107.078957	358.548858
1174.4315	405.356808	692.559222
136.192414	168.165238	85.2257507
267.032499	361.207642	187.707644
358.099083	212.941469	396.40778
325.343929	209.109387	327.368236
87.2359919	130.463115	94.7942079
1821.6937	1395.45612	1905.50377
4837.19699	3672.1628	5447.68601
147.376584	182.055139	91.7109899
38.5246597	15.5352623	65.149545
3571.46678	1310.29908	2166.12003
2741.23074	176.73288	2053.54915
201.266066	286.913201	288.251657
517.252173	226.216081	469.840276
175.468916	179.217229	216.971554
120.760175	96.3474303	99.9446093
33.4862264	22.6051612	54.0724305
358.688152	39.1185609	327.885352
76.8514701	42.178651	78.9807117
1539.57294	86.5375848	1273.36348
336.822273	129.064486	514.497615
51.1975788	34.4819084	70.6361052
118.810812	94.8037593	121.720569
375.453107	479.07041	739.396149
230.594234	29.4187992	358.515769
40.7119056	31.6976296	72.9837939
24.4936924	21.7538427	42.8654371
268.470844	15.9962519	78.5589881
166.349732	70.4584578	77.6058849
69.1243408	77.4938652	98.0434777
34.3968077	12.4106131	27.8758489
91.9969143	68.2050856	93.4157127
458.872895	454.319768	383.293805
42.8257739	20.3313739	16.8207299
220.213648	138.906735	239.160532
35.474077	34.1314655	17.8594239
412.518105	355.640463	261.581912
152.949957	1164.68705	256.307755
63.8236888	52.4086458	34.4813084
36.1184503	45.4597466	47.6623729
172.806808	50.9129578	104.347969

N24 N2 | N24 N2 001 | Geometric Segment	N24 N2 | N24 N2 007 | Geometric Segment	N24 N2 | N24 N2 008 | Geometric Segment
242.936578	108.292091	106.196427
999.520256	539.973769	599.42658
95.8232545	160.044745	124.048394
140.20785	240.82529	261.400004
276.720211	259.708096	368.450947
199.731096	224.908527	312.030025
99.0044275	116.675275	68.3873993
1438.08733	1878.62599	5113.08277
4120.72279	3037.58544	4377.06575
98.7303244	111.55288	84.392251
59.6206547	18.015916	29.4164443
1974.53148	870.924605	1083.00501
2974.44484	6005.65224	241.984601
348.280079	250.5757	370.115054
747.23029	489.312039	248.267491
192.988998	154.001106	167.256393
98.5910962	77.0004438	74.1464368
51.0207148	40.4702096	64.3855295
299.184381	86.201322	62.8488742
52.5940242	48.3136216	60.3626465
1313.97659	211.390132	163.845469
408.650792	336.209516	310.193308
43.070211	23.6632241	41.6094935
119.293433	129.771298	69.7248106
320.40717	616.774651	1313.63691
151.855128	18.8946211	49.8365487
34.4686741	37.276875	46.2690235
44.4838304	33.0878395	45.454507
148.541898	13.1372954	16.6838134
69.5026635	86.0570552	70.2571152
107.01637	87.773898	132.002957
36.1974627	33.3348432	40.2985695
90.3206394	64.9826355	43.4209608
407.222811	339.81124	650.035029
23.2364848	11.2344035	39.5092265
287.114066	151.969852	155.548475
13.2418359	9.1459696	10.0514399
262.109662	160.490662	227.23187
141.386388	433.252777	903.618764
23.2939225	29.7196248	27.5047836
33.0875721	27.9951333	26.3714663
45.2009012	168.106046	158.884054

N24 N2 | N24 N2 009 | Geometric Segment	N24 N2 | N24 N2 010 | Geometric Segment	N24 N3 | N24 N3 001 | Geometric Segment
309.671311	220.502494	369.002005
861.435554	1116.31591	813.580761
129.702019	89.6480421	133.261631
243.288115	233.667303	215.023694
358.346176	393.016716	412.647102
334.055108	385.144685	458.874356
81.6012459	91.4367184	75.7271327
1633.18935	2580.92079	2162.88317
5423.46839	6047.76041	7055.38332
125.54398	118.935088	150.096845
81.5232867	87.0900174	190.788159
1985.61799	1932.85012	2583.85232
1307.33884	2004.45122	1216.86622
335.122364	341.898614	289.953382
441.91306	498.820486	639.165236
185.318487	210.127178	212.925425
111.304802	126.076128	99.4430522
55.9565552	69.4190194	41.8125504
286.736275	160.64843	288.903574
40.2725722	74.9424924	96.5482798
797.82858	485.779828	749.318819
337.821734	437.220656	442.778218
56.2158204	33.5788366	77.0115165
137.441889	115.421294	99.9360032
620.511304	1116.86056	968.197779
201.873794	135.533407	283.059278
34.7283342	81.3800343	87.2212909
49.1284501	40.6321037	33.6511717
123.297866	68.514152	95.0112888
81.1279637	105.119533	96.3207358
107.715581	100.85331	162.875458
38.4962654	40.935426	52.7370366
45.7412658	122.004021	89.2935658
360.601678	573.247741	484.068031
17.2985197	24.5260941	10.156154
131.718703	195.59516	237.859162
12.070962	17.4709362	16.5363253
159.550866	218.413084	199.730397
203.311036	832.868597	552.690276
19.2680883	25.6111663	16.9687646
29.9105429	34.9239101	21.6927698
40.6758744	94.0428057	83.5844096

N24 N3 | N24 N3 002 | Geometric Segment	N24 N3 | N24 N3 003 | Geometric Segment	N24 N3 | N24 N3 004 | Geometric Segment
338.843112	252.01391	89.8077426
758.92073	737.72117	708.033476
111.06135	117.093919	138.99955
156.802215	218.0113	334.927915
357.654616	328.462202	379.21017
289.418773	346.788603	448.539485
83.9250281	101.45032	50.0255795
1509.33966	1585.62909	5007.00417
7320.01872	7559.39841	4039.03334
157.196674	79.5815752	118.724399
69.3117417	77.7329653	56.6268782
3164.00283	4052.52793	897.763923
1713.10844	1736.0264	225.195916
286.798177	255.813112	304.328301
367.655354	656.143217	180.222061
190.595855	203.780607	227.218705
123.433331	146.767485	45.1779242
28.1123256	41.4008615	67.8422829
278.387249	297.692472	90.6672519
51.3600881	68.5349646	71.6127373
1254.68421	1381.83733	168.90578
510.007267	446.451736	454.798096
84.9691273	78.010533	45.8163936
113.258665	168.335349	74.0247987
627.711276	564.613173	1521.14079
287.740514	261.543887	68.8909937
39.3684051	41.4928426	61.7539665
39.448874	39.5209472	34.5801069
127.760123	141.581804	37.255102
68.975635	64.5360529	93.7701989
150.33048	114.321611	99.6098239
58.4461225	53.7534988	36.6718426
72.9179722	78.5276854	34.3141207
455.257683	446.635011	512.937599
9.45471725	14.5960717	14.8308343
307.967772	283.217265	163.689489
20.5256544	21.6963477	14.0861434
231.542916	162.132778	157.754515
180.959303	142.977392	974.43076
14.0416121	24.9340968	26.1557769
29.1699119	48.6572864	29.9176673
75.4537078	69.2419963	549.2551

N24 N3 | N24 N3 005 | Geometric Segment	N24 N3 | N24 N3 006 | Geometric Segment	N24 N3 | N24 N3 007 | Geometric Segment
138.117273	205.891495	194.545767
695.418644	432.628287	418.086763
212.471789	218.361834	184.059496
321.501674	356.357839	296.438756
313.801904	973.561885	461.359356
350.803292	1150.7597	603.909614
168.096396	145.424219	130.10226
1992.76872	1723.88353	1120.99503
2917.34546	5454.57749	5176.2748
231.825626	198.117291	192.966787
65.0907174	33.5020332	34.1009408
2196.50189	3701.61536	4122.39487
3505.00861	3381.33445	1915.54825
560.00541	442.446573	480.568056
319.334302	276.454934	262.907209
246.251887	261.065434	240.865334
147.510613	167.341814	125.37799
31.6944021	43.5502986	32.3185607
197.741185	307.800595	179.070471
84.4088003	63.5141467	37.4926993
993.793539	1517.09846	726.992502
517.944302	748.056055	474.19679
50.6564133	74.0132362	80.4715489
257.360559	209.627968	200.114971
442.579805	778.350896	552.230121
69.8884165	225.639685	196.838834
89.3724614	78.5790361	71.3347085
56.301478	38.5504431	48.0208755
135.988751	163.993767	89.8483392
182.936438	114.160047	103.544059
311.989305	238.764771	224.69328
55.3808279	54.1839955	42.2202887
134.176173	83.8703909	86.7325839
596.460489	431.769327	510.957437
140.243381	76.3449089	96.286863
294.057581	272.849288	162.669128
69.0279941	43.5297574	59.288405
891.539437	463.541533	556.360216
1325.74547	527.762526	862.344657
43.4363908	48.7235431	37.0615519
99.8830497	57.4840083	78.800153
313.838177	773.403	390.441082

N24 N3 | N24 N3 008 | Geometric Segment	N24 N3 | N24 N3 009 | Geometric Segment	N24 N3 | N24 N3 010 | Geometric Segment
155.562273	136.34443	162.62695
321.27782	344.930106	767.740554
192.675266	193.962208	220.305439
262.066322	313.578598	351.514365
369.52765	257.434096	326.897981
495.901608	345.777214	313.939763
153.243229	126.999161	187.809815
1123.72704	1398.0351	2005.3008
3812.40535	2893.47861	3232.19232
149.188691	145.855259	201.460439
34.0530761	36.0075226	40.0725174
1854.71033	1652.10259	2296.23158
2638.25096	4068.491	2990.57943
385.850116	339.798646	545.476476
270.516206	316.689052	349.027894
257.279407	181.044879	270.096889
107.347112	117.30197	157.410219
30.5192196	21.0030978	39.0247498
229.831481	101.503854	240.210188
48.780618	39.0854544	71.1432744
1355.086	639.016893	1168.67398
727.857418	295.445168	654.913606
45.0318711	24.3251844	58.9072049
198.246351	220.333351	271.614383
341.610215	281.018587	479.943726
167.095567	78.5962512	106.408827
69.8580681	55.076318	91.7022595
46.3121418	52.7200862	36.10573
293.294878	68.5668168	227.905143
81.4826891	79.6342964	159.14145
157.015749	135.200805	335.738096
32.117232	36.7688826	76.5895678
69.8403927	111.680975	152.971037
426.11255	366.719931	617.473635
132.037867	94.2827224	158.888814
252.448446	133.325022	299.384256
57.2895488	38.185584	56.6619553
411.412413	458.112237	1040.05294
240.063627	450.313675	1316.84531
39.8352859	33.3730131	42.446389
50.4789547	44.3340984	96.9470128
264.80134	138.570253	271.834592

N25 N1 | N25 N1 001 | Geometric Segment	N25 N1 | N25 N1 002 | Geometric Segment	N25 N1 | N25 N1 003 | Geometric Segment
177.273823	79.4165155	147.844404
626.923501	596.21368	439.711076
198.314232	147.87506	191.30913
328.129819	224.170922	266.711558
379.613738	200.872752	287.798704
306.031048	161.632624	251.44998
156.802961	108.430585	150.005691
1687.26555	1386.10216	1556.73534
3849.58717	2891.55663	4816.76822
223.143235	147.822478	179.176103
31.9279299	27.2913999	27.2010559
2389.51387	1289.36348	4261.24388
1162.76149	442.381445	1051.22832
533.119985	370.387143	454.970234
251.02252	184.716245	312.768899
253.64849	193.991478	210.693869
150.277992	92.7729318	150.25696
26.9075052	33.2222719	37.2513315
156.512526	164.02284	94.9827163
82.4662135	41.3220662	53.1471759
850.073588	615.614603	599.871816
291.171079	170.846584	365.526084
66.8976454	27.229934	66.1532467
232.363074	154.152547	196.854132
647.001445	392.374283	409.512401
102.078224	36.3560686	72.1317249
93.6719518	49.042287	37.2834237
63.0669421	39.7220141	57.4416033
91.9323896	67.6171512	29.5984154
135.049583	90.5866609	99.9746399
311.047208	151.345575	250.091121
52.2405575	31.0638301	48.7393836
147.662614	63.1096262	149.264387
766.093648	531.388654	528.559615
151.583589	68.3191645	112.55891
322.53904	170.934226	193.49634
80.7412183	42.3001841	79.3276616
1049.89372	580.35307	678.545114
1971.74765	1459.15456	1028.83442
50.7289817	30.8538652	47.270393
114.591115	63.1235594	75.7958202
271.051943	405.04697	126.414063

N25 N1 | N25 N1 004 | Geometric Segment	N25 N1 | N25 N1 005 | Geometric Segment	N25 N1 | N25 N1 006 | Geometric Segment
164.211361	154.841462	117.610496
525.661563	317.999683	393.441739
192.062957	146.036095	139.137554
303.321417	272.868899	625.526093
299.918144	333.182834	1585.71197
348.32295	400.49805	2078.9881
130.739436	128.449297	125.218456
1448.45185	1655.81901	9063.34048
3235.93363	3194.36312	9088.5244
168.147563	138.923204	248.60742
46.0324526	28.1665022	42.4592468
1664.72987	891.293274	4744.99166
6002.2583	3008.85345	205.262678
353.838488	332.026356	483.196361
380.732817	199.373386	227.815653
187.917456	166.226301	417.137865
104.320179	139.062147	138.98801
25.1590495	29.3893271	201.002443
122.194915	224.808994	193.564837
33.7194417	51.2481521	142.021394
474.191703	1202.18866	399.152691
403.971403	440.656515	1115.45497
37.7740766	27.4004888	104.026222
277.254894	208.812803	210.9585
325.436738	273.108286	2954.38545
71.5838683	102.783015	235.569512
56.4326901	56.5912214	187.43069
45.7079032	28.5505807	106.267213
92.4100408	165.504336	199.863764
76.7688712	92.1912015	146.521614
168.399735	127.401278	297.211629
44.3088796	45.5851643	63.9902426
91.4472814	65.2809389	96.0486109
388.153952	427.979365	728.553311
85.3915979	23.7058902	38.8233752
150.516027	246.601969	268.476795
34.8692233	40.5382203	63.3721313
519.407068	387.241107	497.322812
366.024636	237.290119	1087.26202
41.7937604	36.7610668	71.2096358
52.5984341	46.9351728	94.5687255
338.245376	247.235333	8687.52722

N25 N1 | N25 N1 007 | Geometric Segment	N25 N1 | N25 N1 008 | Geometric Segment	N25 N1 | N25 N1 009 | Geometric Segment
190.356213	117.897644	141.357647
327.741766	537.333566	1129.24659
174.599052	171.537634	221.877849
280.102855	298.541378	957.834833
329.412916	286.542107	2286.26603
435.577636	284.888978	2776.82404
136.834807	153.107712	175.706111
1208.2135	1763.73995	11081.7797
3681.66718	3379.40073	6996.38356
166.204236	164.788086	244.793017
48.468726	27.4805586	35.6268414
2926.37565	1824.192	2487.35295
2452.42426	652.543746	298.031501
401.377583	317.779433	681.97626
316.827006	302.854951	372.051126
214.16659	203.14949	469.613242
131.100951	117.288912	171.56965
26.6459655	45.7184685	132.817999
144.678703	98.3576729	239.089928
59.7399008	60.4546631	149.532632
637.371118	458.847784	404.618518
336.165007	270.576735	1100.68047
52.7275251	39.2086935	95.3093278
231.821713	165.569056	245.25442
419.444238	466.392405	2776.88813
147.56755	53.1426935	160.029441
74.5404627	56.7727365	191.082985
35.9227517	37.1403781	75.2346857
84.3848114	78.1453359	182.203987
81.197408	158.804755	537.375378
243.711173	143.676495	316.712891
46.270314	47.4400227	55.5998552
141.750993	76.0547158	82.8751902
543.667586	539.611751	758.326416
75.4449983	86.3466864	119.939066
162.896027	160.39762	502.96836
63.0071307	49.5428144	59.0341809
651.321081	589.370203	774.604263
775.776313	1148.93496	1604.7147
44.7154699	34.9301884	58.3749694
64.8441681	64.5838954	83.1133639
281.47106	375.700343	1648.74874

N25 N1 | N25 N1 010 | Geometric Segment	N25 N2 | N25 N2 001 | Geometric Segment	N25 N2 | N25 N2 002 | Geometric Segment
343.19501	106.223937	150.497237
392.540768	1060.2593	712.784419
229.294071	235.049446	221.363956
332.349324	598.460564	645.432385
490.74276	709.102352	791.152463
538.21629	996.557844	1352.87852
158.656217	162.676542	162.278113
3052.67235	3644.17544	5159.23229
6202.10567	5028.17004	5275.51451
209.864959	233.285082	191.81749
49.6012841	13.9270884	16.3474969
6103.52979	1130.54788	2273.90104
3433.44443	279.218299	285.769071
469.339871	613.045704	493.37442
878.711146	425.120059	637.948319
261.518834	336.776432	417.052643
144.845227	117.896422	109.334994
26.4771755	52.1966407	73.9838235
282.968017	109.464736	122.830324
87.9931438	103.61329	179.047263
1646.37609	293.809475	231.504117
561.544799	437.042252	584.72297
71.1829223	57.7630857	53.1177331
196.486392	161.872976	191.306836
439.658803	2039.71055	2971.67692
132.574963	92.0638273	72.0357311
69.2982072	116.277096	98.4160654
50.0229495	53.9048174	60.6276585
393.586819	81.2144357	146.680665
163.716269	167.230593	251.660202
162.594064	191.124542	253.765326
68.7564248	40.7237441	32.857887
117.159927	110.760087	122.187437
431.943279	752.591157	714.941276
74.0368191	56.4556792	50.6788214
267.809009	176.505427	216.966092
49.8701573	44.5370913	59.1700016
410.55059	432.639529	541.587216
355.579711	1848.94198	2116.70705
40.5592336	47.6908549	63.8666341
60.0252306	65.4943996	80.9304598
63.9871659	270.107154	273.256595

nSLN

N25 N2 | N25 N2 003 | Geometric Segment	N25 N2 | N25 N2 004 | Geometric Segment	N25 N2 | N25 N2 005 | Geometric Segment
391.594354	262.244136	167.840079
488.48223	451.032441	844.226954
235.724135	211.087789	263.149164
363.989463	447.232042	412.926233
461.91529	401.637913	379.909889
819.738076	531.316454	513.607892
130.32039	142.579651	164.18774
3288.89925	2024.1872	1993.10601
5299.91841	4823.95011	3506.86842
220.490275	210.397689	237.854778
51.3054318	34.5578884	22.5320705
3049.95774	2940.54298	1067.5451
2336.67524	743.365723	285.876351
470.245134	488.135055	493.046281
508.419627	511.137588	422.319
284.567962	249.942366	266.131446
134.75421	157.477224	110.334661
36.5624952	42.4080669	44.4388382
258.255819	143.352796	65.1821051
121.510276	70.9683802	78.4942238
1167.80765	529.896411	231.956062
629.859865	318.942301	388.070951
58.8374641	46.3190263	48.7813657
172.482283	169.289676	140.577864
958.293707	1005.571	1051.77375
162.201263	76.7642814	66.3707588
96.5942691	80.9804084	65.3913521
56.5524717	53.1206882	29.3667721
298.423977	157.806773	49.5946191
190.508689	119.298678	174.107527
197.205093	139.027287	147.104322
62.7517803	35.0294601	38.390525
108.978778	125.7949	83.7411016
552.780263	594.815211	827.672085
65.491918	39.0588321	54.80241
262.294615	158.125618	131.553297
43.6942949	56.0266608	41.0978949
502.464851	391.526015	490.420751
1253.58295	1364.73006	2019.06206
74.0342192	47.0969483	52.9252194
74.1065931	64.1562855	77.9988696
61.1326025	114.178832	96.3799359

N25 N2 | N25 N2 006 | Geometric Segment	N25 N2 | N25 N2 007 | Geometric Segment	N25 N2 | N25 N2 008 | Geometric Segment
145.621602	137.461638	209.903508
545.449395	509.780828	806.077074
277.117954	244.065839	310.823519
2112.32604	2363.27435	850.5631
2776.13957	2444.49188	1420.53701
6010.30017	6475.70849	2095.58393
177.653437	191.64616	210.62044
3355.68479	3713.0992	3316.9489
8947.82386	8663.43776	6730.8721
375.721293	332.022837	339.160743
40.1553208	29.0250923	19.7808045
2169.56068	1380.76997	1708.27974
326.346266	400.562211	513.14123
666.440824	520.899191	574.086687
615.424062	393.553606	633.008354
417.434619	375.319661	439.106932
99.901688	80.051047	135.91969
113.04628	93.0687854	93.6303802
95.3418924	69.5804689	145.109094
305.856276	265.295053	135.950721
111.554204	110.583324	455.605485
1007.28647	373.223927	369.99013
54.0694904	35.3122247	106.540104
156.099174	182.536609	250.622676
4550.50898	4706.81777	4779.41719
245.857655	210.175286	157.819232
321.415441	294.44225	135.554067
69.7809613	56.4918087	60.7284803
194.130441	100.757666	102.005412
177.464657	148.702312	211.128482
430.007334	326.116412	302.381807
55.067598	23.4481704	53.2669768
103.318912	64.7235645	104.053331
599.558025	437.877227	630.072884
55.6151376	57.4170316	56.6638216
145.332901	128.23834	223.437901
49.046138	42.2015345	52.5732175
404.189541	270.297761	382.945156
1409.63088	796.057247	1154.35439
56.5921138	53.5496001	54.7416435
88.5173114	65.5177055	60.4645939
968.848473	678.809531	272.872142

N25 N2 | N25 N2 009 | Geometric Segment	N25 N2 | N25 N2 010 | Geometric Segment	N27 N1 | N27 N1 001 | Geometric Segment
151.517102	191.997835	122.641298
12879.36	8315.27729	1503.38529
370.645999	366.236296	282.91195
1186.96489	1137.81639	525.644597
1800.05824	1717.99463	785.603361
2396.68193	2229.6726	921.944058
254.649811	259.95581	191.569763
13051.7724	11751.6216	5299.46256
7358.15092	7025.13833	6019.52972
340.928517	389.468853	259.180775
25.2694772	20.4053532	14.5724024
1358.07658	1396.62812	1097.55517
281.18902	265.560346	172.350355
642.699026	605.977178	555.270568
1088.7382	1071.06381	460.883893
610.395307	620.689214	465.897443
173.281766	203.870231	142.945972
301.295752	240.168335	76.7646708
400.149073	367.54411	144.125477
244.870307	196.388615	100.154097
571.470298	445.64683	397.891254
286.947646	230.894872	145.423406
114.438948	124.988801	115.422748
251.679822	248.742686	246.735827
7059.61373	6629.3129	5180.28696
168.884091	145.972377	92.7172595
177.842564	161.420374	105.591665
68.4521445	94.5598332	59.0883398
594.524648	561.419622	159.457817
2234.04244	1320.37941	363.567998
512.679867	470.059751	243.182832
58.9297779	44.0947164	37.788081
66.2927676	47.6412516	85.8208063
570.403111	538.440075	558.284399
63.8705281	44.1153928	46.4696293
366.625135	319.723401	253.158919
61.0025977	67.7914209	60.3738281
279.083717	242.640687	336.833725
1036.52468	1170.13774	1096.4679
65.1318738	47.0583562	41.8214709
87.3778155	47.9797472	58.2497296
503.091049	410.048245	218.164825

N27 N1 | N27 N1 002 | Geometric Segment	N27 N1 | N27 N1 003 | Geometric Segment	N27 N1 | N27 N1 004 | Geometric Segment
302.487861	199.543039	299.326312
435.974497	587.990483	327.553676
287.792513	300.296338	251.200782
430.390549	503.713124	442.970961
428.017131	386.567079	403.216685
552.610131	450.886158	605.718433
189.99452	193.390049	190.948241
1974.67372	1927.58549	1523.0429
4713.65085	4237.70847	4490.3034
278.510705	320.175416	248.059228
46.7257335	13.394053	43.4353495
2863.87535	1161.33907	2686.97283
1704.97266	423.683346	2521.98359
357.385856	674.323639	320.206536
751.480014	504.475174	684.441866
337.809619	310.884134	244.236525
175.723444	146.274194	149.301555
34.5353621	39.0435919	24.4407528
120.778737	47.8523461	76.2247994
98.139562	69.8827809	62.3548383
773.821932	216.140696	601.875394
383.915956	239.041826	279.248512
70.9491713	40.5068875	66.5345908
237.70035	177.766951	199.750718
947.630065	671.285064	547.173136
105.156148	55.3376515	112.956881
100.249336	81.0487741	90.7891926
72.613782	42.3197576	57.0988173
160.922647	37.9044273	106.15746
115.946871	124.277827	93.8087914
151.609978	188.462986	122.762149
51.5671929	36.4326631	38.3373598
113.042656	114.875518	94.9950295
460.683037	623.006552	397.653428
62.2130618	108.58987	48.5486434
208.511965	191.94806	169.262601
49.8700357	67.1042666	32.0846687
369.81621	551.726447	306.201771
653.53424	1561.38901	406.487209
47.0250837	52.4177935	48.322879
82.5641475	134.564662	74.427569
142.061817	261.722419	77.9546176

N27 N1 | N27 N1 005 | Geometric Segment	N27 N1 | N27 N1 006 | Geometric Segment	N27 N1 | N27 N1 007 | Geometric Segment
202.540557	111.405345	227.946404
682.922513	2309.06844	526.133294
337.128849	187.951195	138.677124
843.133772	390.721223	509.897346
676.780542	380.672906	435.431119
1288.39599	326.275592	406.688607
264.210349	120.907279	88.6556244
4467.61374	1632.96543	1949.84463
8393.62008	4893.37737	4318.38739
374.858149	172.69828	196.002286
26.4610129	14.145238	42.5994421
1475.73362	1445.7146	2177.56348
371.746919	133.519192	1622.27775
578.397381	364.34116	426.148236
616.961847	303.374519	515.935088
505.196931	242.137022	321.711949
195.25643	89.5353262	102.354028
82.2497206	57.2449646	93.4283182
91.6099836	63.0944267	150.784974
108.474905	45.2943311	62.201713
340.412203	332.036998	935.467406
464.843797	140.352709	806.661883
108.985837	70.5512867	81.3716717
133.886649	163.09659	251.885938
2116.15254	2226.44465	1148.23144
130.754873	48.2028873	171.560527
168.804619	60.5399532	47.4033551
59.1214099	46.0133688	58.2005346
67.7514661	18.3956079	36.0098368
117.258482	223.14824	96.7719762
196.230495	70.432138	120.740478
49.0119647	13.277764	31.9895835
56.1960751	48.258546	68.514704
648.259489	213.059553	215.288708
72.5308588	13.2105742	35.8061688
239.170439	47.8560948	137.889897
56.1440471	10.7947627	3.9011082
309.12857	181.720433	265.21288
1444.84364	944.584415	915.64791
51.3374651	30.3465856	63.0597983
103.697683	57.486477	49.2038976
394.129171	545.603035	290.338476

N27 N1 | N27 N1 008 | Geometric Segment	N27 N1 | N27 N1 009 | Geometric Segment	N27 N1 | N27 N1 010 | Geometric Segment
158.875669	160.116006	99.9431126
7269.46724	5239.16912	429.680934
232.578852	242.367192	138.20272
1310.93441	921.819109	381.717989
1561.08945	1144.84776	230.169922
677.489936	615.116285	177.714276
159.307019	78.4914647	104.986577
20777.8378	9547.56772	999.761254
5133.67273	4791.65773	4740.52487
250.865496	244.105926	119.718452
19.0518888	6.40020875	15.4091253
1848.02319	1815.99496	1387.34952
181.384155	256.23278	122.043605
444.399157	437.095095	401.835912
741.67505	415.258489	255.498111
442.431906	514.762649	219.163617
110.748699	96.7315356	91.5638191
240.632537	150.39451	48.2785886
123.358647	149.186045	37.6917197
554.543365	125.423568	49.3414124
611.95144	369.761412	160.921703
312.595584	337.415614	114.209472
77.3332567	128.366815	81.7607397
198.548759	227.062493	78.9641729
1797.34565	1699.56404	1490.43733
84.1052317	83.2747885	28.6417365
97.8478092	98.6117036	59.3543177
83.6653398	87.44142	46.7830502
204.407051	87.3951483	8.34969534
205.578336	232.626839	110.847534
175.132417	154.437211	74.7579852
22.3543857	12.0154163	4.82138062
26.1153893	49.1293062	7.51006887
220.887462	216.903924	232.096578
15.0128539	33.6223527	21.5864228
160.70511	121.14061	54.9500213
9.81396829	17.5832633	7.05556951
224.515628	284.614357	121.819868
774.787099	947.601172	1222.43933
82.7680344	49.4306379	34.7109788
50.4295486	55.4435458	42.8472716
444.126501	445.796215	65.3980715

N27 N1 | N27 N1 011 | Geometric Segment	N27 N1 | N27 N1 012 | Geometric Segment	N27 N2 | N27 N2 001 | Geometric Segment
115.936173	127.423873	368.104912
918.440152	1631.86974	484.570554
189.346946	173.500786	168.726588
436.596687	551.801851	393.706011
267.737634	539.146158	429.968953
227.149411	498.42362	334.745669
95.5841077	119.768084	97.079493
1553.87667	3751.55505	1779.26227
2970.03291	4671.60069	3615.44372
138.875996	174.922522	175.717198
11.5855955	22.2258687	26.6061524
1152.84719	1300.38266	2496.19312
160.643589	166.388006	3785.99123
470.277561	491.711115	372.248381
420.950247	426.609215	870.977134
220.486591	356.681808	227.416526
80.8165306	94.7459742	87.1729362
49.0036817	65.2839767	39.7855415
39.0079624	28.7819557	169.139369
64.1055589	46.9716992	36.2467301
145.855853	83.048824	873.888567
221.877529	390.578674	374.737227
66.3909592	84.9098487	103.954007
127.418156	147.846579	188.783195
1200.54232	1893.38623	581.926575
15.074321	51.6404617	209.056513
41.6513871	71.3430759	52.794829
36.1795671	61.454182	44.8474772
18.0802109	21.6782337	66.0560454
94.7472384	151.469676	71.4296365
82.5369785	148.975226	83.3775558
17.4001516	6.95428007	24.9744977
40.6552646	32.4971993	66.3098202
299.644309	343.582082	185.514352
34.0831989	27.2439134	48.284412
55.2986001	121.936933	87.5809897
11.1401681	15.265256	6.64499608
321.488628	349.225558	249.539924
1579.91695	1872.6763	332.197504
31.3176006	57.2189081	51.3717262
60.2180197	74.8757416	41.9060033
67.681855	178.947855	46.1943499

N27 N2 | N27 N2 002 | Geometric Segment	N27 N2 | N27 N2 003 | Geometric Segment	N27 N2 | N27 N2 004 | Geometric Segment
111.82993	198.091455	344.396284
1807.06117	516.168168	1133.81424
179.702457	152.326815	279.039007
752.100723	627.621735	772.025968
867.33058	335.065048	954.648453
666.910411	273.962246	1509.844
124.69887	165.812265	199.137318
7010.64612	1573.678	5045.03595
3992.39754	3882.16132	12432.4169
163.754337	198.833384	260.798766
22.8260534	53.7058872	66.8707913
1144.7126	2022.71405	4958.97605
158.250783	1359.45594	3383.90583
428.143036	421.218903	688.139875
488.730169	455.671477	1036.95874
222.373903	159.115321	517.048922
79.6127588	110.468122	220.019145
134.093794	33.9769531	67.6349397
65.0301962	85.5973029	827.592945
336.803728	90.4832592	173.340294
186.985133	498.7228	2120.62616
378.204337	271.133596	521.293647
65.4020579	102.586738	125.613425
192.329412	194.198416	179.185091
2294.67961	911.449424	3580.49205
74.2489362	130.541567	252.982793
73.2696156	63.6521188	167.91569
55.688544	40.315641	102.14862
46.7536231	32.2354456	903.099443
140.003951	70.3858734	236.305689
159.992336	113.92743	289.846411
12.8557307	7.75573971	64.012545
25.0310622	54.3636041	64.5425191
333.85997	224.749147	588.555405
43.1685329	13.0215821	39.7792948
110.828287	81.5938764	372.640664
9.40648628	8.5122652	59.7464015
318.730145	180.039286	383.625545
1251.77734	937.610205	537.415183
46.2766819	31.9066066	65.2105514
136.218742	47.7171105	69.2053829
1684.79376	97.4649323	174.85155

N27 N2 | N27 N2 005 | Geometric Segment	N27 N2 | N27 N2 006 | Geometric Segment	N27 N2 | N27 N2 007 | Geometric Segment
443.453329	172.642277	132.115116
4924.11855	397.684599	765.508857
657.390494	183.147026	193.461429
7462.93304	338.287397	303.362475
12964.281	311.439973	260.417527
21632.2438	477.995926	334.054688
494.661466	138.385842	170.081021
62587.5774	2072.06952	1289.67098
26074.7442	6240.19869	4597.98089
1722.35902	199.437862	197.100912
107.70903	46.7125921	38.8911155
29600.4478	2576.02124	1880.74183
1125.20928	6705.39091	321.826307
1877.05586	564.062638	651.694164
4750.66266	1090.90914	420.701078
2303.32075	206.821753	281.612945
264.781084	163.217107	121.08552
824.436875	26.1620503	24.0398062
1233.5118	179.194379	145.510121
1704.44578	54.4661558	47.5699003
619.940096	750.155376	513.509346
1086.32847	404.972627	359.396966
424.76559	52.0345895	52.8543983
483.174635	150.904131	166.537868
27015.4327	1084.74425	1391.31334
663.444803	107.581858	58.7509777
1170.45346	109.079394	96.8287396
360.801916	61.1452192	58.9859913
3154.01063	225.398068	171.021596
2888.33897	105.033565	122.975198
1487.96994	120.892446	152.442788
160.590036	50.9613094	21.5059031
64.2384795	39.8863462	106.591305
672.204428	355.937398	522.068854
38.815462	15.788538	67.2654589
996.063255	186.227414	129.29127
115.514072	32.4501222	46.8929422
382.51709	342.42147	537.645452
1007.00127	205.464612	1279.82207
144.019282	57.7954062	47.7963811
168.949393	76.7484124	71.5477851
2437.04402	219.650105	83.4903218

N27 N2 | N27 N2 008 | Geometric Segment	N27 N2 | N27 N2 009 | Geometric Segment	N27 N2 | N27 N2 010 | Geometric Segment
190.655915	185.303597	225.843392
665.965747	383.514959	670.15238
264.756367	258.050189	314.806737
1640.84344	1769.78851	2377.57556
3280.37204	2421.26856	5264.87819
8132.81728	7718.90693	11203.3006
260.6917	214.197142	247.196068
4839.76889	5725.66103	14168.9949
14016.5915	18546.472	18993.5457
392.535314	403.209886	475.654251
119.418975	49.9874422	86.3039037
5574.11918	5174.14446	6873.66053
953.23368	626.740108	814.084734
932.831376	859.35402	1214.21438
1220.48169	1267.44008	1296.77675
748.464515	689.637951	1355.80669
108.703533	114.459477	133.846189
196.428337	194.11472	318.091905
210.805666	156.782433	306.48364
503.358282	629.184069	667.524066
406.431231	202.957394	414.858276
1133.95807	1112.39501	1374.30215
114.070762	89.5374403	168.990615
224.083842	164.220009	297.985129
12589.47	9843.63487	15911.9814
376.554544	287.019841	564.321351
486.155262	446.508563	648.590127
190.252215	133.752914	202.614072
389.242346	300.805168	387.272063
167.575104	164.505863	280.982756
604.319762	358.384068	623.530635
65.5259333	45.0644547	50.2758661
72.0915935	91.5847537	90.9950669
780.855369	793.421686	972.272248
33.9469829	44.3639378	42.5293821
198.829663	149.840234	298.851299
89.968004	60.9645896	73.0659509
532.69495	386.683259	493.201025
1282.7632	953.545137	1274.08428
98.5313398	101.680433	132.611263
132.562127	119.867967	156.396007
5351.17223	4773.48627	6323.13025

N27 N2 | N27 N2 011 | Geometric Segment	N27 N2 | N27 N2 012 | Geometric Segment	N317 N1 | 004 | Geometric Segment
455.408128	319.570859	311.08801
484.542454	980.668419	514.994312
228.52141	299.111687	176.356701
424.35926	615.630917	382.361575
539.03518	794.572609	410.036985
829.260752	1349.73249	513.179152
178.478432	212.166022	144.766216
2531.72226	3339.11328	1653.23407
7476.21865	4965.89025	5596.61634
231.501144	310.98174	188.742313
54.0270222	71.1649296	45.8419122
4932.97241	2877.71984	2548.18477
5819.02614	6530.61954	6071.5244
673.374248	723.226569	428.677175
676.888508	764.549262	652.08168
288.70262	336.841936	182.938418
206.193393	155.827512	129.464722
29.1903856	45.5370228	27.7613698
533.127699	268.557137	207.867437
100.995499	76.0624342	58.6017098
1903.32039	870.802235	1028.93805
538.849976	560.345456	396.962618
84.7409703	58.5840319	63.2328121
188.027818	185.005071	178.896868
1072.89313	2417.8558	945.963196
259.720502	128.110732	205.104045
132.949095	141.170544	106.517757
61.8163151	76.4148972	63.4292672
548.630671	227.206429	231.313302
194.321376	134.284723	132.384804
174.315316	298.59561	121.944466
61.0364081	25.4416603	42.9839953
97.2507272	78.4529723	26.0153677
454.157865	507.615531	302.137867
57.8814987	72.4461329	14.4677197
302.871291	155.789595	123.29621
44.5144201	49.3837602	30.4607042
366.841815	555.930433	256.509567
192.555294	762.196646	157.409616
52.4626955	73.108478	53.0727516
70.7800308	87.0332119	50.1955137
160.592744	143.226386	29.4166576

N317 N1 | N317 N1 001 | Geometric Segment	N317 N1 | N317 N1 002 | Geometric Segment	N317 N1 | N317 N1 003 | Geometric Segment
445.030122	112.294163	114.899805
732.422895	1290.65063	626.315858
206.488532	151.892696	156.915147
528.639698	458.465537	735.23851
709.05471	537.372266	1115.43803
1031.97941	714.930012	2111.37539
201.973937	137.033597	146.386198
3193.31568	2188.64322	1996.02033
11995.2456	6423.86424	9111.28356
195.946436	149.290075	162.985806
42.5048671	41.2897653	29.9157856
4396.23241	2933.82993	2293.24648
3184.72695	407.428567	259.264829
618.305543	532.025616	712.618228
674.274975	625.875626	787.639613
418.98226	330.111927	361.879197
190.704634	128.431627	118.953165
43.8848373	74.1653418	76.7319461
653.626439	98.8781258	128.962543
131.464458	187.637678	157.475994
1463.04736	671.901576	181.792724
392.801964	355.902922	388.97345
124.204541	82.5919386	88.8642971
200.120814	145.841349	137.361873
2678.14368	5378.44151	6258.3055
396.421496	68.0493618	107.530449
157.061056	210.242304	218.014867
82.6040671	68.8928772	80.904589
716.185442	171.04982	206.046967
154.531258	207.576663	201.481708
296.153738	141.444242	258.309744
46.2364649	29.8509602	32.7944244
58.4785541	70.2587957	52.7671663
474.223604	625.795967	630.22171
27.9117766	20.2102967	24.5560915
248.530522	91.5894564	114.078789
49.0604391	57.5823401	59.1481483
308.200511	298.859744	355.768166
506.904028	676.411629	999.315818
58.0769628	42.6369811	48.8148621
69.7301389	54.5670395	67.5061761
121.940802	501.856976	186.828634

N317 N1 | N317 N1 005 | Geometric Segment	N317 N1 | N317 N1 006 | Geometric Segment	N317 N1 | N317 N1 007 | Geometric Segment
108.127778	181.76936	352.247998
930.211365	1210.98178	4071.5537
163.538834	280.951408	528.393213
476.771123	747.587888	6867.87837
630.829777	912.272002	10009.0717
1114.65303	1658.13781	16878.2204
139.414754	217.94477	343.62456
2504.75561	5756.58379	43757.0932
9815.32735	11990.0198	21649.3134
168.237787	284.75924	859.249135
29.126079	51.3006877	125.109663
2587.4116	3321.12662	20549.7564
216.526138	386.776784	714.066178
536.874292	844.13985	1398.01176
517.758825	829.894674	3377.38176
353.627586	444.808945	1761.66821
125.628795	166.08364	210.673443
52.0107866	76.8387566	619.292606
183.982897	145.106365	740.527931
112.350708	238.877722	1135.39871
384.455517	303.003507	683.891333
244.717549	468.114057	698.568907
94.0637049	91.2916231	304.393286
120.368172	160.111275	417.233034
5847.19334	5441.8891	20029.0051
53.9306889	96.223469	667.279754
180.08627	176.72107	906.933354
77.36231	87.4603518	217.372636
173.382464	199.02242	1277.8532
115.958461	347.939677	1776.3564
174.162087	290.698052	1466.02472
27.499056	31.3188466	125.786763
57.612537	52.0762347	69.5747034
609.369999	645.917901	629.007198
20.2580676	50.0463272	54.2421501
129.578606	226.234725	695.400876
52.1764352	54.7707661	79.1292162
301.535392	388.693725	401.469052
911.805388	961.923974	933.043178
43.4973501	82.3953969	138.637357
62.9485416	91.0507565	153.386352
149.059958	628.75715	2130.68039

N317 N1 | N317 N1 008 | Geometric Segment	N317 N1 | N317 N1 009 | Geometric Segment	N317 N1 | N317 N1 010 | Geometric Segment
228.873445	836.089982	746.075909
443.277631	506.701907	474.061364
274.416856	313.701088	333.714884
472.155553	649.802671	595.599322
374.286059	947.616281	767.272565
371.624556	1352.32876	1435.76721
212.90493	250.417865	218.101524
1112.01121	2233.10988	2231.32588
5514.33557	7448.32675	13822.8647
271.033576	357.651921	346.212739
38.2255637	107.140496	130.184482
3660.72856	6725.36949	5630.39411
17638.9685	10004.5701	11990.6402
417.033114	497.171099	490.042099
1486.69569	1224.92651	1585.62928
260.603495	371.650989	334.051968
201.642921	229.805962	314.381293
43.6023791	29.8596574	49.867472
136.162973	733.460292	432.566747
42.2083451	171.240221	147.362666
394.550722	2471.05705	1993.14778
316.117075	957.730608	905.113854
102.250785	214.29381	200.816047
261.090001	294.942277	288.206915
906.190667	1935.49032	2035.06102
128.620377	822.727917	1070.34645
83.6966838	163.690022	153.096879
73.231771	89.87989	107.194437
54.6595371	253.641331	227.737939
98.9595178	146.52984	112.029409
141.84593	355.844916	289.606865
61.2193787	77.9195042	82.231328
60.2533359	156.974707	51.5203055
458.437227	438.096926	578.970643
39.6893257	126.962348	27.4120175
165.356735	304.619881	203.493606
29.3308923	41.9986238	50.889023
419.663919	587.521935	460.592633
141.81326	184.9665	177.599218
51.0773998	75.9502105	63.7706098
105.706901	119.574952	86.37605
213.469257	585.315163	417.903913

N317 N1 | N317 N1 011 | Geometric Segment	N317 N1 | N317 N1 012 | Geometric Segment	N317 N2 | N317 N2 001 | Geometric Segment
399.146326	392.933529	99.5681078
453.555743	494.600006	1566.22459
313.522212	282.351066	161.519988
528.615241	465.56569	277.865357
523.441995	516.491014	338.502051
561.450924	601.61376	147.831463
261.387574	245.209891	131.145801
952.908584	1988.91035	2366.42083
8127.53916	5720.59064	879.033975
360.504444	328.37487	333.552799
52.1891308	81.5984407	15.053988
5173.7784	5935.56026	773.939833
14854.2138	13791.1617	459.070124
525.127962	467.298008	161.938936
1537.78135	2552.06058	1963.40344
322.012221	294.527934	149.412902
264.065049	226.095159	69.2452102
24.8262025	33.2093446	80.546481
180.012861	421.293892	1811.13431
102.652984	131.234541	113.299156
1021.4005	2007.01497	38.4872234
603.295313	828.806485	516.519101
135.121634	168.75789	22.6800917
311.154502	313.64196	218.145941
2115.23793	1251.41539	1322.2667
276.126371	278.704066	43.4629169
82.8731852	95.9028866	42.9375485
94.8480916	89.7101233	62.2900694
51.6969283	183.764793	428.364051
83.9416877	143.268315	113.534797
243.712978	193.364129	58.2384144
53.4472792	95.3266541	62.9523484
71.2344211	61.3044077	19.7142143
515.521858	528.507979	428.465431
48.6582496	67.2405312	3.55237422
179.821686	284.893654	124.477269
41.2915972	47.8778246	33.3143915
575.602898	554.76457	180.966359
527.72213	152.051129	281.154647
69.461977	89.1631826	236.354738
112.128575	102.687704	1146.38132
563.863212	138.87497	58692.7656

N317 N2 | N317 N2 002 | Geometric Segment	N317 N2 | N317 N2 003 | Geometric Segment	N317 N2 | N317 N2 004 | Geometric Segment
202.063462	114.269471	446.348386
3485.65148	1890.97136	450.801329
221.108292	159.614332	383.330678
1004.27326	347.266672	721.233588
933.111658	407.989068	558.713338
537.725378	204.459262	712.408436
195.479523	138.972328	329.801089
3094.42714	2752.2123	1476.13276
2454.7774	1138.76438	9180.01639
612.074826	353.553344	431.609247
42.8212733	24.7745307	67.4846935
2056.27328	954.939763	5868.70443
920.583335	592.719625	14349.7727
405.174716	183.940618	494.640451
2806.29268	2614.2678	1577.82768
314.668667	209.981865	348.768926
123.567234	96.5221573	288.427772
173.112685	109.719886	42.9002832
3305.02464	1911.89696	286.937756
198.419266	102.857913	127.607356
190.045976	55.7382238	1074.01328
1170.2645	996.990887	627.63792
59.6474842	29.4480607	148.416775
272.017625	199.241755	277.132543
3573.06025	1426.38918	1028.43723
109.280847	37.2526637	300.871539
85.6481787	39.7973142	105.223696
92.6850615	62.7548802	90.0437253
924.312296	379.867635	202.336679
135.067641	113.726684	113.101975
125.093639	70.5157984	216.848167
102.964566	77.9082858	56.4411231
31.6686823	18.35399	47.3441602
726.405844	501.512179	446.043635
16.6728816	12.3471435	46.7125786
140.010952	150.815207	203.024081
53.0849628	27.6972317	55.3123152
328.894682	216.501125	367.411288
325.737784	316.50209	109.444127
244.037149	215.430569	71.6240267
1248.76679	1943.57796	104.98705
49439.4244	49084.1312	1189.98229

N317 N2 | N317 N2 005 | Geometric Segment	N317 N2 | N317 N2 006 | Geometric Segment	N317 N2 | N317 N2 007 | Geometric Segment
106.33709	838.541474	408.5101
504.202487	466.881509	535.675382
138.825672	315.449286	367.293498
291.638243	547.259234	621.622254
185.508512	1005.81699	837.456741
124.574552	1451.0651	696.494018
116.239579	259.990631	347.775475
699.685454	2083.46767	2440.27543
534.009708	10040.4149	8745.2189
212.209885	330.16095	367.299709
12.5537753	104.637418	181.86012
439.486476	5628.71116	4950.45095
265.2253	11874.7242	17671.3927
140.249627	414.472268	728.82056
1427.86469	1476.83354	2397.81733
120.785697	415.327731	427.215792
83.9733023	283.799654	271.868574
41.2928252	37.3541852	19.2681981
918.201286	556.133999	527.215864
58.7715859	156.085237	142.999929
26.3522533	1793.4511	1790.042
799.67019	884.722942	1214.65949
13.586432	234.875899	283.435323
201.045703	215.708676	312.197964
567.620433	2062.21509	1907.59263
23.3773687	965.350518	257.169784
26.2761226	178.64026	124.48998
20.2628532	107.266468	310.813943
124.845509	231.124727	205.759993
59.4790795	92.5425033	68.5780135
37.2702505	374.851904	115.336194
46.0590918	75.6877574	138.27229
46.0246205	45.5884109	34.0225878
337.913333	448.189592	445.6906
12.6722242	41.1086437	34.3316444
79.2947323	242.095951	530.735686
21.9989968	29.7468733	89.4345408
230.839876	479.643366	451.491452
241.413331	148.096521	171.251926
213.21127	84.2490772	159.736503
1402.79645	117.620377	214.878067
53062.4715	1968.87017	904.7994

N317 N2 | N317 N2 008 | Geometric Segment	N317 N2 | N317 N2 009 | Geometric Segment	N317 N2 | N317 N2 010 | Geometric Segment
389.032973	461.469849	73.1892818
1507.23961	2877.76315	208.216541
643.679061	623.939139	117.629214
979.479139	1969.51295	177.794887
750.442817	1340.95522	102.596941
638.619989	1840.86438	52.032094
497.88779	421.189369	84.8795456
2913.61625	4534.31267	196.88882
15896.5905	11674.0689	181.420721
596.014887	697.43174	114.070929
45.0438186	42.4086164	10.418679
8557.48326	6675.73516	142.340184
1935.66137	10056.3262	375.535502
775.115083	611.565393	101.498087
1289.90591	2412.22211	189.775276
700.800359	613.603876	125.54384
394.096806	370.435792	53.5619888
72.1828812	116.367687	23.7210683
831.921677	683.382709	239.477906
194.803268	198.686076	8.49828431
1211.33524	1398.93952	15.5540667
963.00807	1362.9124	39.1682028
332.486168	198.998096	8.16446531
283.530134	330.889415	116.078953
5059.47255	4455.18458	737.417097
158.136172	330.46896	16.8800861
122.480136	157.793411	15.8070625
115.137754	118.317424	19.5965178
114.455374	324.576507	38.3464006
153.106923	159.837064	38.5629342
246.151585	328.051587	36.9056562
72.2058124	89.4851035	22.6502436
54.0722635	79.7375724	21.7079014
965.915765	848.999808	166.438717
68.2043109	44.0825319	17.6386542
370.426275	297.889846	36.4190481
79.5300142	73.5096455	13.0137751
571.887973	609.232544	207.611579
1863.07883	779.32765	178.640276
181.335712	136.584976	222.327248
351.264087	420.278713	2382.01345
12195.7668	8111.1004	38211.1004

N317 N2 | N317 N2 011 | Geometric Segment	N40 N1 | N40 N1 001 | Geometric Segment	N40 N1 | N40 N1 002 | Geometric Segment
67.6210772	70.5221887	241.235098
259.469296	283.681076	488.002638
111.626332	138.848377	281.400554
216.983688	224.393152	394.586871
93.9619484	134.185983	354.708413
47.7007691	67.1336627	288.943488
89.4912519	96.1039048	245.317835
336.656945	393.239433	1344.73061
292.400943	346.364886	4737.07753
101.473052	121.012845	272.288105
23.2815216	20.8512299	54.4194074
202.638705	237.087338	2675.54814
262.114659	338.80778	2511.63373
122.315516	125.715819	305.176864
367.202206	521.348494	348.68923
158.108979	148.822334	257.905863
63.0052865	64.1713313	155.968153
37.6434495	42.287515	34.1305938
376.023185	415.235119	230.712048
9.14387942	14.7457513	39.4234357
22.1034135	26.4750289	692.338318
49.6505714	91.080036	147.700732
11.5798331	12.3079021	83.2099054
165.61205	164.540102	166.871418
727.670116	993.037039	340.310635
21.9462665	30.4190068	137.629624
27.2422868	23.5424529	55.4487436
21.9637711	30.9116274	37.8783348
59.0390706	71.9353446	78.8487602
47.1875168	57.5910628	73.0559366
36.5368008	51.4990294	198.073149
21.2027096	24.8670965	43.3988286
30.9109514	35.0710095	87.7939482
172.631498	208.705572	320.376499
15.2883346	25.7092612	59.2177405
39.8165501	51.115268	129.910398
20.4030092	19.2600772	31.2076916
215.668217	313.547382	419.668805
197.628027	216.925451	487.934421
181.848404	247.647623	45.5535417
1766.57975	2513.6321	69.2592292
36890.0824	44975.8402	43.8097536

N40 N1 | N40 N1 011 | Geometric Segment	N40 N1 | N40 N1 012 | Geometric Segment	N73 N1 | N73 N1 001 | Geometric Segment
192.427916	167.346006	196.321704
509.172943	336.867719	294.808847
322.666043	295.766363	248.279941
484.731767	394.492434	477.452281
265.880077	270.227638	329.100831
177.720086	198.284114	265.578577
232.188485	173.799035	195.141058
1236.38058	1183.0634	591.520014
5078.10158	3092.25944	2683.48585
304.158101	264.253032	262.319435
37.8336722	46.15537	54.009204
1344.38489	1902.94369	1969.59432
241.148528	2052.49094	2069.76584
494.098872	312.258486	299.660627
340.057298	390.458145	587.668356
290.785624	245.831961	285.081973
171.685245	135.590176	148.084819
29.6605053	30.309362	28.1067936
88.6401866	177.753343	122.819043
38.2052503	42.2125584	22.9168148
239.896172	513.61177	93.4348849
82.3330804	300.763626	73.4823539
50.4868488	68.7318373	65.0794454
135.059906	149.469137	165.048579
456.746009	590.13193	548.797424
37.6934618	77.1920403	143.037457
50.0098914	53.7935933	42.6860384
33.5159024	37.8542687	35.229838
52.4341705	26.1496895	47.8275418
90.008007	98.5468208	65.5825104
287.885405	169.542435	182.13749
26.9621256	27.9662673	38.6467013
134.768482	58.3011174	47.3600706
441.966072	310.660572	277.339584
162.717895	105.175547	82.7796719
150.137039	105.981926	84.3434623
37.9685824	39.382634	34.4679164
643.150978	398.211984	328.539953
1408.44562	555.502678	571.890079
53.5112281	90.7448465	117.791417
104.326454	415.158924	717.137308
71.3153266	9225.69371	16381.8423

N73 N1 | N73 N1 002 | Geometric Segment	N73 N1 | N73 N1 003 | Geometric Segment	N73 N1 | N73 N1 004 | Geometric Segment
200.146504	183.889272	190.694651
333.706117	362.341893	322.012774
277.420965	220.704981	294.597977
440.443066	374.52316	452.173774
327.586182	320.30132	309.05006
209.044014	218.475052	198.450342
156.306321	177.261811	183.643473
965.181371	968.473899	944.83703
2563.76636	2665.33535	3398.03622
247.104891	230.207696	259.790835
46.5784657	27.784412	46.4920898
1870.28975	1765.34381	2051.73916
2420.97247	3139.85393	1456.43795
301.695985	285.426459	364.782349
442.409621	403.451074	400.955169
288.808725	238.146386	275.169828
121.629422	129.418656	124.163049
31.6859893	36.2055084	27.170666
119.719424	135.335719	110.199079
18.6325905	19.6005412	19.6547446
350.740022	160.181935	270.002042
168.993186	114.428128	199.147087
64.0472679	74.4665315	65.8402349
134.571646	149.362837	163.244776
389.471327	402.264865	537.795176
120.802523	145.111769	56.4421237
47.5816523	42.3925484	50.1066902
24.5517719	38.1842091	25.5853528
30.5535511	30.2566726	29.1106697
92.7204657	77.4318564	91.286498
207.592733	185.076631	193.393919
22.6311959	22.9859449	32.3436676
47.7297408	56.4251142	45.4757195
282.587267	282.74368	349.413544
105.240286	82.1616782	116.732644
93.978361	72.5431343	77.0046269
33.0285816	22.9527398	31.4688178
329.700136	322.937085	451.422195
598.162931	445.728562	758.949537
76.9983644	74.650543	81.698749
202.540387	166.531395	227.435392
7456.64344	5918.33005	4506.09865

N73 N1 | N73 N1 005 | Geometric Segment	N73 N1 | N73 N1 006 | Geometric Segment	N73 N1 | N73 N1 007 | Geometric Segment
170.306801	199.075847	232.283192
298.313314	799.477903	653.101902
222.261822	393.487915	402.249524
433.33538	665.429653	675.732427
297.817797	559.169485	561.769142
216.417014	502.49909	454.992366
154.233102	260.862742	286.703944
807.275982	4536.09022	2833.30877
2860.42592	9769.74106	8970.63648
218.204564	385.409363	419.669405
47.1670379	16.9260783	21.9438613
1813.8616	2284.01683	2490.89603
2348.93984	324.261833	313.407145
293.789865	929.611052	907.484518
590.827858	966.739918	1141.77052
234.089672	476.516371	640.605394
132.707404	185.940616	189.406822
37.7240902	72.2470175	44.8742732
161.054495	184.436727	244.853352
27.504811	147.184901	109.947672
309.248141	595.572369	519.813009
153.844492	312.146644	283.348302
49.9765851	78.9766889	84.9183154
170.780399	167.486264	177.378631
613.687667	4583.98138	4174.96644
185.779716	86.2473037	86.6678493
47.2542461	130.943838	153.194451
26.1970316	80.2192807	72.1813059
28.1398115	198.857008	202.563994
67.5758599	212.479433	170.956517
148.532834	169.064812	167.723826
32.3510583	32.2279952	36.3461408
45.6991606	161.004851	278.272846
273.446833	1026.08723	1169.04866
81.1541411	62.3750585	71.1623598
74.3098123	222.763736	198.341724
34.3632815	75.7784818	86.3805211
356.059988	668.32705	741.605115
350.582262	1987.80503	2388.6379
110.068358	55.5946371	55.4260879
495.416921	99.4970473	101.351818
13002.233	134.516935	119.425047

N73 N1 | N73 N1 008 | Geometric Segment	N73 N1 | N73 N1 009 | Geometric Segment	N73 N1 | N73 N1 010 | Geometric Segment
274.641385	334.985251	145.297049
1414.90641	2459.03706	675.402605
532.192621	497.788052	328.642934
1620.94439	1547.54992	787.941416
1166.99327	1117.5986	873.292822
1169.59329	1168.97548	894.126765
310.732261	603.556478	228.418455
3731.80021	3905.7624	3325.89747
7779.90646	12256.7306	6288.8321
500.17592	486.594625	319.001637
46.5950246	59.1316195	31.478349
2011.90714	2352.80839	1696.32678
360.792123	416.750428	234.36681
778.288506	869.105557	535.213298
2208.64394	2223.79231	925.389323
817.981603	758.547964	526.477548
267.898672	248.868136	157.764996
96.6355955	113.11441	60.4262953
283.225571	227.128316	151.137929
155.855105	191.702798	116.350254
376.64157	300.503871	434.184227
530.56186	383.61801	345.4584
82.8212219	102.994425	69.0963971
214.448669	155.070786	157.286456
4504.54448	7066.69751	5398.66416
65.7495697	76.0710709	49.2720819
167.841025	194.921286	157.000008
79.3004154	100.467402	71.9966084
657.994973	355.909663	280.345067
282.047917	470.9534	414.238017
257.230675	264.992237	159.231106
46.2835153	62.4977351	35.8295319
177.869764	233.788059	160.603864
1129.56277	969.324531	808.549088
36.7952255	22.7507157	31.2484529
315.780777	310.623741	203.518291
82.3558568	91.3751495	65.4809496
675.619368	526.925995	517.555021
1774.31051	1888.18799	1704.67831
52.0972459	68.4506053	48.0910201
66.2046549	85.833897	57.7440779
156.689456	103.145987	165.837464

N73 N2 | 006 | Geometric Segment	N73 N2 | 007 | Geometric Segment	N73 N2 | 008 | Geometric Segment
391.215762	307.249164	321.268596
2176.73438	5903.66314	2375.9134
532.047453	486.924562	514.799518
1169.6576	1640.6315	1131.71773
1249.16091	1103.75813	954.704471
1139.33178	1034.4248	746.837228
369.480973	353.712796	413.419167
3622.30447	3098.68219	3432.40025
12389.8398	12979.032	13855.0933
537.565135	463.865717	516.153721
78.2364958	42.7073764	59.1083925
2509.99717	2977.06425	2437.94694
506.633537	566.896272	452.236339
744.770588	844.691592	655.850922
1816.57413	1884.04072	1902.8812
767.250739	1021.42584	858.564625
319.512953	334.347448	353.487827
75.397208	75.6194832	91.9407399
236.197378	411.799027	325.365377
141.540284	141.602659	148.283182
443.487314	640.178294	546.197062
262.015483	269.152314	313.428922
109.93491	143.522237	163.98401
179.526125	148.026296	190.118948
7442.65834	10207.6063	7498.65191
80.4465577	87.4073619	68.2420937
202.038285	268.392505	222.30906
85.9119395	68.2141268	84.3239629
286.610572	505.270665	351.604512
296.263707	336.542109	272.107006
273.119146	274.222926	211.142726
45.2211878	55.653754	60.7378196
175.771795	77.8094051	175.496017
975.166181	976.787127	1009.19649
31.0941838	26.1410811	33.1650946
353.902357	317.823086	326.314315
82.9689483	63.8187217	74.8723214
533.238219	541.941038	436.886209
1791.14305	1798.50109	2059.38617
51.7944865	50.8920024	71.7405538
74.3587106	88.561285	59.5834592
77.8222871	83.5697117	79.6790854

N73 N2 | 009 | Geometric Segment	N73 N2 | N73 N2 001 | Geometric Segment	N73 N2 | N73 N2 002 | Geometric Segment
260.848277	235.245601	226.981483
961.361658	1051.76916	1649.26955
458.724669	391.998956	444.476108
1221.39562	624.968642	1292.38558
1692.47933	548.615097	1829.87693
2158.67674	517.103247	2827.32178
325.867384	286.016513	257.06982
3931.52503	3307.91363	11738.3427
10995.7357	6571.15784	10517.4534
511.967552	380.417844	470.027549
55.4820805	47.0451174	12.3475136
2660.79115	2313.51331	2343.59821
400.837651	2114.10909	380.554755
769.899811	643.497824	960.894262
1397.64696	1170.73883	1872.32649
835.002455	436.003736	700.561444
290.762697	242.549379	211.620834
120.304294	27.2026793	128.831856
286.064124	139.634362	296.540218
188.704067	65.3651783	262.462101
503.426782	468.146218	325.499949
445.945253	255.23906	875.336294
152.924053	48.8167286	105.624223
200.762	214.244251	216.886782
6971.40668	2310.03391	7906.69249
79.568851	57.030483	135.092271
247.979733	127.844364	202.290313
105.447439	69.940383	118.014685
268.655321	168.931716	586.262181
336.333262	164.376223	638.357711
217.895377	122.600965	351.288169
51.3103877	29.3677644	51.3119604
195.248344	135.813371	103.546434
1095.83672	1013.99719	1206.48799
48.6412644	67.3111218	73.4105101
267.164684	227.567616	391.350331
99.2893608	83.5671447	82.0427351
614.494475	611.793426	755.588928
2225.44327	1778.44617	2153.26997
57.8505428	36.4396805	79.0987554
93.4796601	75.6061765	106.454686
369.128575	114.151666	260.778723

N73 N2 | N73 N2 003 | Geometric Segment	N73 N2 | N73 N2 004 | Geometric Segment	N73 N2 | N73 N2 005 | Geometric Segment
229.914768	276.373865	549.336936
1218.90955	581.795831	1028.12137
379.481559	174.170892	156.908286
693.519553	441.817062	287.247269
623.716306	349.108981	275.784556
677.227016	636.900987	471.084185
222.189904	142.468554	94.4237235
3873.68189	2976.33864	4028.15652
8729.83513	7219.67376	8426.62262
386.032227	160.612263	106.763514
23.5756028	40.9620022	32.0350415
2213.84527	2552.32122	3010.14766
326.396111	545.756147	310.944638
846.548809	354.593642	404.362814
1089.86938	508.356293	348.518123
516.384868	234.257678	319.041044
183.291597	124.709761	197.303828
55.683839	96.7495043	46.8995587
199.777156	113.193249	488.897973
93.9617647	79.0960779	120.817293
357.243398	364.237598	1324.92236
279.623802	511.521145	682.763967
96.6347695	68.0917101	86.0643205
159.110129	170.137315	152.323236
4573.09585	1746.3728	1065.63794
79.2412501	130.195647	328.736337
168.842496	92.6007393	104.374734
71.6959631	58.9790839	72.3832649
134.825248	44.5911653	150.385988
247.700142	90.5404225	104.263773
191.457973	123.055074	150.917657
29.7589899	19.8372416	25.1851583
175.660087	22.0385021	31.8270862
1092.05227	207.565665	276.699399
52.5621112	4.57533189	2.03307804
194.354678	69.20816	152.301014
96.7528572	11.0950198	13.1470484
603.096044	104.295964	95.3374217
2148.65989	676.043891	448.402427
55.502211	30.5148768	28.0540987
79.6864532	41.1884238	46.2374906
90.9955914	247.718874	85.058317

N73 N2 | N73 N2 010 | Geometric Segment	N73 N2 | N73 N2 011 | Geometric Segment	N73 N2 | N73 N2 012 | Geometric Segment
294.004812	408.137975	453.541835
411.135082	852.501861	820.111629
204.247338	143.460491	196.514415
345.020951	491.562548	442.011533
298.742887	335.013656	400.931341
231.316636	571.000403	454.240519
115.300744	133.64384	130.642154
1674.8492	2623.46924	2683.2002
2376.18451	7837.18959	4160.72006
179.290799	115.988215	162.381233
33.8459195	29.1079379	42.3382274
4759.53423	1739.11736	3246.038
2807.35924	159.377604	864.490688
394.288173	416.835696	535.721122
762.497124	242.151359	525.435848
255.605149	274.393592	203.902582
111.489657	138.585468	81.8902693
37.5569729	94.152009	56.8182137
182.623487	298.54153	287.006478
49.8537395	82.7557112	91.8130316
925.811488	875.886517	994.083987
426.865665	482.083768	474.772837
48.4882944	93.051025	46.430873
233.481846	134.334428	162.827335
491.809442	1606.47873	917.627307
78.6389469	197.873013	265.288226
48.2855168	126.543946	71.1273666
40.369328	78.6791228	58.9649047
58.68795	79.6858687	55.3958194
79.0225862	127.213719	115.330378
105.868902	131.165931	149.050113
22.9452002	38.7266708	18.3068977
26.8056052	16.9407066	44.4901231
159.311487	237.050164	225.07479
41.4874807	7.81554983	55.4185792
112.95994	105.085238	138.383043
7.7487279	13.4772871	11.9456104
170.579424	127.594801	211.750116
382.796502	869.620512	734.738957
50.8281066	28.7587853	37.3858987
52.4863242	47.3989231	45.5131029
513.322894	111.55816	309.14118

N9 | N9 001 | Geometric Segment	N9 | N9 002 | Geometric Segment	N9 | N9 003 | Geometric Segment
329.697757	181.428556	401.639609
376.726172	609.315542	838.110436
180.112839	197.347638	144.077666
333.483923	441.469962	304.814772
271.459723	372.793439	214.162977
270.207369	561.905648	243.675723
151.128179	111.754442	108.784811
2095.64649	1888.85618	4092.51006
2976.71801	3409.79163	6396.37251
158.049473	195.441644	97.6273701
46.8579034	9.68037874	25.1283834
5050.94948	2745.51776	2087.6849
1398.79889	3401.08654	521.687649
447.500023	460.547565	336.232138
561.900677	749.430525	487.272165
187.678513	251.641535	253.703092
103.11724	126.848939	144.495772
51.1217858	57.7383988	67.4449519
240.552212	135.981644	236.526834
54.0155676	60.8478042	45.0154888
1216.48019	450.412059	779.454826
487.942973	613.691538	420.798808
57.5395754	50.6594623	78.3211195
222.642131	238.268684	236.769238
459.429919	1059.82142	873.905299
116.64804	91.0596989	165.616815
47.2716301	62.27139	67.111306
45.7275443	35.9519136	67.3010958
71.8195445	58.5446872	72.3321792
62.7269922	40.182234	88.0075769
90.3776812	128.441486	106.156217
12.5669387	25.1145664	35.3126244
38.5383013	51.5401429	23.7650096
192.532569	183.949222	181.903413
47.4736411	20.2737949	8.77113909
119.815183	114.662802	138.99337
11.4939729	9.711258	14.1798037
193.491029	207.354754	108.53916
311.347979	792.692558	380.380237
40.3902851	33.156107	34.2922467
53.1560999	68.3079861	41.5580313
174.847053	426.871419	75.5505893

N9 | N9 004 | Geometric Segment	N9 | N9 005 | Geometric Segment	N9 | N9 006 | Geometric Segment
248.367497	367.132192	462.610191
746.481833	471.106009	515.628999
175.818413	189.485348	171.703825
370.107304	376.646987	295.840611
393.210765	318.333151	657.777632
476.359543	319.19436	814.555805
115.011339	119.858804	145.190816
2571.85684	1975.95138	2834.91566
4243.73501	3506.31412	15908.4529
147.857011	184.363091	145.619128
29.4488915	75.9068343	41.9281826
3465.27427	3533.54519	4357.5794
1365.20311	1995.63707	1290.61068
516.253446	479.44994	385.811091
467.284069	583.165691	359.197261
273.401502	285.241332	326.59545
110.840723	136.047945	235.589816
71.1371335	45.2744586	29.7236748
376.374367	274.684167	367.800174
99.957474	111.783898	109.282611
1197.6636	1152.35744	1345.88737
602.347314	639.889842	526.695721
95.1869817	36.7193369	119.555289
221.982974	197.398829	165.578127
1295.90275	628.177623	1560.36369
148.713153	169.111699	369.756445
60.890514	49.8254253	111.868656
55.526359	43.3707752	50.3093369
92.4750368	70.6256835	405.873493
107.72334	78.7703213	94.8974414
141.549155	105.872837	203.155262
28.0139092	9.37766943	60.485791
44.5617645	51.6572674	35.103957
287.792199	196.245247	536.12646
59.6195088	73.1780827	43.7046662
197.444271	101.785628	198.050316
6.64713673	2.90633902	58.5274632
285.644569	195.024256	447.337883
1100.44425	553.414071	441.163143
26.4770407	46.426296	51.4165498
70.1329102	64.5618189	45.7725594
369.340617	263.928186	367.499063

N9 | N9 007 | Geometric Segment	N9 | N9 008 | Geometric Segment	N9 | N9 009 | Geometric Segment
309.858662	271.622419	500.948881
469.690841	319.667394	413.134746
224.011013	225.046467	196.594357
318.926822	376.746667	341.137004
521.379923	507.0094	610.119859
662.299625	823.957345	835.599362
175.708485	150.054716	130.777591
1698.37431	2397.41794	2208.22399
9031.3307	14486.2712	12643.5796
204.302791	188.332032	169.224002
40.5822446	27.1048063	60.6535503
3219.95685	4741.25235	4047.83432
1698.01265	1204.41358	815.361307
410.789882	298.531411	307.6093
504.877639	618.861217	385.854785
252.526214	305.106678	270.740876
134.776065	203.868386	168.826097
35.8077055	29.6145594	28.8803563
190.205527	127.068423	228.138786
72.8283624	70.4376964	101.412592
784.021028	548.233318	949.054437
316.173662	304.805682	325.517909
57.5425525	96.5397322	76.9251184
178.735825	165.690107	165.42455
815.723799	1385.16719	951.859834
299.324882	319.907439	525.214986
97.5338816	91.519383	95.4249963
41.6200481	67.1510538	58.8140635
117.983357	89.3396695	154.776068
119.1608	86.1299937	127.745962
211.661655	200.294066	213.718173
34.3373613	61.5606115	48.2764028
46.7881153	35.2157362	45.5816816
469.916346	424.32493	401.013837
81.9836251	80.7956934	70.7646832
154.814996	120.507932	130.118217
48.3649638	40.2487134	38.5565727
367.232173	291.979895	370.909659
527.368573	598.117381	559.19144
42.9592703	50.0104996	56.8851387
64.1363032	48.7198228	48.0437563
141.39658	398.647021	242.306866

N9 | N9 010 | Geometric Segment	N9 | N9 011 | Geometric Segment	N9 | N9 012 | Geometric Segment
421.234103	355.339208	224.13585
420.129917	495.597107	459.881249
133.504614	191.174074	197.302506
287.527962	287.255239	341.639503
479.651574	430.471746	324.611318
688.493101	498.577194	349.354493
111.411256	128.934715	151.273114
2524.91152	2827.88059	1662.93557
13701.2087	10317.2947	7959.34678
135.974887	182.80338	198.04407
50.9523827	54.5055859	44.859042
6040.95477	4044.0721	2922.34665
1328.96411	2049.45715	1716.93677
270.631108	268.062309	316.08704
477.100011	583.193524	389.632322
302.696974	246.390232	245.581427
217.932314	164.519621	170.022638
36.8185051	30.0371171	30.8661532
324.782822	307.880441	174.036359
73.9264909	70.8429137	56.174373
1346.13743	1412.37188	728.185573
561.928368	450.384889	376.671467
115.443836	70.0881132	48.2294012
173.147219	198.835752	185.281283
1273.622	868.723744	665.339995
297.242043	326.216537	144.680406
110.189374	85.3705198	59.7234884
67.7375129	74.0213224	33.9946787
198.203285	196.218818	110.169518
90.7637794	122.726813	100.432987
110.129971	136.288465	177.573871
53.8309753	51.6022399	45.5397656
45.7165499	43.2273833	64.548504
453.183512	372.601668	414.768182
28.4607774	33.5548402	48.400898
190.433876	195.622267	138.958493
49.0132898	42.6748554	44.5715947
329.446867	315.782957	381.335628
242.313665	179.943986	538.891134
49.5464189	55.6328601	54.6625931
55.6305916	36.2844863	56.9571575
360.589641	226.88874	114.073227

N94 | N94 001 | Geometric Segment	N94 | N94 002 | Geometric Segment	N94 | N94 003 | Geometric Segment
345.441504	331.346414	231.924713
352.587667	352.718712	470.108968
177.803363	149.743309	242.930453
297.842298	296.754539	402.02565
490.232429	427.397421	448.632458
632.735139	574.403075	460.530433
120.662581	129.917289	178.93836
1882.22585	2463.31603	1728.22506
9906.80924	9183.05159	5662.53768
180.104784	155.260795	242.659284
56.3292111	40.1269136	43.5964199
3287.85405	3596.70526	2280.18332
2306.36777	2648.55466	2935.95843
277.444241	296.948521	425.068276
320.477415	560.853196	472.450457
264.297041	281.219993	210.31172
161.531761	173.063269	117.282782
23.2842	31.4633988	35.3319582
201.472742	295.317272	166.058812
77.3817417	73.7091555	88.3926688
817.446813	1287.25691	599.930786
344.394117	509.284042	527.783574
76.8725397	64.3895964	46.5373695
144.586866	152.552392	158.757051
725.807481	1121.23756	695.020172
302.742557	236.53357	166.125143
82.4180893	92.0192937	67.8139041
39.5823981	56.394167	47.2048195
148.532568	253.670977	105.027307
93.022545	91.6925038	95.3015503
149.774792	121.619347	223.642646
35.0138631	54.4251811	32.610873
43.5127395	37.3931075	92.6144638
394.879087	392.95016	466.813682
38.6015374	25.5173976	168.568517
119.312743	169.68222	143.132287
45.784566	40.2202925	45.6140267
327.52609	293.45472	506.233273
303.516628	208.037042	853.663862
43.959681	47.114653	60.3246364
37.1037567	31.7845806	52.8318861
130.067154	144.569056	195.267919

N94 | N94 004 | Geometric Segment	N94 | N94 005 | Geometric Segment	N94 | N94 006 | Geometric Segment
263.306248	196.032699	269.603905
372.252415	452.980401	395.2747
207.281139	202.319716	151.513334
307.272277	278.489321	369.338883
418.375348	360.964994	406.863203
534.590643	365.104047	500.397253
141.218959	141.893114	135.636223
1680.37642	1592.59294	2066.24679
7359.54269	6809.48031	8645.60673
217.922668	194.369683	168.805257
37.8471398	20.5704511	31.850355
3378.62447	3146.33709	2462.77314
4254.57097	4763.70321	2236.359
391.272255	343.253949	361.217644
502.364197	644.160521	355.592018
223.120937	186.638065	207.303722
131.57832	136.659058	135.839855
42.5536229	35.3351941	29.0136288
189.300745	139.915842	161.440084
51.9261131	63.9059288	66.1632977
948.586185	609.025653	836.14344
309.582492	305.461018	335.136214
62.7889339	51.8872985	62.610114
197.357415	210.506822	163.210125
788.237566	678.490313	793.706211
288.755514	174.986065	242.893265
80.0109622	69.3272285	73.7984096
37.6681921	51.5579432	41.511592
156.438661	124.636031	98.3288058
110.170294	83.9171033	71.7085509
151.87073	127.746467	148.001835
42.2081022	42.6102971	42.7760117
36.0010925	35.9634882	49.6669005
364.378846	372.805731	454.791615
52.9994401	38.4856672	55.7522639
151.276442	109.447852	121.096966
41.1503468	34.3245728	39.0438271
316.967035	377.303946	387.794168
198.356163	287.588164	570.743476
43.9055931	47.4391012	45.6296181
46.9226154	48.367947	51.9128985
146.804313	141.501855	95.7926854

N94 | N94 007 | Geometric Segment	N94 | N94 008 | Geometric Segment	N94 | N94 009 | Geometric Segment
393.813545	362.018835	398.953573
437.991895	440.14838	465.695375
202.938324	156.833052	222.35402
400.050573	325.678518	333.937248
606.713237	619.379881	619.872452
792.519637	733.833132	800.01074
155.056713	128.737483	185.668689
1986.10317	2757.5174	2064.31844
16335.7154	9444.60578	9791.94294
196.651805	175.384276	245.428647
25.1124848	33.6656501	49.0010115
3871.20876	4607.74557	2535.64529
1210.02759	2422.9575	2406.029
489.353873	319.276864	476.096679
475.737172	765.673997	391.062817
325.701401	257.56639	231.982735
203.49616	178.907705	146.958457
52.9151358	40.3889718	23.0608784
347.389155	439.436199	176.74246
113.165132	109.044754	54.2191704
1517.77874	1712.95608	817.204712
355.779485	538.955441	336.578203
101.203852	82.2816462	44.6603093
154.459516	189.036363	196.574361
1611.08984	1292.97075	800.42183
362.926101	347.283292	420.472736
112.564475	112.110383	84.269125
68.0782107	77.3080193	40.2434429
276.687284	387.136703	144.368919
96.4928339	101.862795	108.810636
206.338307	157.759914	254.603527
60.8620105	64.1553813	30.6283512
40.4914199	25.4363569	35.6935172
484.664744	431.983067	441.931147
65.1503818	47.4392567	88.7047937
172.352157	205.438899	138.207139
38.8683913	46.053935	48.7985394
370.909193	323.064688	389.00849
812.233008	200.473114	585.448152
60.230388	40.0518382	49.0204362
46.0572631	35.8100687	43.4038962
478.545157	275.149221	125.436784

N94 | N94 010 | Geometric Segment	N95 N1 | N95 N1 001 | Geometric Segment	N95 N1 | N95 N1 002 | Geometric Segment
228.152261	173.578672	137.781234
1479.36113	865.516992	513.429475
407.956745	316.032562	242.260273
2271.01858	1579.02584	1498.25958
4351.66199	3580.43464	2272.67237
4514.73285	3907.30134	3487.70424
284.444117	180.013315	258.710736
17263.3875	12313.6811	5495.7704
9834.63322	8966.09038	9619.45714
504.405451	377.567964	281.952208
59.8646447	54.4108188	38.4595896
2622.34943	2915.31856	1202.67694
812.385642	537.663076	498.122094
795.002144	522.752923	487.518425
779.114029	489.64813	351.763694
527.180199	470.319555	355.108381
172.374805	118.265374	96.1117921
150.555622	167.911745	101.671692
149.457414	109.972249	64.3136163
155.729107	141.09323	175.021229
97.4691537	214.440937	126.625079
1064.09337	1031.62846	1034.87654
47.7145257	70.0704368	32.1632531
226.617235	200.897842	132.330778
5697.36758	4436.35943	3034.9106
212.380216	210.599182	155.641234
180.903206	211.586498	230.735925
93.5276811	77.3444559	85.3907287
248.023874	151.649222	115.57719
382.281068	437.61865	189.361856
737.445893	528.207649	318.037175
52.7032226	47.4295496	25.1050526
120.763487	117.491308	75.2849126
708.465786	682.695953	567.823997
220.821015	160.730429	58.4375313
236.944742	197.722632	112.644995
53.2632083	46.9834678	49.8978741
747.225863	543.03144	364.02876
2166.26717	1828.41334	2244.51982
73.2515701	65.7135089	55.5119221
93.1634383	68.7263537	65.8357591
885.926562	1466.20843	1482.47939

N95 N1 | N95 N1 003 | Geometric Segment	N95 N1 | N95 N1 004 | Geometric Segment	N95 N1 | N95 N1 005 | Geometric Segment
104.081933	129.928289	133.585276
624.326548	650.238349	1583.3466
237.152923	333.395796	325.517801
481.34046	527.447244	1031.15478
283.8572	467.09326	1539.8296
228.465227	301.342671	1369.73198
174.417278	211.908147	208.495257
994.101019	3133.36747	11904.8647
5345.8123	6909.56865	6500.68867
268.056435	320.299742	325.643736
18.3566774	26.9003773	41.2845145
1192.74024	1514.69458	1976.70869
210.548601	220.916093	311.846037
413.871949	510.868479	627.751046
329.535077	380.124434	776.394632
277.390148	388.753278	592.464118
118.119872	157.534431	167.858187
34.4163078	67.3471024	245.731879
81.4441507	123.909012	220.989017
31.557981	39.3946802	244.133255
236.797112	319.416363	1053.22357
121.058219	194.80344	1502.60791
63.8531319	76.2934642	84.2591511
109.932038	175.965727	283.342427
559.044479	966.103345	2273.49837
24.8296951	54.2422635	101.297546
50.0811855	67.5191125	129.528563
33.9575851	53.234168	90.4529933
46.6853098	49.5987518	820.262433
143.459215	180.461582	1107.00055
246.09871	233.536471	423.786575
13.350164	24.9980556	41.9274486
105.937204	108.665838	122.887222
540.825767	622.631069	928.663281
97.7840606	110.687405	166.868926
122.412477	172.94866	264.713344
43.1744419	52.9975583	41.2097601
627.486965	423.874667	871.007942
1900.07144	1924.70476	1736.01291
53.0149461	50.5374464	87.2558646
62.2393127	58.2712615	93.9014998
73.0956491	99.0366746	1119.8233

N95 N1 | N95 N1 006 | Geometric Segment	N95 N1 | N95 N1 007 | Geometric Segment	N95 N1 | N95 N1 008 | Geometric Segment
211.794722	224.820414	142.47316
4555.40137	2665.12193	567.027592
489.047837	428.764076	280.122248
1755.56942	992.004472	437.702835
2438.82583	1217.77682	289.374298
2412.39611	1021.48406	222.414237
291.40438	236.455746	187.150754
17593.422	15485.3634	1145.64693
7935.03509	6004.31611	5775.60875
527.49831	452.754715	283.927575
43.6367568	32.6594753	16.6332696
2477.50516	1584.87063	1477.54711
497.96508	522.957088	284.569501
753.915582	526.58779	508.407727
814.521877	1184.46714	328.172308
698.709136	484.715445	299.659791
185.307567	173.883638	134.097109
188.896576	152.995903	45.3427736
216.185903	264.796322	117.803421
229.157665	177.519206	43.3393793
521.55378	413.648049	304.965749
1047.11785	976.162549	158.802722
81.9351382	48.7581157	59.7356827
358.344824	209.892505	131.223878
2937.04165	2139.20659	610.481439
171.714617	80.7932763	39.6468437
150.599495	109.012381	45.0954783
84.8123754	63.6379135	30.3305856
316.15364	540.246038	57.0548241
1310.13748	745.667449	123.808999
561.002716	389.665689	249.579884
49.530008	57.1385665	38.8621123
126.681759	100.256379	170.720466
807.79434	715.297654	546.445032
202.266644	181.059101	137.414384
294.619369	271.629922	147.113917
64.6340705	52.2124836	33.5624127
1016.95428	902.521777	593.037053
2305.76308	1383.89322	1684.32407
112.931496	88.8844656	65.0868382
143.813641	75.2610579	57.4230309
948.64205	255.408872	62.5923316

N95 N1 | N95 N1 009 | Geometric Segment	N95 N1 | N95 N1 010 | Geometric Segment	N95 N1 | N95 N1 011 | Geometric Segment
146.556018	206.307151	88.7305045
594.063556	1043.04446	1219.28407
311.416771	134.77174	135.930998
582.832478	277.322177	318.58958
485.184231	365.16813	272.430343
386.509777	585.858785	375.83627
206.960963	94.3845652	105.602515
3593.05048	1541.6678	1410.79048
5785.37946	4855.9493	4346.02903
346.253058	122.193074	132.876748
32.8676602	34.7260402	11.3985844
1269.20736	1074.64581	615.432403
323.182574	1066.92326	143.49834
513.133619	469.057977	432.79105
357.88027	282.420451	232.425914
369.605912	215.6732	214.510928
132.882876	112.941319	97.2173422
68.0986823	50.5070779	50.0524327
109.60239	253.114381	87.8606262
55.0918769	60.81779	36.2964042
290.811869	732.371267	232.227313
456.855802	269.815942	165.558512
53.3593262	64.3724691	47.515419
167.238298	165.185229	91.9398176
907.100692	1418.36151	1631.27437
45.1654413	107.623991	43.4975682
69.227566	98.0831763	84.5891548
38.1256428	61.8156085	38.7696399
90.0892336	93.3896417	55.9376207
270.420952	142.445726	106.106527
237.644473	128.525593	94.4482214
27.3727323	25.5285011	15.1459343
149.45546	31.6906495	26.8921501
588.875162	596.556384	556.216073
90.1269815	17.7126027	12.0244925
141.70319	147.033141	91.7766167
58.8094744	41.3927239	36.3745166
506.703599	324.949969	289.293522
1473.30766	814.126732	1009.28147
62.5826235	34.1571609	32.8743421
64.4082735	57.4262777	50.6994128
156.912093	61.4704426	63.2772021

N95 N1 | N95 N1 012 | Geometric Segment	N95 N2 | N95 N2002 | Geometric Segment	N95 N2 | N95 N2 001 | Geometric Segment
229.413798	93.6092268	91.8167805
2894.57649	1289.56717	609.05916
156.366022	135.831347	180.299301
387.048615	347.040832	373.082527
452.533981	395.902021	347.412242
679.334185	611.044917	473.51236
128.343748	85.8870661	139.788709
2041.85865	2140.98356	916.827918
5165.96429	4992.71486	5014.71314
174.863892	119.242	187.412057
32.0368514	19.0700031	15.9087045
1174.42734	853.536656	983.347615
896.302273	135.794144	308.899569
574.373366	475.800379	561.875408
346.304422	222.347999	319.627287
219.477722	218.632271	241.888252
120.804548	94.2046066	101.988905
49.3084277	72.3897058	46.1308073
180.050458	78.5186899	87.475856
77.801528	53.9895386	48.1250398
546.247327	232.063392	226.502893
374.459651	308.808162	317.333413
64.3364121	58.1408747	64.5613082
140.488788	128.297109	136.003698
1585.08306	2174.54089	1821.21499
109.799972	82.2584467	43.9729456
97.8102725	105.237627	73.3309607
54.5558935	54.1959635	46.8450294
148.929533	46.7294318	27.7373548
167.84663	130.302687	82.5073351
128.669686	161.664597	217.717817
23.1092487	20.1740414	15.0918708
36.0613123	55.5317133	43.4976797
626.090058	714.312755	575.847412
19.9635057	53.1092935	31.9762139
143.213079	114.745415	109.232939
42.8444791	42.7630524	37.4177621
407.050761	433.92423	476.853725
877.399912	1208.09698	1185.94544
49.106281	44.4483885	43.4531595
70.1033186	71.1981325	72.479694
48.4173241	112.64316	43.0695484

N95 N2 | N95 N2 003 | Geometric Segment	N95 N2 | N95 N2 004 | Geometric Segment	N95 N2 | N95 N2 005 | Geometric Segment
234.346636	81.8200418	185.247507
1542.77974	1282.38945	990.996898
153.007621	119.752199	185.92536
358.001046	372.013971	422.794105
419.225228	365.537151	355.944711
457.613133	504.563949	490.74019
108.34783	97.5130852	120.173627
1427.47559	2413.05566	2043.89269
5452.26116	4616.34048	4198.3221
165.940563	147.539293	192.770256
18.8617709	17.2186766	24.2415342
1967.59944	690.460302	1036.67113
2680.2137	156.873192	1369.34564
668.797174	478.918213	520.455196
396.905392	241.723754	315.837881
241.88608	236.849389	234.868957
130.030693	91.1949934	96.4298444
48.616779	52.342592	43.2491901
219.194709	124.086931	103.27621
46.3807152	60.2087437	37.383025
641.486557	282.608472	390.958416
303.445885	218.978243	205.489905
99.9563938	68.5046748	63.6341381
237.714245	127.747155	141.4099
1491.96449	2026.59379	1183.97114
120.962122	54.7930067	65.8049768
101.392311	107.208018	78.9145427
61.2762497	53.4823038	46.0683928
87.3166933	62.4996961	53.4479615
133.217411	160.197879	113.565985
123.667376	100.867636	152.037314
25.3304496	22.4643313	14.8931986
61.7698199	25.6550434	51.2276235
562.837917	655.766396	569.500333
57.3237215	10.0172225	34.4448097
163.066123	109.944003	109.899887
45.3990267	30.5138048	39.0972574
419.098348	328.394347	330.959224
762.469219	1234.95013	1189.58885
44.0987288	35.3375093	41.367682
79.492808	54.9110192	65.2073478
29.9451545	38.7000993	39.3773835

N95 N2 | N95 N2 006 | Geometric Segment	N95 N2 | N95 N2 007 | Geometric Segment	N97 | N97 001 | Geometric Segment
94.5807124	76.5466386	156.147749
1325.43286	1894.36217	861.087197
117.189144	117.345711	242.917125
404.374263	339.630045	400.53662
638.251136	348.379535	417.214165
767.650787	456.901166	339.849093
104.258281	98.6652822	128.408335
4052.01909	2248.32012	4695.55807
4968.19428	4221.65692	10414.3577
143.596908	101.915719	221.723677
17.5196374	16.1384988	24.1894528
1196.23172	687.149069	2645.71224
185.734437	99.6756224	192.630138
496.366683	476.603022	383.029914
505.130069	203.981472	455.975971
220.472623	196.786396	324.278753
87.324431	93.6205741	171.921046
112.631761	65.3877938	54.3725252
189.488478	110.991282	340.908126
255.335622	50.1049267	110.298385
384.431245	200.290273	841.183016
566.755608	249.088725	333.131364
64.502056	54.3531585	68.0892458
135.423622	108.137247	111.621309
2163.40143	1734.08242	1596.71596
80.8147181	46.1202551	84.2688922
103.215452	103.319714	54.6512588
66.1081173	54.7897078	70.5747253
354.956927	59.5648757	187.283638
147.673344	149.424386	154.733142
127.23803	101.091365	140.673597
28.7892162	23.2483155	45.2131544
33.475403	24.3066864	72.1589124
735.381282	619.014004	563.875603
20.1064199	13.7109479	53.9039864
137.369818	120.863697	153.435316
49.0112316	41.1480138	38.4242552
372.306205	300.425939	476.016556
892.829657	1070.68239	1184.69825
41.0093016	29.7137388	45.3275412
64.2715202	53.8364488	62.9981984
535.927706	62.8067744	338.293842

N97 | N97 002 | Geometric Segment	N97 | N97 003 | Geometric Segment	N97 | N97 004 | Geometric Segment
231.75481	138.279296	168.496963
945.887675	1582.99486	672.659062
191.795202	233.914504	283.619937
436.742795	660.35882	448.997807
901.438476	1062.42519	672.105807
568.431328	1079.93294	579.89991
118.282259	175.680177	172.300986
4618.68914	8634.55688	2733.88289
6834.97364	7890.46891	4289.29605
188.864204	277.164772	294.274337
71.0587001	26.2096903	29.2757646
2881.41289	2148.35869	1605.02791
577.373849	179.31801	419.573627
422.107704	524.438156	464.303737
395.617364	751.242404	409.375297
482.973738	395.455437	354.811859
153.383108	155.580391	131.985242
73.5287414	150.939912	63.8325361
460.903484	270.941056	114.904733
80.7061508	273.494512	75.9470671
964.232345	605.88892	207.385282
432.994271	676.049838	522.749579
131.464486	95.5058381	55.9924085
190.712718	132.647839	166.124742
1298.7448	1755.09001	1085.66587
176.744422	108.927412	82.9568251
76.5011737	92.2776179	79.2209982
80.1007636	75.8316939	54.4517182
233.191275	374.812002	49.8842797
208.613751	360.587487	138.130124
240.507824	210.986558	329.399991
51.3159375	56.3886501	36.9982603
53.6737775	60.0746799	73.2025793
519.505391	574.036471	519.144239
55.7084731	48.4341619	129.102535
277.979074	245.472148	163.704435
36.1457937	33.4754596	49.5315995
473.704666	541.794686	738.929477
1232.81715	1328.89761	1570.22713
48.4195426	55.2523213	67.3264083
66.5130902	78.7102404	56.1761503
594.181033	821.664431	597.174213

N97 | N97 005 | Geometric Segment	N97 | N97 006 | Geometric Segment	N97 | N97 007 | Geometric Segment
170.60716	125.193038	252.853872
886.553111	814.707876	472.557926
250.25572	229.277205	184.788637
410.295774	343.658858	352.886041
433.153864	391.843829	502.282271
402.945644	325.23952	496.053585
159.877954	153.362264	167.882475
1742.06553	2495.48099	1498.39967
3952.5251	8851.73364	8184.34201
257.931654	210.032088	195.906198
31.2940336	14.4149947	41.3265201
1650.4061	2351.9875	2387.14895
3437.0429	184.97793	1227.53774
397.47273	442.011672	358.562293
373.274804	407.448906	280.948137
277.193573	314.631744	220.593769
109.340329	149.97177	151.182976
27.5631072	51.3866744	27.5695424
196.637341	230.601033	194.02987
56.6462439	101.06611	35.0908226
604.207718	619.804202	634.994205
278.11701	316.938506	146.178766
30.121857	91.4838728	57.7708438
149.580937	130.944182	129.489168
576.893945	1319.76525	399.842112
116.51023	56.4524144	349.136991
48.8290152	47.9122651	80.9954884
42.037389	48.1228413	33.176079
57.5694127	140.40428	90.2252434
157.261955	158.947124	99.9606934
287.340822	121.33981	135.417001
29.7793934	37.4358551	30.3774414
122.162425	49.5604592	39.6103624
439.079362	487.22973	341.662926
143.369632	48.153615	48.0411673
144.564954	183.563991	138.759984
33.3077241	46.1288471	42.1669799
594.471612	428.149323	340.284826
1257.70858	1235.25425	493.947401
52.5321143	57.1399617	47.9855535
71.1555409	49.0932904	39.8398129
169.319444	319.739064	125.545157

N97 | N97 008 | Geometric Segment	N97 | N97 009 | Geometric Segment	N97 | N97 011 | Geometric Segment
169.1991	238.469287	180.956521
430.704368	360.717198	19065.345
147.948986	178.33798	409.439755
235.862348	270.985647	736.025737
373.248655	466.773119	1186.40335
354.494554	484.501972	1076.75995
110.307256	109.295645	229.583064
1009.24776	1381.84436	70413.2608
4004.47551	6366.24191	6163.45916
192.369285	170.661852	416.649311
34.3610163	49.4045088	44.2217275
1526.06637	1665.08566	1431.90127
2962.00123	2773.54277	293.915071
320.331449	303.663867	476.481923
303.639513	294.180308	523.059723
194.15262	212.098499	468.295232
99.5187814	119.422565	203.256881
28.7829631	23.9293709	137.675297
84.8439478	132.952366	234.800427
33.7043764	42.0840206	83.6072385
323.46281	457.597725	132.231511
149.555661	193.254445	507.35734
36.9397476	31.2208437	66.7400486
122.960929	164.03159	167.400484
316.962408	483.946678	1132.83258
129.822095	241.366119	93.0133415
48.9904238	83.7741201	81.8167067
34.8600421	31.2818228	54.8337297
48.5742064	74.4309016	116.804837
91.6126836	90.4068875	760.24735
168.16283	180.077212	240.125365
22.7463883	34.4445388	27.8460054
66.2284552	36.1401177	54.6971507
363.454337	353.965919	506.042422
68.3078025	53.4184548	89.6921143
69.5816298	134.102808	196.572587
28.8776779	36.824476	41.7209634
318.319653	313.377364	497.464132
752.751568	480.433154	1200.49015
33.1693606	44.3187357	66.696325
48.2444241	56.2800692	58.8586463
126.262215	161.589161	343.966334

N98 | N98 001 | Geometric Segment	N98 | N98 002 | Geometric Segment	N98 | N98 003 | Geometric Segment
345.505247	250.249332	239.660877
848.394573	1461.91752	1692.00422
489.597865	344.666248	355.795837
5516.16575	934.18518	602.201555
14041.2907	1795.83926	1277.59834
29586.47	2254.08534	1525.78648
398.808788	221.332039	251.430343
5468.44524	4525.36318	4781.10843
25259.5702	11178.2373	9882.58381
833.462121	358.4473	309.888723
84.2832455	24.0115548	36.6596314
3332.06213	5883.6691	3200.7027
986.180385	542.382454	1082.02739
1038.51492	828.204517	692.379229
1662.15151	911.521079	1056.04176
928.890696	768.695228	542.785082
149.2618	217.140628	180.828745
152.576185	71.64991	61.2592237
246.275449	355.104153	456.300644
715.092156	184.144843	154.232088
225.810963	645.941336	984.732887
2317.70236	739.537167	587.275778
95.8512023	180.561655	139.692596
296.662391	354.745267	384.56702
22597.4214	4496.75088	3239.1916
606.411543	208.146158	138.670718
657.701529	141.157399	198.096889
184.257513	92.3989978	124.900038
367.31929	124.369234	183.080543
312.96278	165.583491	139.219276
972.696261	585.449161	380.285852
102.812114	65.6178044	72.0676522
101.448938	127.860982	178.57434
1063.23475	1042.69452	1001.50383
55.1604227	148.876284	137.75564
264.619343	232.037533	393.806972
132.194585	70.3558521	56.2927557
850.25087	1379.71782	1337.89675
2882.21421	3420.30662	3209.32979
98.0179309	160.814888	116.273553
164.676809	152.156671	190.067838
2664.99149	2257.36127	1099.88296

N98 | N98 004 | Geometric Segment	N98 | N98 005 | Geometric Segment	N98 | N98 006 | Geometric Segment
474.453033	417.077349	284.808338
11295.0916	3012.50851	1897.90527
826.107196	491.843581	381.303178
6368.9535	1726.4575	897.908289
5945.23899	4926.43202	1454.69737
8692.57405	8097.79733	1837.84356
545.810006	312.991148	331.301939
51621.8869	8829.81289	3110.32354
17174.5975	15056.558	11363.9983
842.940262	422.40771	357.546236
94.5953614	52.549927	59.3402955
5957.42071	7310.77364	6540.49261
997.153393	1239.97773	1893.49108
1742.83414	1097.03987	809.807788
17590.0067	1824.06319	1161.38906
1954.76464	1282.06305	666.620111
327.47395	319.858301	341.030224
489.033321	141.85069	74.4090089
1283.3202	887.557173	583.90449
963.101036	303.056155	96.6942315
407.312835	2416.65985	1581.76714
1449.24848	616.813843	419.270834
201.709999	264.005572	188.886908
611.426456	453.248894	499.566615
15080.2685	14907.7946	4235.88501
343.378245	273.320045	108.869177
394.676809	328.993761	139.164483
270.042206	156.228365	95.3855482
3624.60626	675.070503	320.549047
2140.45407	352.734604	226.734657
1020.59168	768.762648	353.917601
132.765501	80.6884965	76.9458259
123.961887	131.841644	218.251653
1612.82833	1130.73027	1132.9349
42.7663206	129.49245	151.533219
723.927044	443.044796	482.78582
171.19464	92.6640825	151.143611
1149.91928	1320.85621	1406.52582
2118.46027	3720.2382	2447.65178
193.377824	141.030907	184.968045
192.005842	172.903288	161.010859
1133.32948	1529.79112	667.911008

N98 | N98 007 | Geometric Segment	N98 | N98 008 | Geometric Segment	N98 | N98 009 | Geometric Segment
299.03191	411.959758	291.729176
1294.24097	1773.40996	1365.65291
273.406877	392.867291	375.355563
675.284837	1504.90134	1117.23054
1380.19324	2641.3605	1943.25942
1354.94785	3840.18719	2526.47269
162.774764	257.483338	205.71545
5455.5661	6787.4317	4838.60272
9800.47637	12681.1636	10151.3336
268.670345	334.640167	374.916195
57.8535292	36.4175067	51.3214848
4113.33762	6540.88331	5616.58218
629.674885	2142.31215	303.462225
560.228469	645.330516	706.311718
948.695404	1203.03542	904.419529
568.912175	723.172277	699.604683
207.114043	231.40713	241.20985
98.6842843	125.24568	146.207597
411.903167	544.362109	448.77924
110.351331	244.820959	184.526305
1009.86436	1372.40654	646.305742
296.504798	358.909579	237.746697
129.262196	162.611493	159.958744
452.44458	416.844071	415.568064
3089.79259	6758.3782	6523.56293
169.67326	230.600019	159.626213
136.982321	157.764054	168.941155
91.5156336	97.340216	94.585756
288.177394	409.292994	181.933768
116.903472	224.183634	204.266046
336.497697	589.39349	468.207059
46.7723278	42.527532	30.6470746
142.073823	106.260963	68.267741
879.57068	755.950145	728.553563
112.30653	138.279667	103.431558
315.370301	417.684689	380.575507
70.27782	61.8385129	67.5382408
1156.12491	1084.0515	902.733565
2265.72393	1815.52143	2038.20658
120.722343	136.014708	90.7953873
100.517334	143.323884	100.989815
1472.16346	1374.73536	1381.31787

N98 | N98 010 | Geometric Segment	N98 | N98 011 | Geometric Segment	N98 | N98 012 | Geometric Segment
200.794536	303.992757	231.300014
855.34248	1225.61285	980.583664
317.651515	415.633077	319.031405
670.87412	4107.63613	576.301376
1113.23178	12245.4233	990.287101
1186.54673	18230.8414	867.02937
210.254185	295.997965	251.081972
4358.10348	12606.0383	3921.56512
5869.94193	18825.5985	6197.22204
336.627073	690.362049	275.926879
23.7790557	80.266292	29.8592127
4076.85905	11431.4056	3346.72197
500.363065	630.868744	2077.79824
424.64423	1034.48582	411.988278
715.644301	1985.77991	754.24152
552.915388	1363.63992	489.15181
184.260898	263.388956	209.930435
55.5730699	228.420021	51.5785609
215.274157	932.968825	287.824601
84.6304362	401.249772	57.9447496
532.870164	1173.19632	717.880962
174.954342	1958.03131	107.191291
149.156657	232.502028	115.707284
278.123957	569.06236	260.468867
2540.25228	14863.9583	1651.233
110.028464	554.070391	80.2369722
90.415522	520.483589	114.552805
79.2376261	171.911605	53.3936116
119.006594	570.602769	213.541652
58.7904212	285.222875	110.369413
251.759227	1474.47147	257.752136
25.3927116	85.8936422	33.4906885
54.7387842	145.482828	56.1520574
431.801823	1251.00337	480.18436
57.3611109	239.694906	72.349186
228.121592	502.838737	226.847736
48.4534471	134.11493	51.5840221
396.030081	1358.64982	441.340326
1233.07753	3534.93358	1279.65455
70.9393904	212.057937	56.6533445
53.134816	204.36977	66.6426555
1402.21932	4948.06365	919.85123

N98 | N98 013 | Geometric Segment	N98 | N98 014 | Geometric Segment	N98 | N98 015 | Geometric Segment
183.956822	318.949077	352.01105
3475.09267	5952.4851	1810.08719
332.20494	570.336914	299.681124
3943.40107	1291.69482	1142.00302
4724.54312	1112.54588	1334.2518
10381.52	1040.68196	1865.89856
229.945379	490.693143	266.378966
26628.8289	44151.4974	11538.5045
6867.09498	11061.0348	12859.8935
590.311785	747.177713	310.914969
44.4514001	39.0009936	48.4369963
3749.28414	2774.98258	3183.82165
338.953022	477.448425	1883.97934
913.051447	1274.55338	760.235855
630.349223	1160.13871	1692.0395
651.183553	837.339079	609.254567
119.072067	329.593265	209.939945
307.789791	194.388328	96.2991811
124.03298	344.005073	521.308327
377.212786	223.525616	165.469949
174.645677	588.079521	658.053747
391.963042	165.198145	605.209194
75.4598314	125.190009	142.470276
251.240117	391.744673	201.764533
6559.7205	4648.97903	3702.61709
227.941141	84.9764943	231.758632
461.873754	272.845919	225.271987
98.9623652	129.72898	93.7393711
231.866251	455.441447	759.74047
622.751254	931.64981	270.844388
578.251295	463.114766	363.747653
67.7935825	87.4457538	63.1487159
48.4285635	54.6449894	53.5036869
438.958128	435.198697	604.877639
34.6151748	73.2346415	38.4584216
234.721684	294.997774	335.376952
53.632392	86.0369281	53.9456983
251.803909	194.409087	401.333581
1576.56675	2221.92316	1019.44135
71.0539845	81.1768562	50.7562699
67.9694597	94.9090845	72.2121361
738.14065	361.454683	98.6509817

P109 | 001 | Geometric Segment	P109 | 002 | Geometric Segment	P109 | 003 | Geometric Segment
308.535699	277.524483	238.08126
1713.763	13877.5874	4443.29876
302.803653	521.829079	442.317992
889.854305	1182.87445	3302.2542
760.75367	1038.90076	4091.29376
975.746845	983.07703	8198.04242
217.230738	465.121502	348.838614
5375.04834	43796.9708	43503.9499
10113.0217	8679.8122	7102.49945
352.474771	634.425686	610.582611
40.0924067	38.6593857	46.0136811
2917.74372	2541.17836	2878.45091
3341.81337	483.554879	388.390926
685.682812	1112.38794	983.425295
1415.99386	1174.22054	1059.07676
478.762957	736.953587	558.687384
215.152844	254.129595	173.121588
75.7527291	363.401345	236.508706
410.674497	356.708538	165.546014
128.353376	191.85989	330.933873
742.276438	491.917076	151.79979
802.622131	189.253415	434.112256
118.72228	121.68928	46.5221087
196.357519	273.144963	322.39911
2822.73481	3421.36428	7014.97156
176.384377	74.7584714	173.584978
143.322508	213.951705	316.865169
74.5318637	119.773457	113.822754
525.435178	743.794487	254.029775
307.410102	2246.78746	1016.3172
265.285403	446.547144	705.38143
56.9018757	59.3654145	55.2587131
43.1280519	43.0389442	48.55958
633.452066	490.027528	566.004837
26.4903261	53.9583658	59.4133351
236.175956	276.541883	335.266383
40.5283716	75.9951712	55.3139863
438.301596	219.529708	440.058969
948.038661	1805.29627	1099.90596
52.08899	71.183192	61.9842035
64.7409911	82.3706348	99.6742616
122.802781	503.275919	880.258585

P109 | 004 | Geometric Segment	P109 | 005 | Geometric Segment	P109 | 006 | Geometric Segment
164.547594	212.745283	308.818684
1457.19112	4046.44414	21836.3795
280.082448	407.07499	583.145921
1275.53016	3287.60652	1475.10772
1509.98406	4011.12911	2036.51584
2918.24907	8175.91286	2570.5477
265.857828	324.9621	494.530517
21828.3061	33410.4702	62859.0529
6985.80207	8007.45651	8213.48235
346.990175	554.230912	644.63101
39.3117994	48.1163988	31.2276597
3469.97753	3841.78563	2096.51175
472.662271	323.188957	557.272784
1247.79044	916.477046	2203.1895
1118.2515	739.084592	1198.26391
334.7277	597.100212	768.24106
119.019141	158.530763	280.539145
134.444312	319.511915	399.59675
80.149603	148.97892	237.778674
265.969935	418.256821	282.703249
181.238638	176.226165	112.912392
1043.18626	351.389358	234.866901
46.403588	83.7691069	98.3320405
186.84004	277.804032	309.357857
2876.71947	5611.7932	5897.52122
87.7154897	200.894635	107.394148
198.151575	429.718889	255.136386
84.8099549	102.416162	100.894024
149.183977	341.89465	451.306692
747.607941	510.572545	2237.30357
338.988731	508.190424	647.329742
51.7433673	41.1782328	72.4890877
103.445176	33.3899076	45.9022482
884.457797	439.45934	488.078166
50.8541619	38.9214749	71.4639365
175.468154	267.417888	446.301545
58.6845399	57.0009837	72.3974293
506.677427	256.529013	318.767286
1669.98744	1342.60695	1716.35604
62.2662155	78.3830304	83.8956131
89.6239672	82.8985272	94.4268513
1574.17607	787.990872	864.830647

P109 | 007 | Geometric Segment	P109 | 008 | Geometric Segment	P109 | 009 | Geometric Segment
107.732502	128.751263	133.414324
661.337611	7902.13901	1409.67768
239.334404	246.52589	315.7885
466.338487	590.271089	643.858829
247.001917	507.288884	566.984448
251.725372	556.298165	749.767444
188.495116	230.543677	223.939458
3266.58626	21049.1328	11879.5642
5660.10458	4897.85995	5395.11499
238.473277	292.63607	292.075557
19.7404702	18.3430931	19.6738795
1417.44902	1421.18071	1491.49738
225.0161	209.366151	288.587724
689.815302	593.020118	598.432161
454.212583	530.448275	711.034235
299.842498	342.976669	355.360942
119.371479	141.406734	150.31344
44.8910679	118.184531	63.2842159
142.790813	135.751768	74.0313546
54.7336557	100.925683	89.9465945
451.572177	216.076421	127.808486
166.029284	234.590705	149.206606
39.0705846	47.0718847	47.2201353
126.218204	161.466469	169.585253
1102.66056	1872.84155	2187.47971
31.8300791	41.2741782	37.7438006
119.640215	115.101487	116.044944
50.0224592	48.2856317	56.4534008
99.4713179	155.065559	94.5134161
183.739096	850.838856	264.408328
115.545466	195.570141	233.324185
32.2146925	33.5772449	31.5923261
80.2566835	129.625559	128.74776
676.710723	633.597279	623.425458
37.9933028	61.4486906	61.2817394
105.904668	158.426259	162.337635
53.8275438	39.3166484	31.928003
359.163451	524.310379	580.820036
2093.82909	1592.33096	1501.24545
51.171212	64.3704904	53.2239892
60.074777	45.432368	50.1179592
85.5031659	134.133609	119.096975

P109 | 010 | Geometric Segment	P109-3 | 001 | Geometric Segment	P109-3 | 002 | Geometric Segment
306.175815	345.826872	423.27033
709.501316	1033.68661	377.81681
412.123509	418.878996	282.731468
1612.20858	1609.38877	529.289776
1250.63298	1428.2741	400.337614
1554.7366	1426.21537	394.13421
230.784161	222.592668	166.331534
6878.1449	7146.12462	1421.19584
6857.87227	9394.83492	6070.40251
488.224654	517.663957	275.894599
39.9034222	45.7167749	58.9343285
1475.81681	2131.6995	3646.86259
492.578404	716.190481	2367.60053
427.940931	482.022844	313.326601
806.31346	801.610076	648.783575
532.912303	661.43368	315.507526
194.669985	214.608458	200.868674
69.7422894	110.939334	25.7785593
143.712589	266.443775	187.972778
119.440243	155.633435	77.4942022
341.214248	815.222614	950.470855
160.91606	205.907686	230.071648
102.170928	122.689457	99.55486
159.587393	203.648821	199.231041
1503.57387	1445.99905	454.70276
123.382286	132.712312	176.96413
142.116752	170.549547	54.4133477
54.7412997	67.0356226	35.7406231
158.301033	204.17477	262.246317
150.17226	273.246433	104.5576
417.315444	376.694525	154.841138
20.7499168	26.6349714	35.6644057
101.337449	110.939103	146.518857
651.660698	689.235944	469.712343
93.9310043	88.4623357	67.5829614
197.49615	319.703678	121.559086
35.3332991	45.7381042	32.8894558
545.948304	512.851704	374.9969
1668.00232	1921.0796	318.006076
87.1940148	110.592959	65.9886894
59.6547488	84.4178372	33.409004
215.460464	316.548016	53.8098911

P109-3 | 003 | Geometric Segment	P109-3 | 004 | Geometric Segment	P109-3 | 005 | Geometric Segment
232.744142	242.182539	417.733138
409.779207	567.102802	440.266755
271.608845	305.59125	326.627372
628.134167	828.572171	825.878885
449.736486	964.598788	552.140997
571.921262	1307.05278	734.460371
155.175857	186.534357	202.918746
1376.89463	2295.31361	1954.51001
4391.46731	6840.26227	8391.43678
270.324194	316.360307	326.607272
26.1693258	34.0268315	27.4356663
1617.11277	1634.3077	1936.76339
983.023468	1482.26503	619.443443
351.559425	457.945478	395.190527
374.569924	510.906359	559.997668
306.393734	358.343227	388.452342
127.387215	145.458955	173.558136
32.7889072	55.7102864	60.5308966
75.7172514	94.3735498	214.391823
59.2838662	109.529048	106.333412
355.727115	459.298337	721.387689
105.100679	219.037263	121.024547
48.0486819	53.4777577	83.4315567
136.447566	180.485274	160.437629
647.127725	1274.05683	1533.42864
79.024602	103.132451	173.629561
63.6275117	108.410992	98.5773293
31.7041185	43.6659632	43.0142564
82.8906226	82.8097823	172.951465
141.986102	169.390856	112.580808
184.898517	291.712695	236.543118
8.82690126	21.2079328	27.7621119
128.898153	128.344949	68.8755475
648.744695	651.304668	646.633303
75.8553803	79.4601101	70.949704
91.9647447	134.212586	169.391998
34.0488931	38.5978966	31.9781395
507.780478	639.602883	520.003734
1336.60602	1435.98114	1113.07723
85.6841535	63.9363292	68.5147362
44.8962706	41.1268739	46.4877163
64.7753803	182.238685	95.4982655

P22-2 | 001 | Geometric Segment	P22-2 | 002 | Geometric Segment	P22-2 | 003 | Geometric Segment
361.435836	320.863791	535.850697
592.471112	499.24477	999.429364
311.490996	291.355071	461.755294
945.233619	591.153243	1562.58637
719.551076	433.496687	1926.1772
822.049268	486.921727	2128.88829
181.894353	193.763447	294.639859
1845.90694	1682.16812	8547.36962
8235.36471	8232.76717	12727.8884
358.555512	346.275003	596.789844
27.5416596	27.9242833	43.6935987
2175.29376	2158.31981	4183.68494
414.782639	1715.73671	1670.74231
463.420157	423.981348	615.337546
647.24918	600.987293	1165.68722
554.494073	361.056749	807.141076
196.655701	172.251614	268.054637
70.2535377	46.5869958	182.917802
322.889492	170.476598	513.518753
102.396429	80.8607431	203.255328
793.074255	534.793498	980.416074
108.063774	108.586795	389.610976
130.798705	89.3735983	185.028543
142.595136	142.531154	178.129422
2106.81698	1053.45104	2827.76727
150.650599	123.626785	264.863211
135.134908	86.6361803	208.365546
50.1296207	43.5315694	78.9734301
309.861698	143.215408	336.556765
236.382784	162.926716	388.038068
261.112303	207.239069	591.887399
32.6397985	33.1131361	28.8348853
86.1017994	122.290609	114.097005
719.035137	681.620643	907.383346
76.9987102	78.9832823	132.266466
188.675504	169.596308	435.049011
39.5470124	28.9759292	50.4644194
479.46398	499.458714	595.199827
1316.22709	1120.01494	1547.11824
57.8325555	94.9170197	108.851898
49.6628513	49.1639091	66.1894231
122.928822	85.8366587	600.029044

P22-2 | 004 | Geometric Segment	P22-2 | 005 | Geometric Segment	P22-2 | 006 | Geometric Segment
233.373688	187.95373	385.522577
805.690859	957.733192	430.521799
357.182233	363.927656	273.658999
1092.55698	1269.42107	654.458652
912.93869	1373.11774	676.973795
859.727627	1095.86902	880.646147
189.13862	204.072534	144.579088
4751.24558	14336.1892	1511.83101
9023.95311	7401.26773	6071.12981
343.240079	426.195351	333.480622
21.3456139	24.5494387	31.3388926
1976.42928	2644.4493	3003.83461
239.654912	626.779339	3480.74417
455.360369	555.770396	347.91594
675.550885	1056.40435	860.4955
519.022458	553.688616	294.348011
201.673575	169.513733	194.954412
102.345904	166.166907	37.9200463
194.735874	207.49807	168.195734
145.137299	279.075984	76.5712752
384.121757	718.849848	848.647215
198.199018	404.481633	170.819171
103.383349	113.390433	93.7970145
194.325967	214.101842	170.137898
2268.82496	1728.60717	788.395411
70.5894352	107.650524	151.788299
125.847193	113.740619	84.3373514
51.6756496	77.5614965	44.5955296
107.129758	133.411206	158.517206
213.895672	272.270604	108.195004
244.163662	276.799306	263.930412
32.2935144	25.8766065	32.0167021
99.851887	77.8174388	176.289545
723.17232	886.648671	460.446997
76.8062521	57.3814023	76.1282334
267.068318	334.483153	138.015705
44.0756497	57.0617282	33.7341354
606.031272	642.39558	480.494553
1743.14558	1531.50494	617.558244
68.897298	113.247648	86.0382663
52.1252936	90.0839713	49.591134
232.855126	546.100707	116.931901

P22-2 | 007 | Geometric Segment	P22-2 | 008 | Geometric Segment	P22-2 | 009 | Geometric Segment
348.287888	516.567946	737.68673
2034.4484	1555.98338	2327.55466
556.881495	838.705857	693.290886
982.093328	1654.50093	1332.697
717.036265	1294.55507	1258.03143
672.710529	1147.1213	753.27024
469.961293	522.130045	513.62868
4075.14524	4859.59598	3810.06133
19047.1564	27313.6379	23143.789
502.322822	643.534702	634.981998
38.1087083	36.4045342	39.1107202
2124.49065	6523.01408	7009.91866
452.700143	668.703355	2610.05177
570.61063	970.720918	808.224903
1337.49102	1989.30263	2836.50695
897.432386	1021.86115	888.126264
333.025707	477.406318	526.087826
102.868774	90.3970382	121.722941
275.324731	543.624031	671.266566
108.530097	108.324775	169.461625
558.747962	745.169466	1492.35001
233.754265	362.708489	412.283457
141.447605	295.131293	219.502348
197.853314	253.838577	272.245049
7067.78132	13786.5902	6537.58412
59.716845	162.539453	282.534583
241.263862	207.769725	183.746141
80.6800059	114.765609	103.828955
270.543398	279.615783	836.745724
194.571115	178.22046	255.61938
180.115183	546.730426	381.953234
60.9526373	43.4785735	68.8367016
109.268924	82.0104902	116.592542
1181.7573	1218.46401	1081.03899
19.6901136	33.4942915	60.6048677
291.170767	375.487813	286.418352
77.8094994	95.0428458	71.305201
407.095765	974.375231	647.563251
2092.96827	2022.69197	1376.28211
57.5292798	150.278495	131.101991
77.7868905	121.470442	99.3280351
137.587533	366.164138	241.551273

P22-2 | 010 | Geometric Segment	P22-2 | 011 | Geometric Segment	P22-2 | 012 | Geometric Segment
644.3137	368.426336	520.73545
830.241303	2111.27522	1777.87047
268.994578	430.860619	396.079095
407.481271	607.624895	760.569881
654.852487	654.111318	787.350435
669.331497	424.500112	901.123031
194.7942	310.630321	282.958402
2645.96204	3642.3919	5690.38445
7056.04098	16764.9869	15370.3735
244.794024	356.116922	397.499225
85.0445278	46.2868692	45.8693611
3070.16047	3116.59812	5037.30456
2926.99921	1678.3738	1597.34425
458.692132	436.037025	383.266019
1300.93148	1639.36629	1361.81399
320.471844	506.076644	655.984028
222.160507	362.90148	299.992568
43.8807816	46.6548306	57.486696
334.019748	446.833529	387.0463
53.6787989	87.6466814	83.3943588
1121.66093	1256.79499	934.236135
472.220623	247.676744	319.318072
56.2506808	121.664121	130.944734
311.028674	248.605387	276.41851
860.834095	2108.64082	3655.78823
219.857163	79.9347288	116.446551
117.854386	174.134971	196.247822
54.8527664	56.5991779	72.9063639
566.941583	409.718128	388.772189
144.538794	251.889421	206.821183
177.271833	109.310312	193.320953
65.427019	60.3067849	70.8499618
133.078341	86.668441	82.0538436
656.9581	702.296402	740.564506
132.393835	26.5475004	15.235656
229.44619	268.692625	260.907468
64.1402404	70.0228891	67.2005211
554.803429	390.007284	359.41091
484.768074	572.591306	1041.21905
42.9875706	77.752621	66.0848548
74.8395829	79.2023978	55.7973847
102.449905	81.5126533	100.360068

P22-2-2 | P22-2 001 | Geometric Segment	P22-2-2 | P22-2 002 | Geometric Segment	P22-2-2 | P22-2 003 | Geometric Segment
267.081059	389.230162	301.136571
888.591741	485.328371	749.135466
260.176136	240.55022	195.988317
461.82873	625.672468	346.824562
729.877666	1751.37185	557.766679
648.560104	2129.76658	452.066546
280.393727	149.380038	160.121689
3384.23802	2288.01721	4031.19371
5391.06915	8169.35246	6394.01256
225.931616	233.813492	193.771231
37.5856328	61.5607226	30.8578964
4100.92919	3369.43153	4213.56189
1944.97969	467.227435	1246.41693
428.479083	478.804743	417.413415
1325.98541	533.628968	831.246284
386.338997	470.737035	321.193017
166.084253	168.557668	212.461016
48.5323701	52.5778018	51.5643693
274.800548	388.313006	421.558607
73.7930211	83.5688556	76.5958026
747.178689	1056.61821	1047.92254
366.648006	571.019186	470.859853
68.8349721	96.0137022	79.3014546
199.620236	279.975357	251.940706
2578.6399	5079.5204	1461.59985
82.9345188	141.119254	89.9753349
131.234301	167.635071	103.895693
80.0343384	89.721475	80.4927746
342.874564	357.721408	505.468688
211.460556	137.153343	201.821199
132.18549	370.906879	120.38024
53.9951061	87.6099711	90.993392
79.6021853	121.107378	68.6212682
621.407093	828.125392	568.482656
29.4240144	53.7191065	28.9886253
253.56551	215.535059	226.993983
59.0888375	69.321755	54.1530196
396.975247	428.917463	355.744933
705.692085	1642.70952	593.834369
39.7368522	52.2029199	38.2313118
64.0541103	78.0197258	59.9954235
150.805549	350.102778	324.312801

P22-2-2 | P22-2 004 | Geometric Segment	P22-3 | P22 3 001 | Geometric Segment	P22-3 | P22 3 002 | Geometric Segment
367.649682	332.48409	211.127147
1708.35187	3944.07739	2181.54637
455.488047	462.220542	356.305563
728.26402	1037.06564	636.043916
719.170311	816.185072	729.875416
568.830276	795.154	751.103852
320.724794	349.257846	263.085995
3452.18468	5797.79657	2470.5999
20614.0806	19544.4396	11146.1784
475.889848	445.564233	337.210328
32.9683925	47.3972913	22.8470985
2416.56755	2551.86624	1630.14927
436.147742	432.369274	274.554164
567.689997	689.218914	530.247197
1274.44399	1350.90004	942.374572
942.682641	796.94664	678.92536
273.915073	300.859585	251.375803
59.2228946	114.233856	62.3575829
375.700911	298.296441	194.555293
115.955324	236.220473	59.2104565
700.319933	547.018445	234.353574
249.909143	355.33584	155.943322
141.403439	133.275697	112.839957
170.475567	182.573931	152.545143
8612.74976	7556.49085	6425.1879
78.5920437	77.4326944	80.5411351
281.889094	273.396477	193.608083
96.5283679	85.3998974	55.2991774
284.344091	318.459404	167.346477
221.884634	379.070198	216.332951
138.259546	190.236063	197.569697
79.5183549	69.7444451	58.2732503
103.687726	118.914281	159.679115
1190.58816	1201.41365	1013.04502
17.0342009	30.1407564	18.887524
335.933751	265.361554	159.11405
91.4860068	74.7804641	60.6432961
484.771483	527.594917	419.488968
2028.63901	1981.00489	2137.60212
57.5113167	67.5763372	69.7468569
50.922898	59.2031425	73.595347
206.859798	286.405858	117.392133

P22-3 | P22 3 003 | Geometric Segment	P22-3 | P22 3 004 | Geometric Segment	P22-3 | P22 3 005 | Geometric Segment
121.881853	699.597694	246.25984
506.035116	1538.31234	1212.11501
171.195111	269.825742	266.450554
1793.30605	698.796527	524.606098
3266.93791	847.257541	582.827373
6132.53613	1211.89602	785.363393
162.229534	213.667408	180.98046
2715.41112	4130.10944	3436.45951
7210.69524	14813.557	4125.42369
244.33883	242.587559	260.803336
78.7557663	43.1637071	35.7990888
2285.15401	5228.32437	3101.2515
292.91218	3171.13116	3077.40266
506.61101	497.58691	670.90009
440.255042	1224.50331	1167.7845
485.361275	419.555204	315.030927
84.6221931	218.582073	132.219453
162.210597	60.3929889	38.0611639
124.51444	730.648405	329.88218
352.761446	112.013977	86.2058231
208.652527	1803.21031	997.085749
920.500775	481.19638	355.482576
48.1908437	106.834962	46.0073327
210.91775	212.948855	164.920259
4397.06993	2045.49364	1339.41089
232.525615	536.078892	89.4116169
483.977744	224.09713	124.276649
139.972154	87.7508801	68.2921364
169.483895	890.146674	204.166392
188.802457	171.179959	165.382076
287.767287	274.613869	229.124428
38.4495122	59.3068632	29.3950898
122.975459	75.926573	98.8202492
577.431956	517.851484	544.931464
50.9378143	32.925967	87.1463296
106.653412	285.852117	180.885849
44.5462013	59.7082718	44.718296
338.047116	294.31075	506.056202
1743.7246	406.190416	1110.97481
59.6456769	42.7925138	49.0216972
81.0895543	68.7312874	90.7321392
2901.34737	222.838826	208.374952

P22-3 | P22 3 006 | Geometric Segment	P22-3 | P22 3 007 | Geometric Segment	P22-3 | P22 3 008 | Geometric Segment
315.97716	193.899125	179.88947
515.226581	606.398428	736.721954
193.527626	265.036178	319.507272
428.972441	1781.26373	1560.19191
456.947621	4150.68011	3448.5338
771.930357	10310.0115	8638.72207
134.869451	188.794209	224.785413
1458.54225	5273.6206	8069.75057
5763.37625	16959.0895	15552.9774
195.40751	450.190548	486.840152
38.6550371	58.3502782	51.5413006
3624.42444	3078.35692	3490.64642
4795.83996	406.185859	505.568832
519.975147	756.402209	716.956365
817.446486	689.334343	1023.18115
236.246097	720.948911	679.271485
176.034999	123.286324	130.124936
41.7708031	134.532202	152.145301
240.411021	173.702465	170.690475
81.6931634	343.478659	404.594037
1186.53611	142.963878	163.944254
395.67462	985.075429	1093.36218
54.4987046	81.19326	59.5328696
171.695682	210.097653	247.474684
1478.84094	7798.47425	7637.14045
158.108311	289.556563	327.899229
122.626001	622.999154	525.356564
58.2454921	152.264732	140.601559
301.631261	343.158606	398.313435
110.287778	190.271587	329.234126
176.879984	308.251462	332.72159
22.3498464	54.4811649	53.5412661
68.1325843	89.8276874	63.3849001
445.073362	692.034852	709.053572
49.2793993	40.2344471	45.4248614
201.390117	132.268975	146.923649
41.0679907	67.3752834	67.7102613
327.381349	358.074802	384.369767
510.423667	1418.93389	1339.62771
43.0936201	64.4616387	72.8071807
58.4412564	110.294992	93.8906637
276.578446	2726.63602	3049.64369

P22-3 | P22 3 009 | Geometric Segment	P22-3 | P22 3 010 | Geometric Segment	P23 | 001 | Geometric Segment
345.54121	687.625343	186.376756
2448.84678	885.80561	645.530895
609.927429	182.443796	281.419784
1563.60238	426.028764	1518.6873
2230.58592	700.787177	3507.18643
3382.5837	910.768819	8680.24878
408.54957	129.744023	223.903394
6967.26665	2929.32836	9326.85875
10056.1446	7757.00444	15443.3828
684.073354	170.07566	386.248273
48.7724717	66.1346561	49.8189489
5782.95157	4746.45921	3569.3045
974.346909	3957.89191	330.691908
1069.57351	408.491177	803.70502
1113.07097	1095.26261	722.763233
839.617638	373.905746	763.710163
284.102954	224.951603	116.792521
226.590261	41.0919134	165.698628
179.82977	574.132998	160.834322
232.787449	97.5560552	367.273389
359.536384	1537.91136	127.988776
198.482962	747.567033	945.838695
142.433612	136.630643	92.667397
316.057875	207.265599	202.471912
4389.75723	1995.09451	8054.46447
202.69828	458.651113	280.455165
259.431495	155.917185	553.161623
111.353607	102.006419	134.779922
172.836171	696.050306	394.638259
463.023601	124.912353	314.305894
851.835853	199.642622	314.45165
39.2050579	49.6529029	74.6530697
55.2297596	62.3905194	84.8631628
389.447727	458.163996	736.505391
52.2176089	11.9232633	29.2927281
161.081121	146.989514	174.713093
75.7121582	41.2040493	50.9036832
288.938667	222.293537	326.386229
1354.48213	220.180967	1403.71882
75.4351359	43.3240014	68.7421343
100.435516	49.8251999	93.2640827
287.5421	87.7885187	2839.35946

P23 | 002 | Geometric Segment	P23 | 003 | Geometric Segment	P23 | 004 | Geometric Segment
192.858834	168.391779	127.473082
606.792539	2278.37162	1414.81338
316.102287	362.209927	202.776803
1645.22114	1388.20123	2637.11592
3559.9582	2495.96495	3832.9607
9432.95756	1574.13939	4976.12743
238.726305	205.103046	146.062508
5856.45249	29758.23	14139.2736
15502.8726	6773.88298	8755.71224
459.644089	302.603062	460.745937
63.5310481	92.4940137	144.521986
2500.7891	1820.67839	6527.63925
648.786301	242.685742	218.653686
792.980385	477.429329	472.365476
549.163908	1967.7337	465.680652
662.961777	563.231789	640.585123
113.686657	163.615593	95.5721314
140.653471	326.072936	229.250714
115.302415	554.108347	350.283827
353.844324	547.838661	268.603024
157.400268	488.941356	208.523985
873.902165	1399.86934	803.453577
69.3496898	103.727559	111.247158
202.812542	247.74358	294.080937
6913.37623	3238.4074	5908.44791
310.748755	131.252626	283.082137
615.658045	129.697826	264.894684
143.010114	97.5328059	54.5367759
323.242753	1513.52813	271.474039
192.282287	715.103402	894.453499
278.0854	285.458851	513.337153
47.556124	105.860101	60.0635815
81.4901735	53.6803655	127.126995
664.277482	962.130013	706.702481
45.1546596	52.2959993	53.8318804
172.194991	565.094964	238.575824
61.3476211	64.8911332	48.5431075
352.186431	703.192691	528.831399
1441.64917	1412.34944	1881.75583
60.4397586	67.0902996	64.4162431
92.5855142	63.2489046	71.4130703
2345.2156	365.100579	1628.842

P23 | 005 | Geometric Segment	P23 | 006 | Geometric Segment	P23 | 007 | Geometric Segment
184.381008	134.164933	192.110907
8802.26172	1292.3881	1228.42119
402.643732	258.100466	345.41511
1733.30855	763.77649	1701.32877
1978.05806	1292.22451	2658.66985
1715.5633	1155.82355	2693.32592
240.827652	132.008468	192.825079
39518.8272	15435.0913	18448.6856
6199.24626	8145.11714	11122.2891
374.998285	207.419865	300.595911
99.02863	62.0602664	130.41104
1187.19309	1607.86292	3769.82385
222.712513	166.741701	646.021518
507.319075	372.795197	586.182389
801.687712	396.266875	604.302185
427.285289	446.113948	635.467983
128.141321	160.436173	229.864336
263.360231	154.263887	223.561488
393.120544	354.278623	431.886522
297.635636	164.222078	267.620749
113.949798	550.211099	707.04491
1173.60095	434.268947	881.178662
70.5477513	124.49981	95.1342106
263.801387	196.805641	242.262812
3483.16638	3588.24822	3989.14057
127.799341	101.401648	221.276937
139.762551	142.943311	170.801776
71.5691046	57.9621355	81.1995909
654.052851	285.960183	450.005578
1542.53683	1247.00261	1293.84668
296.075433	252.990059	575.021348
51.6692588	51.8089844	58.0636762
54.2731905	80.1286964	57.9666576
826.842091	708.668348	796.531836
61.9896628	68.5613823	49.8711576
418.089177	448.06932	291.749596
49.8936968	51.0708838	64.3910262
565.681645	445.692915	475.187711
1264.87006	1804.08583	2549.90759
66.969479	30.8472977	104.948798
65.3609154	63.4173301	82.2259342
427.081738	215.831279	839.887944

P23 | 008 | Geometric Segment	P23 | 011 | Geometric Segment	P47 | 001 | Geometric Segment
150.595676	260.623376	349.922941
1853.696	731.988342	1009.22575
265.189111	393.028323	568.250049
2824.50286	650.922985	1264.95791
4257.94429	725.132893	1042.19857
5228.6864	1085.84283	1351.38249
173.563657	323.627298	400.814322
21028.2916	3756.91229	5358.0504
12323.0851	15695.1664	16869.9483
417.313188	412.794453	568.247891
72.4191446	53.325869	42.6432302
4970.4532	2269.71387	3039.64765
295.75985	337.40649	503.150931
603.289771	477.815035	625.337885
567.242482	695.747145	1294.42576
643.510366	629.57726	855.853144
141.830005	256.199343	330.80416
209.160607	64.9144611	127.779221
357.185617	122.896835	115.49673
276.312637	125.95597	265.861487
288.59734	539.361936	575.041816
1305.24124	319.317721	463.592881
82.5459514	208.885846	201.390027
246.671648	135.542455	199.177418
6027.65332	5577.97466	5824.36283
319.068041	94.1960228	102.252445
248.853429	187.114709	213.728047
106.193308	83.6020053	97.5326866
304.154494	76.1297623	160.858819
1083.70474	191.411992	253.858912
604.123429	218.193987	264.175607
83.2375615	65.0975181	58.649733
99.968096	63.1047746	77.2182225
739.100798	893.318755	937.096076
88.6347489	31.117405	41.7182894
316.181083	265.185576	294.849113
66.6284573	52.0273809	78.8497999
493.605137	408.231878	541.343844
1883.07993	2259.86468	2153.21326
80.1158072	61.0036501	62.5422314
85.5720681	106.15738	139.444574
1249.65312	80.9740151	100.133029

P47	002	Geometric Segment	P47	003	Geometric Segment	P47	004	Geometric Segment
300.135634	324.631128	336.100899
1023.45626	1287.80928	722.96071
459.119719	467.425004	449.914277
773.854999	917.923374	832.590201
701.326126	816.083888	703.38528
915.573278	950.753003	955.287088
334.996943	336.621293	328.000283
4998.24401	4711.33359	3292.26213
17063.4926	16874.4453	16161.5061
399.707703	475.252278	414.466603
30.7982032	36.2762229	42.2385957
2607.74451	3050.94895	2613.21913
366.555701	503.534731	442.698603
677.544984	545.144538	543.213152
820.335369	943.311417	759.251112
640.68135	749.302064	666.970887
267.917382	322.211365	333.831947
73.897742	78.3477578	43.7050791
110.948319	127.693624	91.2606388
168.339849	161.2345	119.819141
450.814416	448.910617	369.553633
295.110404	395.037628	311.914547
192.365273	206.425528	187.643781
146.963358	158.634083	131.219099
4960.45391	5284.03972	5725.25233
106.6418	88.154417	66.7477755
176.702281	206.421184	182.242734
87.9263468	86.9848906	72.7847541
106.525476	113.072249	55.1535427
233.333643	267.217501	134.678817
236.801536	223.445692	225.293748
38.3959775	56.2750993	68.3596889
57.8601277	62.5390079	63.4881426
902.107348	858.09256	895.279464
90.3643188	26.2634909	33.1794817
320.836166	264.32832	203.392897
68.785837	58.3262992	60.471318
453.418194	376.456019	403.041146
2060.89578	2153.07787	2078.60331
68.7713108	80.8788576	57.0445002
156.903026	98.2780222	107.296752
67.0452084	119.07202	92.5706907

P47 | 005 | Geometric Segment	P47 | 006 | Geometric Segment	P47 | 007 | Geometric Segment
338.886047	503.048134	345.137978
1014.21422	1691.92422	996.612412
488.864118	861.531764	444.979658
1035.20794	4020.46164	855.940411
1208.05153	7236.40169	991.396324
1485.31931	12409.4531	917.482574
372.999663	647.816804	403.87955
6641.07455	32701.7834	8334.48025
16530.5327	19531.2004	6335.46713
477.61103	871.531077	447.588984
43.4253969	120.685093	62.3001307
2837.73576	12100.4093	3544.7277
538.504406	922.974009	10053.8091
628.646674	971.404214	592.350006
1055.26901	2840.5621	1964.61809
734.224626	1416.05863	512.979219
292.646175	341.8754	295.44582
76.511445	331.818386	63.1991484
106.743837	257.936506	164.165402
190.534375	790.22384	132.245542
337.306871	278.078268	1224.5905
397.260256	2107.29331	683.889647
192.693773	215.913477	118.218765
175.894232	305.579262	195.497562
6363.47025	13760.8728	2264.13632
96.972978	428.812119	89.9087924
198.314175	490.054926	118.806752
103.479844	171.544278	87.3180421
103.458511	315.171958	197.849113
258.336192	435.421172	281.073061
266.080867	662.870872	192.197111
56.0129735	103.913496	64.0175437
70.8885592	148.932505	132.123186
868.367631	1153.39891	738.62524
19.2359459	53.7051038	72.5012196
264.136092	531.951588	358.580916
65.4425412	110.332088	67.2921002
433.681908	712.503737	837.917554
2206.7223	2493.0739	1162.715
67.1811304	120.67127	66.9072909
94.7897129	222.215261	93.3041201
128.803806	4426.7102	146.290531

pSLN

P47 | 008 | Geometric Segment	P47 | 009 | Geometric Segment	P47 | 012 | Geometric Segment
269.60361	153.645794	285.088189
726.916964	481.482233	378.168673
434.071111	259.050826	314.748946
768.732944	690.039689	444.207064
552.325744	623.835551	357.458095
560.432765	893.177268	275.325735
321.789668	176.15872	210.936022
5393.58261	5247.84902	2137.49111
17084.9148	3359.38325	5575.38565
419.283615	283.811003	308.693588
29.7304125	22.6506639	35.8402669
2691.54326	1296.0626	4518.24258
395.295077	495.992868	11176.794
658.429068	603.962044	365.465309
802.79513	1218.38795	1605.9555
671.352117	300.220934	286.420156
242.570905	128.375662	199.696657
54.770566	107.862697	45.5765016
95.2485291	828.378848	245.880062
146.99886	96.4720661	32.8780883
359.286987	40.3427268	895.592758
352.09565	772.672305	283.789213
208.863967	29.3662836	90.9832156
173.081156	278.400561	151.157005
5827.68488	3884.95902	616.829025
85.1809536	85.7310975	121.262259
176.436382	82.3363908	68.8278631
78.6203533	86.2647641	53.5570403
98.7676656	138.821186	241.147525
150.039372	175.937786	88.123533
172.89915	170.940555	158.126861
36.7424654	73.9006808	38.3260115
63.1921927	70.9224813	53.712626
939.781689	456.777257	384.933465
41.9189495	32.8878502	38.2101767
184.362167	88.4596591	164.694272
54.564295	46.0515359	44.9374104
402.873174	551.068501	344.381587
2068.50448	868.203171	77.583473
47.6964128	266.791317	55.9662369
107.898471	10066.7213	145.716892
119.222368	33046.2294	505.413379

P7 N1 | 001 | Geometric Segment	P7 N1 | 002 | Geometric Segment	P7 N1 | 003 | Geometric Segment
555.520737	540.767229	569.289744
431.196241	335.980314	318.73219
359.067567	273.11588	288.07294
561.253385	423.027051	452.114031
760.953326	670.513046	648.522129
1137.42313	979.465125	823.043111
252.013089	189.487841	166.39782
2778.40844	2615.34895	2765.1551
10838.482	12113.0472	8298.44244
356.836543	294.652732	301.097207
72.4425023	61.7457409	86.7314564
6491.19171	8155.99861	8717.50924
2372.68522	8025.40661	3141.46517
520.65984	352.445719	416.313644
1262.39784	1421.81072	1179.85652
364.973152	350.403476	383.738208
259.757575	287.915245	272.560675
48.6248039	30.1205418	44.5903319
456.902838	270.335769	328.849808
110.501037	102.187674	79.3124244
1106.22424	1104.32555	1260.22865
648.500891	361.080988	345.326476
151.504277	195.037072	230.45453
191.842928	192.706236	185.047496
2068.07729	1022.3492	812.694738
476.112078	717.605957	453.220076
157.722139	136.363424	135.942033
66.8477892	75.4692684	53.772314
174.726978	154.309935	223.127268
134.903601	76.7370635	128.611983
380.154688	283.584663	280.107598
53.5319019	47.2977448	50.6600279
70.2307928	41.6996587	62.4889787
595.844715	457.318976	496.278903
66.1566982	31.7858243	42.4258089
182.945145	142.005345	158.844228
50.4121774	40.5579623	53.060238
449.217496	329.215606	300.908504
542.758217	207.141763	217.833029
120.751503	60.3306422	59.7508176
1161.33662	205.257936	183.856215
7939.94988	980.346425	1810.90771

P7 N1 | 004 | Geometric Segment	P7 N1 | 005 | Geometric Segment	P7 N1 | 006 | Geometric Segment
201.534805	435.543931	389.166943
359.916014	404.311622	454.826193
272.445153	352.605981	221.479084
428.133459	454.586612	353.124989
415.560593	590.04151	581.243302
454.938845	751.788599	511.524115
199.244945	234.362849	180.887662
1144.36571	1900.2033	2124.14554
4665.37244	7887.54463	5748.54196
296.317128	352.703724	256.345643
25.011411	51.7075193	54.5698303
3325.61428	5833.69544	4418.57555
12220.6664	4827.61706	8947.22042
342.645447	521.775848	396.227509
1050.04475	1395.10506	1795.9438
212.327131	312.389308	285.611283
179.732711	267.680704	225.659369
30.8105024	45.6636667	47.4264723
70.9755853	151.791604	258.599126
54.2318198	105.557999	69.7259598
259.072465	690.980264	1537.63919
177.988854	351.494759	322.978272
82.5413546	147.841096	149.038766
226.315633	168.446025	185.706671
575.359943	1316.45665	609.277311
96.8600461	299.187564	270.781934
88.7865415	85.6029565	78.5971716
45.110493	89.5411795	66.4984539
75.7654358	75.7515471	244.537141
78.6732497	95.1837919	79.9901661
184.180776	291.997823	176.80239
45.1557657	55.7704265	68.200126
59.2824973	65.1336595	161.739259
361.044814	503.39312	562.168454
39.9171029	96.3765721	45.2051441
86.3327105	100.195286	170.044046
38.7212086	52.0525856	39.1404736
357.044378	457.847369	501.035003
198.578101	397.948504	267.507255
71.7218938	75.1751564	54.6540589
80.3792648	150.540068	112.373822
163.549912	554.997373	313.521739

P7 N1 | 007 | Geometric Segment	P7 N1 | 008 | Geometric Segment	P7 N1 | 009 | Geometric Segment
123.92937	254.201172	415.704806
528.314073	677.082117	326.216144
144.917688	338.372118	216.643691
443.240115	638.364648	384.469311
351.974918	810.203179	473.723448
404.891824	1001.52609	742.472305
146.626542	230.123052	160.758042
3644.6415	3305.17537	987.903714
1624.35416	9682.77899	7512.72997
191.282835	400.144001	221.804203
10.6771733	31.5325377	81.7529219
826.327511	2195.85308	4612.8185
887.786741	961.719834	7631.95008
382.554512	637.648516	315.285181
752.73794	898.645342	866.78287
211.592282	376.069193	255.698898
70.2791136	217.219798	201.896399
78.1273913	89.2159881	43.0717209
1045.31258	633.523852	274.746731
82.7653056	137.524325	93.9826241
27.7330354	326.301724	879.315782
518.350817	638.599878	776.109778
22.0226566	80.1277683	139.587282
289.10018	232.99686	284.562791
3945.67933	3958.73857	1770.56758
37.7181615	99.5973569	458.612044
42.0342404	99.3106495	89.0459411
59.5773097	64.1231508	87.2081054
115.628601	127.1364	104.69634
184.71743	100.999092	84.10936
115.227597	306.505986	186.379693
62.5526928	57.6121991	61.9314553
64.2414265	63.9634338	57.6226266
471.350123	699.248513	408.112541
24.663585	59.4359207	19.3713604
64.8643608	130.261984	152.04317
28.8326544	49.7094875	34.5537657
553.201489	563.560511	327.740144
506.650512	1086.86403	114.948991
226.084533	137.082819	71.5335425
7829.62231	2216.84326	127.863342
25861.883	11962.3629	1505.50621

P7 N1 | 010 | Geometric Segment	P7 N1 | 011 | Geometric Segment	P7 N1 | 012 | Geometric Segment
816.800318	449.738389	449.621309
474.795387	404.483557	333.068912
265.570538	244.73048	247.028098
470.030307	424.323853	455.494938
898.112891	540.437774	680.281031
1563.86607	672.843988	1207.19793
192.846742	192.428092	185.551277
2045.7269	1522.07605	1682.03152
8434.10667	7667.55186	10210.8264
311.369369	258.043666	240.262737
97.5038582	67.2909466	100.952455
6511.2219	5455.68361	7590.38863
8341.63352	9764.68573	9608.24699
389.181253	318.329865	352.801528
1591.03871	1579.2075	1070.839
322.016942	278.20108	369.901882
209.581243	234.476994	230.907213
46.2873728	38.685196	42.1735624
627.779996	322.63858	386.506534
118.277816	100.566221	88.3286533
1560.03844	1165.35687	1136.82329
1258.02022	698.302854	669.279972
164.889453	142.020706	174.518831
280.636438	264.787284	225.804933
1393.37852	973.349717	1764.99392
884.27972	452.204589	469.006808
181.030418	77.3427478	140.640505
76.9936767	59.287232	107.147511
170.429176	134.97707	292.020671
111.084722	92.8403145	85.661214
299.688514	128.846555	193.836854
78.0231011	72.9105041	89.2108011
132.505652	49.6186615	36.7479164
603.875757	444.28218	396.835739
49.6152536	21.6901745	29.3402226
295.374947	202.459341	236.365379
57.9282342	43.9451767	45.6748591
572.380324	354.457353	298.822547
62.2309096	49.3767602	89.995725
65.2331642	56.5385911	67.9312431
121.70038	119.805554	102.454607
862.720155	1547.58747	160.538327

P7 N1 | 013 | Geometric Segment	P7 N1 | 014 | Geometric Segment	P7 N2 | P7 N2 001 | Geometric Segment
647.53556	944.340113	621.677676
341.867318	379.357795	337.822408
255.655129	224.224865	252.178148
420.290678	401.233344	370.11834
696.343058	805.442642	618.429786
1212.2383	1589.17871	945.268889
177.574076	170.879655	176.30615
1852.34715	2066.01286	1819.39788
11400.2098	10992.2757	8010.65178
249.10811	243.459417	266.941267
83.9797732	100.887883	92.6950086
7695.25231	5780.90843	6987.12899
8344.39805	5266.90667	7840.09311
303.500103	329.857589	343.675093
1241.26923	1114.09835	1401.36335
351.155706	318.89663	266.880365
273.986245	241.629271	245.558092
51.3411384	35.2977665	32.5163015
439.476633	409.553316	317.227745
149.138892	151.740565	116.577404
1360.96293	1706.7386	1447.29558
955.790768	1018.0205	742.567448
199.664275	169.029008	151.056248
225.950826	242.306181	204.897875
2565.13665	1730.38535	1254.81665
652.819645	1017.76337	546.526059
120.502425	183.364406	144.944574
93.5562123	93.0479379	60.4957822
303.05938	356.78208	211.461221
86.1985466	101.264349	69.627751
191.77455	234.91529	209.791329
85.1789852	61.35875	89.4541016
54.8762582	51.4141759	60.9933641
350.041087	463.611762	468.172762
19.755208	22.8055767	30.7106022
214.557923	228.385379	202.973232
46.1303423	44.1241226	52.9304769
259.219039	353.697327	399.667802
53.1524924	61.8640061	43.7667436
54.3410471	50.8728773	67.889605
90.8572517	86.905848	98.0852787
249.618005	323.363488	141.933754

P7 N2 | P7 N2 002 | Geometric Segment	P7 N2 | P7 N2 003 | Geometric Segment	P7 N2 | P7 N2 004 | Geometric Segment
455.327042	487.957513	388.059555
488.8961	314.49722	280.270662
219.361887	220.265673	247.800631
384.134994	406.88714	464.241044
548.292137	537.858317	519.324587
751.968667	779.100464	648.117453
169.384615	159.73959	179.439756
1199.41073	1155.21152	1181.54025
4814.08261	6788.88088	8047.46957
227.756399	224.138367	226.741313
71.7156902	58.2214876	66.8707864
4016.78202	4766.62217	4844.43391
10502.8118	9893.7952	9391.13519
354.081521	337.505852	307.972657
1424.22567	1007.90716	1212.22948
218.036667	270.330035	272.455479
215.247309	158.134899	201.029166
30.9928021	31.7542645	33.6387811
560.184593	249.108755	241.496611
65.6149354	95.0525663	64.1156774
1230.45368	1051.05203	920.64831
783.097599	534.950654	442.801478
110.084208	122.965966	145.452499
191.137234	279.92951	296.140694
802.053602	1164.95797	940.069511
513.773781	597.225734	405.542267
73.1198943	113.138684	81.570751
59.8554641	74.8161016	69.7657399
122.265936	139.484912	193.156554
69.6350307	91.5803039	75.2329339
180.886579	166.403384	141.314187
81.5855993	55.1488258	54.6631232
98.9003819	50.8639841	39.7890099
452.597258	456.589718	446.14861
34.0728852	23.3971426	24.219372
242.101765	158.560567	180.801461
27.8472315	31.2197804	30.279869
454.693403	399.486414	361.92072
49.2282214	87.9593802	35.2371245
64.7087459	71.4120296	98.1488595
109.694522	112.731217	90.7763375
774.604552	846.95681	483.275441

P7 N2 | P7 N2 005 | Geometric Segment	P7 N2 | P7 N2 006 | Geometric Segment	P7 N2 | P7 N2 007 | Geometric Segment
289.787925	273.096587	165.999181
319.323799	809.314978	330.554035
259.84532	326.619845	221.293385
450.266126	1305.67585	626.727827
478.542409	761.964084	288.415319
519.732564	959.080543	220.101362
197.155225	250.674234	207.067296
1185.73529	1308.81322	774.209945
7662.16403	4371.24022	843.519727
287.664301	615.397869	695.128283
66.2031037	63.1147479	38.7973205
4729.87338	2184.57539	559.216666
12436.858	3863.67825	1146.72069
329.598806	460.794596	298.412419
1317.98475	1026.11419	499.29559
274.556453	396.621824	281.747104
213.531367	163.964211	121.334702
36.8757184	91.8219666	109.570396
199.70926	542.909188	795.240805
53.1055325	126.548206	32.3433407
852.731233	260.487133	85.0478213
564.838241	584.625312	215.168965
119.602818	65.8908429	10.0645846
251.761398	193.464775	166.548305
1025.09846	3482.36418	3475.65458
217.311917	195.942564	52.4071796
66.1911352	121.82804	42.9123098
70.2980344	66.576006	52.4351478
145.473914	363.152443	209.394903
61.4567796	156.362274	75.853398
135.159207	310.436578	104.319708
58.7325274	59.7927935	41.6209779
54.4952652	52.8873844	23.6965052
414.327828	483.325806	414.244589
32.8377639	43.1866077	40.0545307
152.678316	117.254595	99.1855042
45.7946355	47.3425001	32.9153074
351.612686	585.874079	839.412171
44.2431594	329.313484	233.528679
56.7812115	1915.11184	3384.2268
81.805721	3366.15877	8466.88359
132.57611	6266.71367	15822.6347

P7 N2 | P7 N2 008 | Geometric Segment	P7 N2 | P7 N2 009 | Geometric Segment	P7 N2 | P7 N2 010 | Geometric Segment
291.093489	175.66289	237.201636
524.449065	622.78259	448.243308
245.457434	231.054988	321.199891
707.339072	817.796532	566.832092
606.912548	579.67601	423.164411
803.91246	801.564308	377.501299
263.547489	228.241195	398.0764
1308.45579	1085.76512	911.94727
3212.43079	3554.05356	3952.92499
936.106361	697.968755	303.516645
52.2503856	29.304994	32.3019503
962.174356	1480.82704	2461.52071
2767.05473	2031.79641	12585.6801
354.435387	374.493468	336.059443
740.111467	821.356589	783.737885
273.364118	347.028155	257.22077
164.665926	115.034883	169.143966
97.3955503	93.0057889	26.6704551
819.608015	1053.4447	110.951281
85.0751419	156.32339	27.2672876
342.233586	135.845103	426.129127
541.367625	420.612734	194.961103
38.1220419	32.5805923	74.3809902
237.487987	205.429736	241.154814
3575.35411	4485.64941	285.908025
237.527151	74.3125075	151.152293
94.9711125	62.8704146	53.5921575
61.7900173	48.093157	41.0662007
297.069792	360.787495	66.9192315
99.7614725	74.3361213	95.1934575
224.292205	188.63005	187.463275
60.4021046	52.2672829	33.4896693
40.5149644	33.9597994	88.4721879
497.634404	512.712295	349.939605
38.4787454	37.5833658	83.1066911
160.110998	108.125775	138.328429
46.7529997	41.0668626	33.6102856
601.849748	527.299601	477.051513
255.004309	331.161603	306.127795
2526.8756	2892.63357	77.8171702
4875.76797	5636.33207	95.9625035
6745.51185	6239.02239	141.327452

P7 N2 | P7 N2 011 | Geometric Segment	P74-3 | P73 03 006 | Geometric Segment	P74-3 | P74 03 001 | Geometric Segment
305.5411	254.453908	244.603924
364.535512	359.074477	325.998234
302.64539	263.674433	241.058244
539.651599	483.829894	517.577729
423.627956	390.656048	557.42277
384.231281	352.031694	680.907171
239.672185	239.565669	202.664922
1202.10104	823.891561	1245.80602
4346.72237	3678.73911	7503.32193
317.559456	307.136743	248.997392
49.1448855	34.5174762	32.6932201
3066.44505	2686.38568	2937.92296
8842.2523	9469.41289	8067.94985
315.63293	303.243267	230.817529
902.939609	842.301802	846.273916
247.996022	246.759053	271.764865
145.759612	163.870079	154.731943
26.6391271	27.758333	44.2568034
146.686645	148.596712	224.20742
48.744923	44.3807232	73.9037336
532.369291	548.523996	514.266384
273.196325	209.022423	284.048863
61.5306116	62.057927	94.9906929
197.497824	247.86944	213.424503
238.126025	199.893756	628.216281
161.468842	116.980483	230.682332
60.5084928	59.6820081	106.872648
38.6450229	40.6744827	51.330017
103.446635	88.8270915	138.716903
112.667273	81.2091802	57.3752441
195.146192	187.400325	187.632845
44.4513759	34.8120761	50.1242779
50.6109157	52.0041775	35.2344603
301.571897	266.510267	311.274429
72.3458506	67.8850139	55.1627104
136.126371	116.658007	136.789955
40.7236764	38.4235391	28.6609807
387.00657	344.443571	235.324623
84.697039	111.74793	284.218052
61.8273119	55.7892222	68.1516145
76.6926403	75.4700935	83.5227518
60.0494088	85.0100075	310.991234

P74-3 | P74 03 002 | Geometric Segment	P74-3 | P74 03 003 | Geometric Segment	P74-3 | P74 03 004 | Geometric Segment
295.804394	232.044142	251.579346
671.406548	405.20867	391.784678
484.39114	324.09697	308.666144
860.690895	562.327394	515.454214
551.177535	413.804297	379.757392
419.06222	348.21175	344.893797
335.655429	274.076921	283.289105
1187.34328	1064.04614	792.453081
7112.97869	4031.70001	3720.14737
464.832129	350.885884	334.308124
50.7506998	39.4477903	30.8885771
3138.0852	2501.11323	1762.46592
2437.63134	10906.8768	12234.0351
688.184875	309.417543	297.898905
882.820656	819.258591	690.156403
398.021023	284.585394	242.841922
232.188688	173.562109	152.017294
58.6141266	29.9683023	31.6417501
176.149709	156.613224	81.6636317
64.1805273	32.1708808	29.9711664
374.839441	503.485887	328.897265
209.517848	209.257306	203.109112
106.014069	44.6459739	42.2142737
221.45435	209.004907	185.472228
751.067371	276.649767	177.237394
69.2507127	102.026141	128.27745
80.7105849	59.3623197	54.4281939
59.0739766	40.1351685	41.1651853
56.3719081	109.387307	46.3389047
159.979445	97.9725113	60.9357117
430.825214	222.029559	193.125556
43.6409645	44.3910923	32.7205083
134.581429	102.012755	71.2267556
751.099778	305.264875	261.00875
201.449528	103.237018	74.8433468
197.066795	170.196878	109.077195
60.754714	31.259231	29.4176926
988.829814	440.345593	441.563701
1649.39843	157.327209	105.52689
88.2891568	66.1703534	75.2379562
182.365152	75.599901	68.2506889
527.47612	104.826664	94.5558571

P74-3 | P74 03 005 | Geometric Segment	P74-3 | P74 03 007 | Geometric Segment	P74-3 | P74 03 008 | Geometric Segment
346.766069	273.018525	194.951932
311.693436	336.187923	363.928654
215.493226	252.693009	299.905727
432.120554	469.455603	524.076894
510.523136	406.631015	396.239032
692.859817	407.594266	266.892812
180.2681	214.090262	248.743898
1102.52772	935.646739	738.618014
7133.49213	3878.39289	3305.52082
210.578181	293.094578	303.393754
44.9729301	43.3962883	27.7237807
3360.75176	2283.07238	2799.51825
4833.27177	6726.98216	11338.9696
218.984742	283.757147	307.430939
865.479159	643.306524	849.584256
245.299123	218.987321	248.350257
146.073465	148.774572	143.926732
34.8311149	20.0480824	19.0626919
240.585333	99.3976797	111.023307
56.414635	44.1467798	30.0561202
453.770884	442.283706	379.078391
242.218827	201.033236	131.768506
95.2605327	66.8539527	61.0552651
239.204181	210.236305	243.051589
426.293165	259.115353	186.207489
271.79754	211.228451	110.22926
105.857987	67.9300142	47.6291968
47.729637	30.6534418	29.5188153
218.823091	58.3799602	96.9089655
95.3953992	67.4947379	73.2890636
166.897241	182.725529	198.59123
44.2333779	30.8315793	33.2650021
27.243828	56.1500213	57.7655775
199.807712	268.621205	253.037565
35.4666125	71.9565541	65.7601806
106.148563	106.218501	126.429027
33.0170623	30.042352	31.5300977
243.627991	349.483696	366.395655
124.846011	138.135596	69.5469921
49.9224118	71.5249881	60.0737032
57.7155675	80.5394889	81.4180853
87.9563202	73.3698801	85.7449952

P74-3 | P74 03 009 | Geometric Segment	P74-3 | P74 03 010 | Geometric Segment	P77 | 001 | Geometric Segment
397.31522	321.564998	423.088745
710.928761	881.982262	741.228317
269.348041	272.954675	262.255537
636.706211	591.248896	800.006848
967.833183	482.63709	1266.28823
1076.6145	527.154567	1455.27184
219.596296	218.562872	221.274365
2380.55367	2107.48432	3867.68274
9951.67776	4344.37178	11000.3889
280.793067	272.568366	249.949371
31.1121573	42.0057035	43.6413826
5907.68944	2287.72778	5565.73764
3121.96473	2085.15692	4249.31358
345.501494	438.809191	346.447431
1056.56944	704.432367	1236.00166
377.138577	291.458916	369.779309
209.669796	175.867273	219.392917
56.8674737	39.5577005	55.3683747
377.789451	207.784427	249.565112
93.9185524	77.7190912	100.13974
1255.43813	982.091342	837.477594
322.350584	334.151217	429.719286
89.8836965	43.7125348	87.5996887
204.461722	212.220929	229.139995
1397.76211	559.306268	1763.47688
229.096279	140.701246	342.450882
115.889884	79.566987	101.583705
60.3577507	53.9096214	70.2589106
370.197678	168.000445	259.23201
140.19749	141.209599	302.390525
170.678643	227.083474	223.595679
54.2826917	43.2217892	48.5431659
58.5082956	98.1641235	63.7254882
466.596217	579.32842	511.275161
39.114902	114.66898	40.4094532
228.487773	189.249028	204.027745
40.6559753	49.3805869	45.126608
266.99319	535.298946	294.806358
327.048378	779.719929	334.954019
51.8512181	49.5429172	63.7639341
38.1915036	64.6891277	46.8116029
120.940037	55.9724642	193.634686

P77 | 002 | Geometric Segment	P77 | 003 | Geometric Segment	P77 | 004 | Geometric Segment
280.297989	410.801468	580.484308
393.768806	694.148494	550.439578
271.353524	307.586763	314.892916
478.932366	653.492313	739.416983
389.460803	611.154964	798.992759
379.528071	744.084747	948.505708
245.893889	203.219265	210.972042
1623.107	2528.43144	3271.29555
4025.13635	12591.3456	10198.0027
263.811517	310.638156	320.448582
23.0657661	39.9909996	52.3979899
2395.21475	5961.01906	6502.6446
5345.72887	4029.78827	2649.98842
291.618024	334.65669	368.992812
919.530743	1120.20872	819.96467
230.313482	376.781406	389.802534
167.621203	250.65808	250.105162
29.0201899	42.4183318	42.4610743
165.847852	312.372237	195.159426
63.2797041	82.7278074	93.0712343
810.313472	1290.82785	825.432406
425.268782	379.114432	448.70867
46.86444	83.196526	100.591444
161.162254	195.656678	236.452743
410.661776	1163.64889	1436.07395
104.781583	252.205211	252.053175
45.31785	90.2257691	108.912788
44.1764617	53.3526017	58.7319964
210.624415	322.114952	154.879661
69.8496938	111.831362	137.540243
133.406082	148.583787	185.626015
37.980864	62.3836395	45.1927694
68.2291031	44.8473252	88.8750118
358.423678	430.571233	463.994032
63.9880647	30.3253227	38.253131
156.897905	237.739859	169.863403
35.0000081	45.7783293	41.705405
281.382292	278.35605	310.074759
110.438819	158.249318	416.254918
61.3548933	53.7250518	44.4955483
38.2926474	37.960586	44.1601
70.2033466	43.1600699	48.9215119

P77 | 005 | Geometric Segment	P77 | 006 | Geometric Segment	P77 | 007 | Geometric Segment
467.521757	279.333034	254.39173
730.224029	569.796312	402.758931
306.742751	303.960751	267.357807
671.425905	749.486331	611.273294
818.442705	529.410739	399.219021
1067.78039	483.835378	375.538053
203.787776	209.37811	227.209204
3907.7836	2458.76802	1541.07691
12043.2663	9924.47542	8102.60509
305.411346	331.82822	250.440919
34.2623301	29.0695798	28.2775849
5633.5556	6106.8052	3578.59464
4367.55105	5109.35537	5371.12027
374.931044	364.762996	315.082136
1263.5195	1549.54358	1155.06277
392.465107	368.689071	246.040741
274.917186	232.635097	201.966806
50.0630947	60.8445467	41.3284509
329.410276	240.26476	125.607525
86.567655	47.648477	41.3062439
1132.53167	765.795944	487.16255
389.040837	243.241036	210.994412
85.9166534	73.2292109	60.9618381
225.511636	238.554493	222.960077
1464.37701	976.299401	746.867568
308.90285	102.279412	80.8851858
104.60876	110.934671	78.6000197
61.7311016	48.13474	39.897211
260.940865	288.692552	137.546294
168.989161	118.940438	71.7035557
198.352515	134.921482	112.094801
65.8647702	63.5101314	34.8776945
47.3562343	48.2238994	48.1822287
444.277513	371.47499	357.592999
29.2028074	36.9584497	28.6525156
267.525082	241.939362	137.419969
44.7221907	42.7899623	31.4290283
283.713852	253.250474	254.611336
241.897891	149.53228	131.954763
53.7317198	51.5875101	49.7240839
42.5372457	36.2399701	40.6445317
107.377027	79.5124903	59.1826857

P77 | 008 | Geometric Segment	P77 | 009 | Geometric Segment	P77 | 010 | Geometric Segment
198.313135	308.964212	361.066959
424.324369	460.776525	781.489472
268.75722	255.445129	321.762638
470.740641	562.769462	733.156748
313.886452	485.251864	568.085014
228.8351	462.028876	609.320904
195.196529	230.930639	223.811245
1134.23943	1749.18912	2580.21513
3615.8743	5645.56399	6822.98036
263.418769	281.171843	303.448061
24.3486226	35.9140492	45.4050893
2117.51665	3426.02799	3661.7013
6390.35935	5876.67794	4400.59598
300.099604	328.542149	327.345599
811.846843	833.658594	1302.43528
221.915672	239.626702	318.916086
156.786743	186.501036	174.723389
28.9247169	36.5012139	43.6529692
81.8238527	202.280001	313.672612
29.0791287	42.746118	72.4220538
508.989218	931.309662	1198.55654
199.049921	332.254223	460.471874
38.3567862	53.8249613	68.846852
172.123231	190.102694	206.464248
345.476834	690.918139	1006.92309
49.4576291	135.486343	160.554952
42.8266757	59.5719228	69.879945
34.3187323	44.5910058	60.9460687
152.666768	218.981245	376.44397
60.2823606	86.9090339	104.914148
117.098146	177.705588	162.808408
36.5576978	38.8894526	51.5899708
46.5677846	51.7795505	57.5016192
283.878142	365.613955	444.660431
57.4749056	47.2043095	45.1625149
140.53352	194.850389	224.357934
27.8446066	38.4196807	46.8850255
271.293184	332.260518	295.763592
64.690829	127.463244	174.594518
65.0540906	52.9477731	64.4074544
41.8334752	39.3300837	42.8364528
45.5375845	102.34871	73.372685

P78 N1 | 001 | Geometric Segment	P78 N1 | 002 | Geometric Segment	P78 N1 | 003 | Geometric Segment
146.941958	181.737798	340.209138
578.021516	588.854307	487.346963
233.759743	178.175957	209.152867
855.086892	1219.22121	336.690908
896.794194	760.095384	228.630011
1397.17024	1385.73228	316.85293
157.409602	192.125607	182.303552
12118.1174	7466.54914	2546.49072
4738.98402	5231.21715	4584.96629
296.92688	271.135582	181.641025
55.9453332	74.4336199	49.0395177
6453.13993	4789.22714	3164.90578
536.099393	970.4035	8462.11967
732.553033	560.56629	341.801609
1626.14419	1583.3751	1219.40163
286.142976	254.145369	144.434985
89.0458943	82.0197295	111.505987
194.56344	216.736435	30.4425294
117.609387	115.122354	206.975261
298.200806	210.197739	68.3867518
275.18979	425.608278	982.440575
1802.91623	1109.27687	417.905638
42.3953801	37.6369289	41.8643022
112.446271	123.279503	178.725034
3689.15013	3194.40841	541.398332
128.518781	281.447042	155.665354
94.9102621	134.778592	66.5780021
83.7859383	100.405208	38.8610221
32.0601655	26.4133547	41.7717041
75.3230233	73.3669983	53.4335257
141.705625	114.930347	84.2815769
41.1322872	33.763458	37.3190677
40.2082874	33.1263342	28.9512757
304.147138	265.995264	122.797025
21.1688074	8.54219256	12.4426371
85.6953623	90.9156626	84.4232319
9.90894418	17.3269629	13.9783099
158.022892	128.44147	101.139223
465.576504	502.275578	123.460753
59.9173809	51.9621389	35.0329788
68.2435832	64.2556871	42.4044606
3269.54821	4038.87663	28.3784223

P78 N1 | 004 | Geometric Segment	P78 N1 | 005 | Geometric Segment	P78 N1 | 006 | Geometric Segment
360.747494	257.880568	414.989471
498.774879	462.715716	454.829213
150.803696	181.87449	142.384053
295.017495	319.652519	346.142735
224.519869	260.29625	349.0043
430.31526	480.053742	495.430108
140.386338	148.496282	140.697698
2148.59351	2281.12364	2233.51059
5468.75201	3658.73551	4717.04139
169.146012	167.49393	150.575371
51.2880623	23.7769963	60.1368156
2998.91719	2034.5822	2431.86232
10394.4765	9087.58031	3588.17604
340.016133	333.959511	336.321637
898.025291	821.505774	694.238133
176.68456	183.537843	173.665433
99.3794347	92.0728545	127.697857
27.6342703	22.1119549	26.0343264
172.225377	73.4180348	152.353027
31.8690318	31.3563699	44.3022957
931.745467	335.956791	731.204521
291.850921	407.418423	444.068643
43.6780929	33.2891882	55.5251942
144.978662	239.434407	114.642155
373.625048	647.629318	510.20909
274.472064	202.123283	425.022206
58.0061899	70.275677	79.0912954
46.5521368	52.4108525	58.0053811
46.5361598	45.2643579	54.0890907
38.8917063	43.7034189	74.0652152
74.6012554	67.4902473	83.8766975
30.9400248	19.9710183	18.8108925
18.4305377	15.2876246	29.9990821
128.796888	120.710547	153.724899
7.39297965	14.8847407	11.6833994
84.093996	72.2615352	62.2535022
11.2469266	3.07691466	9.66058419
99.0550355	107.90089	76.7830803
82.2344721	219.808209	141.647359
32.7649349	37.5152068	30.517395
36.6012034	32.2831234	22.8058364
40.5323696	61.4200038	44.8149972

P78 N1 | 007 | Geometric Segment	P78 N1 | 008 | Geometric Segment	P78 N1 | 009 | Geometric Segment
415.237962	328.891767	458.959428
430.226161	508.127206	421.122893
157.636804	231.663715	192.028529
286.40326	451.26511	332.28734
281.451416	306.492671	317.946197
510.005169	392.006897	499.499416
126.835336	195.600322	163.772926
1984.76118	2501.8925	2233.46496
5052.06004	4223.69132	4782.75614
179.067531	267.980934	175.635161
33.7794189	48.2053609	52.9184823
2645.38002	3089.76177	2296.84236
5767.50807	8847.31375	5086.41269
267.142255	424.125495	346.556341
842.399735	1079.5928	724.37846
173.424607	225.301135	165.594298
88.2151087	130.034851	130.33336
27.9005332	30.7403413	20.9124347
107.45982	135.078969	203.765852
48.8345111	46.8614571	41.9737439
614.299255	974.970453	1122.327
349.163444	659.980604	498.153492
39.2035453	29.2458097	50.857687
118.42898	212.617505	130.061102
414.352734	552.618686	545.150037
369.661222	222.281201	375.263343
82.4135396	59.0858628	64.2078462
41.2658863	54.8288672	57.6510367
50.4648087	71.4974268	92.2037324
56.1469427	74.1052373	64.0703689
68.1265459	77.5366502	79.2407829
22.8472125	12.9565249	21.0185518
11.1451574	22.7289715	16.4355124
133.453236	145.837879	140.507436
8.58575668	37.9371109	17.3258825
56.9309795	102.909191	98.7297776
1.03530794	8.73336948	3.13384558
59.845154	139.908779	91.7823913
75.786365	161.750104	107.791779
24.0983912	30.6998305	31.956899
38.0187371	37.9613709	33.97646
27.0700174	41.0398434	84.5833467

P78 N1 | 010 | Geometric Segment
142.15215
582.633001
178.533269
317.143382
188.433878
194.275454
169.051035
1634.90023
4895.42934
197.163697
17.2664285
3415.4585
15285.1517
364.202094
1077.99869
187.94116
126.215542
21.2902663
48.3582448
21.9572039
143.504168
227.387416
26.0626063
219.079947
494.849061
69.4624264
40.5261557
60.3723919
34.7712383
52.7441307
75.8126651
24.4731298
15.6116235
111.696655
11.291578
51.8350921
2.5137003
90.0875195
86.4612565
35.9420146
31.6486244
46.6437514
